# A highly biocompatible self-assembled Pt(IV) nanoplatform integrating Nrf2 inhibition for synergistic apoptosis and ferroptosis in hepatocellular carcinoma

**DOI:** 10.1016/j.mtbio.2026.103138

**Published:** 2026-04-20

**Authors:** Xi Chen, Siqian Cui, Rongzhen Deng, Hongwei Zhang, Zhengwen Zhang, Yiguo Zhang

**Affiliations:** a The Laboratory of Cell Biochemistry and Topogenetic Regulation, College of Bioengineering, Chongqing University, Chongqing, 400044, China; b Department of Oncology, Chongqing University Jiangjin Hospital, Chongqing, 402260, China; c College of Chemistry and Chemical Engineering, Chongqing University, Chongqing, 400044, China; d Laboratory of Neuroscience, Institute of Cognitive Neuroscience and School of Pharmacy, University College London, 29-39 Brunswick Square, London, England, WC1N 1AX, United Kingdom; e Department of Laboratory Medicine, Chongqing Center for Clinical Laboratory, Chongqing Academy of Medical Sciences, Chongqing General Hospital, School of Medicine, Chongqing University, Chongqing, 401147, China

**Keywords:** Hepatocellular carcinoma, Glycyrrhetinic acid, Pt(IV), Nrf2, Apoptosis/ferroptosis

## Abstract

The clinical efficacy of cisplatin (CDDP), a first-line chemotherapeutic agent for hepatocellular carcinoma (HCC), is limited by dose-limiting systemic toxicity and intrinsic or acquired resistance. To address these drawbacks, self-assembled Pt (IV) prodrug nanoparticles, termed **DGA** NPs, were constructed via conjugation of CDDP with 18β-glycyrrhetinic acid (GA). In vitro, **DGA** NPs demonstrated superior antiproliferative activity over CDDP against a range of cancer cell lines, including a CDDP-resistant model. Furthermore, they effectively inhibited the migration and invasion of HepG2 cells. Mechanistically, **DGA** NPs induced DNA damage, mitochondrial dysfunction, and reactive oxygen species (ROS) accumulation. These cellular stresses led to the sustained activation of the p53 and AMPK pathways, thereby driving robust apoptosis. Notably, through inhibiting the Nrf2 antioxidant axis, **DGA** NPs suppressed the PI3K/AKT/mTOR survival pathway and triggered ferroptosis in HCC cells. In a HepG2 xenograft model, **DGA** NPs demonstrated significantly superior antitumor efficacy relative to CDDP monotherapy or a CDDP/GA combination, without inducing detectable systemic toxicity. Collectively, **DGA** NPs represent an innovative Pt (IV)–based nanoplatform that simultaneously activates both apoptotic and ferroptosis pathways, offering a promising dual-mechanism strategy for the treatment of HCC.

## Graphical abstract

A novel Pt (IV) nanoplatform integrating cisplatin and 18β-GA induces dual apoptosis and ferroptosis by disrupting redox and survival signaling, thus overcoming cisplatin resistance and minimizing systemic toxicity in HCC.

**Figure:**
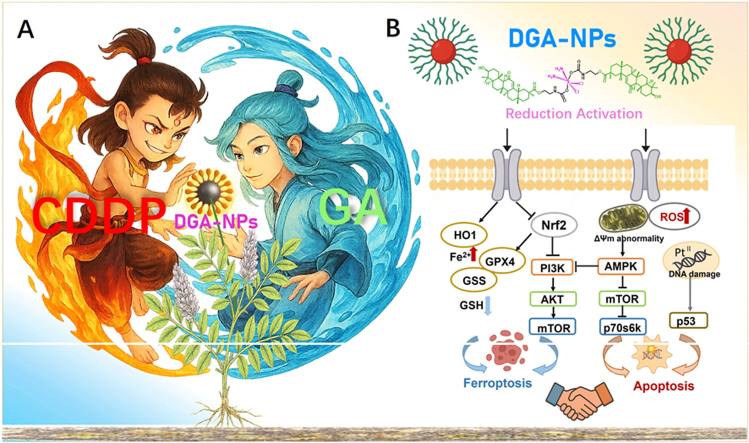

## 1 Introduction

**Scheme 1:**
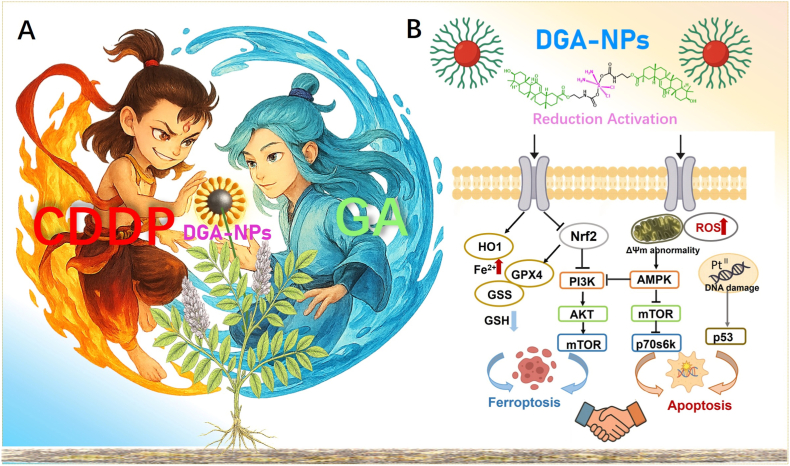
**Schematic illustration of DGA NPs for HCC therapy. (A)** Synthesis of the **DGA** conjugate and its self-assembly into nanoparticles (**DGA** NPs). **(B)** Upon endocytosis and intracellular release, **DGA** NPs induce redox imbalance, activating a dual cell death pathway: p53/AMPK-mediated apoptosis triggered by DNA damage, and Nrf2-suppression-driven ferroptosis via PI3K/AKT/mTOR inhibition.

Hepatocellular carcinoma (HCC) is a major public health burden, characterized by its high global incidence and mortality, as well as a poor prognosis [[1], [2], [3]]. The conventional Pt (II)-based chemotherapeutics, such as cisplatin (CDDP), remain cornerstone agents, but are severely hampered by dose-limiting systemic toxicity, intrinsic lack of tumor selectivity, and frequent evolution of acquired resistance. Thus, developing novel strategies that enhance therapeutic efficacy while minimizing off-target effects and overcoming resistance is a critical unmet need for HCC treatment (see Scheme 1).

To address these limitations, Pt(IV) prodrugs have emerged as a promising next-generation platform in anticancer drug design due to their favorable pharmacological properties. Unlike their counterparts of Pt(II), these complexes are chemically inert under physiological conditions, which improves pharmacokinetic stability and reduces systemic toxicity. Upon entry into tumor cells, they are reductively activated to release cytotoxic Pt(II) species, enabling more precise therapy. The novel multifunctional Pt(IV) prodrugs represent a core direction in current research and development of such platinum-based anti-tumor agents. This design strategy primarily involves the functional modification of the axial coordination sites of Pt(IV) complexes, so as to construct the coordinated drug delivery systems with multiple biological effects [[4], [5], [6], [7], [8]]. For instance, Meng W et al. developed a multi-target platinum(IV) antitumor prodrug by conjugating an NF-κB inhibitor with CDDP. This prodrug effectively induces DNA damage, promotes reactive oxygen species (ROS) generation, stimulates autophagy, and triggers the mitochondrial apoptotic pathway in lung cancer cells. It also suppresses cell proliferation via inhibition of the NF-κB signaling pathway. Furthermore, it significantly reduces the levels of VEGF and HIF-1α in drug-resistant lung cancer cell lines and exerts antiproliferative effects through the PI3K/AKT and STAT-3 pathways [9]. Jin et al. developed a novel Pt(IV) prodrug by conjugating CDDP with a lactate dehydrogenase inhibitor. This agent not only exerts direct cytotoxicity but also modulates glucose metabolic reprogramming in tumor cells, demonstrating the potential for synergistic metabolic regulation and chemotherapy [10]. As reported by B. Yu et al., an amphiphilic polymer incorporating both a COX-2 inhibitor and a cisplatin prodrug has been developed. This system concurrently suppresses COX-2 expression, enhances the immunosuppressive tumor microenvironment, and induces DNA damage. These combined effects substantially amplify pyroptosis, thereby eliciting a sustained immune response and offering a generalizable therapeutic strategy for pancreatic cancer [11]. These advances illustrate that the Pt(IV) platform can be tailored to overcome specific mechanisms of resistance and enhance therapeutic precision.

Beyond augmenting platinum-based cytotoxicity, a complementary and promising strategy to overcome therapeutic resistance involves inducing ferroptosis [12,13]. This process is centrally regulated by the nuclear factor erythroid 2-related factor 2 (Nrf2, encoded by nfe2l2), a master activator of cellular antioxidant defenses. Inhibiting Nrf2 disrupts this protection, sensitizing cancer cells to ferroptosis [14,15]. Notably, conventional therapies like CDDP can prime cells for ferroptosis, and combining them with ferroptosis inducers presents a promising avenue to overcome therapeutic resistance [16]. Consequently, the development of Nrf2-targeted inhibitors to disrupt tumor antioxidant defenses and induce ferroptosis has gained considerable attention as a promising anti-tumor strategy. In this context, natural products are a valuable source for discovering effective Nrf2 modulators. The licorice-derived pentacyclic triterpenoid 18β-glycyrrhetinic acid (GA) has been shown to regulate the Nrf2 signaling axis [17,18]. Moreover, GA possesses inherent liver-targeting properties due to its specific affinity for receptors overexpressed on HCC cells [19]. This dual functionality makes GA an attractive candidate for therapeutic development. Integrating GA into a platinum-based delivery system thus represents a logical strategy to co-deliver a cytotoxic agent and an Nrf2 inhibitor directly to tumors, with the goal of achieving synergistic efficacy through coordinated induction of complementary cell death pathways.

Building on this rationale, a novel Pt(IV) prodrug was synthesized via the conjugation of CDDP with 18β-GA. This amphiphilic prodrug subsequently self-assembled into nanoparticles, designated as **DGA** NPs, which are designed to leverage the tumor-targeting capability of GA for enhanced selective drug accumulation. We hypothesized that this nanoplatform would leverage the tumor-targeting capability of GA to enhance selective drug delivery while executing a synergistic multimodal attack. Upon reductive activation within the tumor, it would simultaneously: i) release CDDP to induce classic cytotoxic effects (DNA damage, ROS generation, and apoptosis), and ii) concurrently deliver GA to inhibit the Nrf2-mediated antioxidant pathway, thereby sensitizing cells and triggering ferroptosis via suppression of the PI3K/AKT/mTOR axis. Thus, by co-delivering a cytotoxic agent and an Nrf2 inhibitor, **DGA** NPs represent an innovative approach to enhance efficacy and counteract resistance in HCC, through the simultaneous activation of complementary cell death pathways.

## 2 Results and discussion

### 2.1 Preparation and characterization of **DGA** NPs

**Fig. 1:**
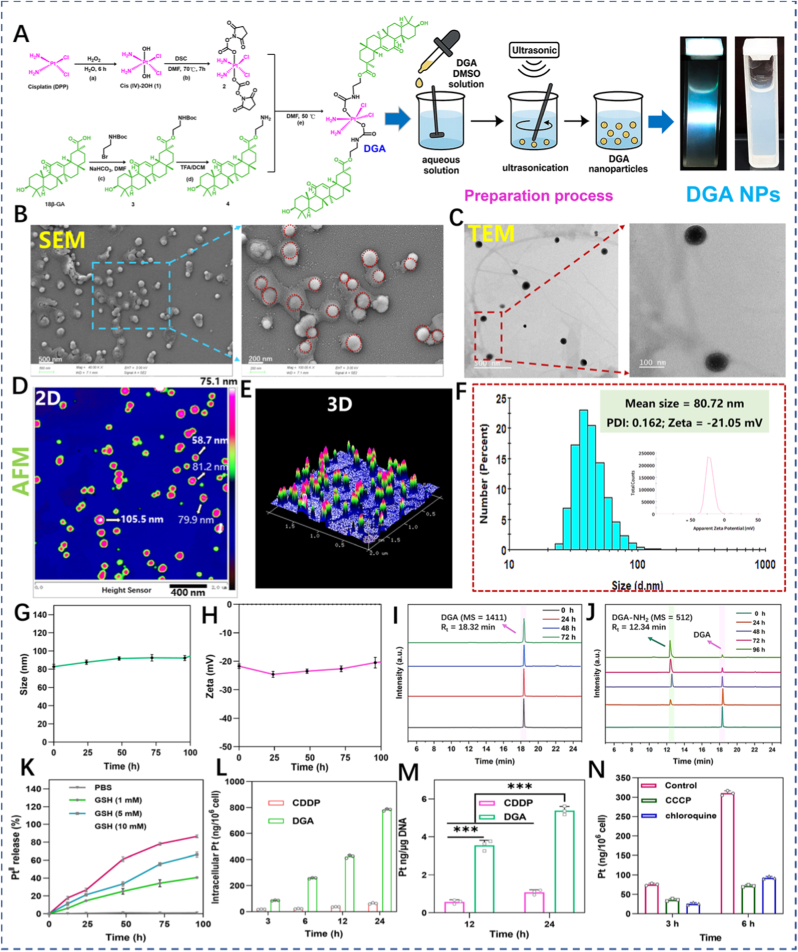
**Preparation and characterization of DGA** NPs. **(A)** Synthetic route to **DGA** NPs. Reagents and conditions: a) 60 °C, 6 h; b) DSC, TEA, 70 °C, 8 h; c) NaHCO_3_, DMF, 40 °C, 12 h; d) TFA/DCM, rt, 2 h; e) TEA, DMF, 50 °C, overnight. **(B)** Representative SEM image (left), **(C)** TEM image, **(D)** 2D and **(E)** 3D AFM images of **DGA** NPs. **(F)** Size distribution measured by DLS (PDI < 0.2). Inset: Zeta potential. **(G)** Size and **(H)** Zeta potential changes of **DGA** NPs in PBS (pH 7.4) over 120 h at 25 °C. **(I)** Stability of **DGA** NPs in PBS (pH 7.4, 72 h). **(J)** Reduction profile of **DGA** NPs in PBS containing 10 mM GSH at 37 °C for 0-96 h. **(K)** Reduction kinetics of **DGA** NPs by GSH (1, 5, 10 mM) in PBS (pH 7.4). **(L)** Intracellular platinum content in HepG2 cells after treatment with CDDP or **DGA** NPs for 0-24 h. **(M)** Pt-DNA levels in HepG2 cells after 12, 24 h of treatment with CDDP or **DGA** NPs. **(N)** Intracellular platinum content in HepG2 cells incubated with **DGA** NPs at 37 °C for 24 h. Cells were pretreated with CCCP (30 μM) or chloroquine (30 μM) for 2 h prior to incubation. Data are presented as mean ± SD (n = 3). ∗p < 0.05, ∗∗p < 0.01, ∗∗∗p < 0.001 (one-way ANOVA); NS, not significant.

The synthetic route for **DGA** NPs is illustrated in Fig. 1A and Scheme S1. Initially, the Pt(IV) core scaffold (**1**) was obtained by oxidizing CDDP with 30% H_2_O_2_. XPS analysis of intermediate 1 (Fig. S1) showed characteristic peaks at 74.9 eV and 78.25 eV, assigned to Pt 4f_7/2_ and Pt 4f_5/2_, respectively, confirming the Pt(IV) oxidation state. Subsequently, the target **DGA** conjugate was synthesized via a N, N′-disuccinimidyl carbonate (DSC)-mediated coupling reaction between the intermediate **2** and amino-functionalized derivative of 18β-glycyrrhetinic acid (GA-NH_2_, **4**). Following purification, the final **DGA** conjugate was isolated with a yield of approximately 40% (calculated based on the Pt(IV) precursor, intermediate **2**). The structures of all intermediates and the final product were confirmed by comprehensive characterization, including ^1^H, ^13^C and ^195^Pt NMR, FT-IR, and ESI-HRMS (Figs. S2–S20). HPLC analysis indicated a purity of >95% for the **DGA** conjugate (Fig. S21 and Table S1). The amphiphilic **DGA**, comprising a hydrophilic Pt(IV) center and a hydrophobic GA moiety, readily self-assembled into nanoparticles using a reverse solvent precipitation method. Briefly, a DMSO solution of **DGA** conjugate was added dropwise into ultrapure water under vigorous stirring. Subsequent dialysis to remove the organic solvent yielded carrier-free nanoparticles, designated as **DGA** NPs. The formation of NPs was visually indicated by an opalescent dispersion and a distinct Tyndall effect upon laser irradiation. Morphological analysis by SEM/TEM analyses revealed that the **DGA** NPs were spherical and well-dispersed nanoparticles (Fig. 1B and C). AFM analyses further corroborated these findings, providing consistent height profiles and confirming the spherical shape (Fig. 1D and E). DLS analysis revealed an average hydrodynamic diameter of ∼80.72 nm with a low polydispersity index (PDI <0.2), confirming excellent mono-dispersity (Fig. 1F). The zeta potential was approximately −21 mV, suggesting good colloidal stability. To evaluate stability under biologically relevant conditions, **DGA** NPs were dispersed in PBS (pH 7.4, 25 °C). As shown in Fig. 1G and H, both the particle size and zeta potential remained virtually unchanged over 120 h, demonstrating outstanding colloidal stability. This robust stability is a critical attribute for systemic administration, as it facilitates prolonged blood circulation time. Consequently, it promotes enhanced tumor accumulation through the enhanced permeability and retention (EPR) effect, laying an essential foundation for the subsequent evaluation of its antitumor efficacy.

### 2.2 Stability, reductive activation, and cellular uptake properties

The stability and glutathione (GSH)-responsive drug release profile of **DGA** NPs were systematically investigated under physiological and tumor-relevant reductive conditions. **DGA** NPs displayed excellent stability (PBS, pH = 7.4, 37 °C), with negligible drug release (<2%) over 72 h, confirming their suitability for prolonged systemic circulation (Fig. 1I). In contrast, the NPs were progressively activated when exposed to a reducing environment (10 mM GSH). As confirmed by HPLC analysis, the **DGA** conjugate peak (R_t_ = 18.32 min) diminished with the simultaneous rise of peaks assignable to the released GA-NH_2_ (R_t_ = 12.34 min), corresponding to a conversion yield of ∼60% after 48 h (Fig. 1J). To better mimic the intra-tumoral reductive milieu, its release kinetics were further evaluated under varying GSH concentrations. As shown in Fig. 1K, the release of **DGA** NPs was strongly dependent on GSH concentration. While release was slow and incomplete (only ∼42% after 96 h) at a low GSH level (1 mM), it accelerated significantly at 5 mM, reaching a plateau of ∼67% by 96 h. Ultimately, at a high concentration of 10 mM GSH, the release became nearly quantitative, exceeding 80% within 72 h. Given that GA is conjugated to the Pt(IV) core via a hydrolyzable ester linkage, an hydrolysis assay was performed to verify whether the GA-NH_2_ could be converted to the active GA moiety under cellular conditions. GA-NH_2_ (10 μM) was incubated with HepG2 cell at 37 °C for various time intervals, and the cell lysate cell lysis buffer was analyzed by LC-MS. GA-NH_2_ gradually disappeared with a concomitant increase of a new peak corresponding to GA (Rt = 15.58 min), confirming the enzymatic cleavage of the ester bond. The conversion rate exceeded 75% within 8 h (Fig. S22). These data establish **DGA** NPs as a robust GSH-sensitive prodrug nanoplatform that combines high stability in circulation with rapid, selective activation within the reductive tumor microenvironment, providing a compelling rationale for targeted therapy.

Subsequently, quantification of cellular uptake revealed that **DGA** NPs (5 μM) were internalized more efficiently than CDDP (5 μM), resulting in a 12.3-fold higher intracellular platinum accumulation at 24 h (**DGA** NPs: 783.0 ± 26.5 vs. CDDP: 63.2 ± 14.3 ng Pt/10^6^ cells) (Fig. 1L and M). Mechanistically, the axial organic ligands enhance cellular uptake by increasing lipophilicity and promoting endocytic internalization, while the subsequent reductive activation generates Pt(II) species with high DNA affinity, leading to preferential nuclear accumulation [20,21]. This is supported by the observation that cellular uptake was significantly attenuated by CCCP (an ATP-depleting agent) and chloroquine (an endolysosomal inhibitor) (Fig. 1N), confirming the involvement of an energy-dependent endocytic pathway.

### 2.3 Anti-proliferative activity of **DGA** NPs

The anti-proliferative effects of the **DGA** NPs were evaluated against a panel of cancer cell lines using the CCK-8 assay. The panel included HepG2 (Human hepatocellular carcinoma-2), MHCC-97H (Metastatic human hepatocellular carcinoma), HCT116 (Human colon carcinoma), A549 (Human non-small cell lung cancer cells), CT26 (Mouse colon cancer) and Hepa1-6 (Mouse hepatoma cell). CDDP and GA were used as controls. As summarized in Table 1, **DGA** NPs exhibited potent cytotoxicity against all tested lines, with IC_50_ values of 2.98, 6.64, 2.01, and 2.74 μM for the human lines, and 4.82 and 1.72 μM for the two murine lines. Notably, the IC_50_ values of **DGA** NPs were lower than those of CDDP in all cases except for MHCC-97H cells (Table 1 and Fig. S23). GA showed only weak cytotoxicity (IC_50_ > 80 μM, Fig. S24), consistent with prior reports [22]. Similarly weak antiproliferative activity was observed for GA-NH_2_ (IC_50_ > 40 μM) and Pt(IV)-OH (IC_50_ > 80 μM, Table S2, Figs. S25 and S26), highlighting that the potent cytotoxicity of **DGA** NPs is not attributable to these individual components.

**Table 1:** IC_50_ (μM) of these compounds against different cell lines at 96 h^a^.

Compounds	HepG2	MHCC-97H	Huh7	Huh7/DDP	RF^b^	A549	HCT116	Hepa1-6	CT26	THLE-2	3T3-L1	SI^d^	SI^e^
CDDP	5.26 ± 2.06	5.34 ± 3.56	2.29 ± 0.09	11.45 ± 0.81	5	7.24 ± 2.24	4.60 ± 1.26	3.63 ± 0.54	5.88 ± 1.84	3.61 ± 1.78	4.52 ± 1.34	0.68	1.24
GA	>80	>80	>80	>80	ND^c^	>80	>80	>80	>80	>80	>80	ND^c^	ND^c^
**DGA** NPs	2.98 ± 1.73	6.64 ± 0.59	1.54 ± 0.79	1.18 ± 0.78	0.76	2.74 ± 1.72	2.01 ± 0.43	1.72 ± 0.52	4.82 ± 1.70	11.44 ± 1.74	8.37 ± 0.55	3.83	4.86

a *IC*_*50*_*values are represented as mean ± SD* (*n = 3 biologically independent experiments*).

b RF, resistant factor; RF = IC_50_ (Huh7/DDP)/IC_50_ (Huh7).

c ND, not tested or not calculated.

d SI: Selectivity index, SI = IC_50_(THLE-2)/IC_50_(HepG-2).

e SI: Selectivity index, SI = IC_50_(3T3-L1)/IC_50_(Hepa1-6).

To assess selectivity of **DGA** NPs, the normal human liver cell line THLE-2 and mouse embryonic fibroblast 3T3-L1 served as normal cell models. As shown in Table 1 and Fig. S23, **DGA** NPs exhibited IC_50_ values of 11.44 μM and 8.37 μM in THLE-2 and 3T3-L1 cells, respectively, which were significantly higher than those in HepG2 (2.98 μM) and Hepa1-6 (1.72 μM) cells, with selectivity indices of 3.83 and 4.86. In contrast, CDDP showed poor selectivity, with IC_50_ values of 3.61 μM and 4.52 μM in normal cells and selectivity indices of 0.68 and 1.24. To directly evaluate tumor-selective cellular uptake, ICP-MS analysis to quantify intracellular platinum accumulation in THLE-2 and HepG2 cells was performed after treatment with **DGA** NPs (5 μM) for 3, 6, 12 and 24 h. As shown in Fig. S27, the intracellular Pt content in HepG2 cells was approximately 3.63-fold higher than that in THLE-2 cells (783.0 ± 26.5 vs. 215.6 ± 12.3 ng Pt/10^6^ cells) at 24 h, confirming preferential uptake by HCC cells. These results demonstrate that **DGA** NPs exhibit superior tumor cell selectivity and lower toxicity to normal cells.

Given that drug resistance remains a major obstacle in chemotherapy, the potential of **DGA** NPs to overcome CDDP resistance was further investigated using a CDDP-resistant subline, Huh7/DDP. Cytotoxicity assays revealed that the IC_50_ of CDDP increased from 2.29 μM in Huh7 cells to 11.45 μM in Huh7/DDP cells, yielding a resistance index of 5.00 (Table 1). Strikingly, **DGA** NPs showed even higher activity against the resistant Huh7/DDP cells (IC_50_ = 1.18 μM) than against the sensitive parental line (IC_50_ = 1.54 μM). In contrast, the physical combinations CDDP + GA and CDDP + GA-NH_2_ showed markedly higher IC_50_ values of 11.48 μM and 9.87 μM, respectively, (Table S2). These results demonstrate that the nanoplatform uniquely overcomes CDDP resistance—a benefit not achieved by simple drug admixtures. Thus, conjugating GA to the Pt(IV) scaffold not only enhances the broad-spectrum anti-tumor efficacy of CDDP but also confers the ability to circumvent CDDP resistance, highlighting the therapeutic potential of this prodrug nanoplatform.

### 2.4 **DGA** NPs-Induced cell apoptosis

**Fig. 2:**
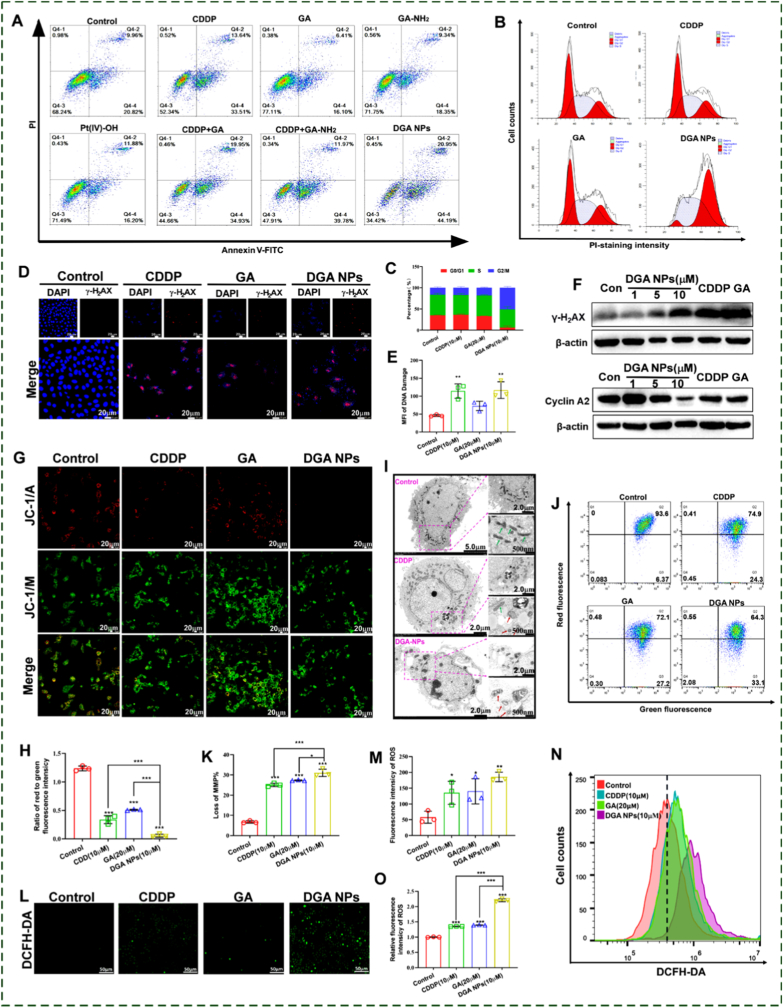
**Cytotoxic activity of DGA NPs in HepG2 cells**. (A) Apoptosis analysis in HepG2 cells treated with CDDP (5 μM), GA (10 μM), GA-NH_2_ (10 μM), Pt(IV)-OH (5 μM), CDDP + GA (5 μM and 10 μM), CDDP + GA-NH_2_ (5 μM and 10 μM) and **DGA** NPs (5 μM) for 48 h. **(B, C)** Cell cycle analysis in HepG2 cells after 48 h treatment (CDDP 10 μM, GA 20 μM, **DGA** NPs 10 μM). **(D, E)** γ-H_2_AX immunofluorescence after 48 h treatment (CDDP 10 μM, GA 20 μM, **DGA** NPs 10 μM) and quantitative analysis. **(F)** Western blot of Cyclin A2 and γ-H_2_AX. **(G, H)** Mitochondrial membrane potential (ΔΨm) by confocal microscopy and quantitative ΔΨm analysis. **(I)** TEM images of mitochondrial ultrastructure (red arrows: abnormalities). **(J, K)** ΔΨm quantified analysis by flow cytometry and quantitative ΔΨm analysis. **(L, M)** Intracellular ROS and quantitative ROS levels (confocal, 48 h). **(N, O)** Flow cytometry of intracellular ROS and quantitative ROS levels. Data are presented as mean ± SD (n = 3 biologically independent experiments). ∗p < 0.05, ∗∗p < 0.01, ∗∗∗p < 0.001 by ANOVA.

To assess the pro-apoptotic effects, HepG2 cells were treated for 48 h with various agents, including CDDP, GA, GA-NH_2_, Pt(IV)-OH, CDDP + GA, CDDP + GA-NH_2_ and **DGA** NPs, and then analyzed by flow cytometry using Annexin V-FITC/PI dual staining. As shown in Fig. 2A, none of GA, GA-NH_2_, or Pt(IV)-OH induced marked apoptosis relative to the control group. The physical combinations (CDDP + GA and CDDP + GA-NH_2_) induced comparable moderate apoptotic rates of 55.34% and 52.09%, respectively, which were slightly higher than that observed with CDDP (47.15%) alone. Notably, treatment with **DGA** NPs resulted in a substantially elevated apoptosis rate of 65.14%, which was significantly greater than those achieved with either physical combinations, CDDP monotherapy or Pt(IV)-OH (28.51%). This superior pro-apoptotic effect was also evident in cisplatin-resistant Huh7/DDP cells: **DGA** NPs induced significantly higher apoptosis (∼41.3%) than CDDP (∼17.9%) or Pt(IV)-OH (∼11.8%) (Fig. S28). Collectively, the enhanced efficacy demonstrates that the potent pro-apoptotic activity of **DGA** NPs arose not merely from the released GA moiety and CDDP, but from the integrated nanoplatform design, which maximizes the synergistic effect through targeted co-delivery, synchronized intracellular release, and improved cellular uptake.

### 2.5 DNA damage and cell cycle arrest

Following the establishment of the pro-apoptotic effects of **DGA** NPs, the underlying mechanism was investigated [23,24]. Flow cytometric analysis demonstrated that **DGA** NPs predominantly induced cell cycle arrest at the G2/M phase. The extent of this arrest was 3.0-fold and 2.8-fold greater than that caused by CDDP and GA, respectively (Fig. 2B and C). Mechanistically, this arrest was consistent with the dose-dependent downregulation of Cyclin A2, a key regulator of G2/M transition (Fig. 2F and Fig. S29). Given that G2/M arrest often arises from DNA damage, a significant increase in γ-H2AX (a marker for double-strand breaks) foci was observed via immunofluorescence in cells treated with CDDP or **DGA** NPs for 24 h, compared to control cells (Fig. 2D and E). This was further corroborated by Western blot analysis, which confirmed that **DGA** NPs dose-dependently elevated γ-H2AX protein levels in HepG2 cells (Fig. 2F and Fig. S29). Together, these results outline a clear mechanism whereby **DGA** NPs induce DNA damage, activate the G2/M checkpoint, and consequently trigger apoptosis.

### 2.6 ROS production and mitochondrial dysfunction

GA has been reported to function as an effective mitochondria-targeting ligand [[25], [26], [27]]. Mechanistically, GA interacts with mitochondrial respiratory chain complex I, modulating mitochondrial permeability transition pore (MPTP) opening and affecting reactive oxygen species (ROS) production. This process enhances mitochondrial membrane permeability, facilitating the accumulation of GA conjugates within the mitochondrial matrix. Given the mitochondrial targeting capability of GA, the ability of **DGA** NPs to induce mitochondrial dysfunction was subsequently evaluated. Fluorescence microscopy revealed a marked reduction in the ratio of JC-1 aggregates (red) to monomers (green) in **DGA** NPs-treated cells compared to controls, which was also lower than that in the CDDP or GA groups (Fig. 2G). Quantitative analysis confirmed that the red/green fluorescence ratio in the **DGA** NPs group reduced to merely 4.57% of that in the control, 16.76% of that in the CDDP group, and 11.06% of that in the GA group (Fig. 2H). TEM further corroborated severe mitochondrial damage in **DGA** NPs-treated cells, characterized by condensed matrix, loss of cristae, and increased membrane density (Fig. 2I, red arrows). Flow cytometry analysis was consistent with these findings, showing that while 93.6% of control cells maintained a high ΔΨm, drug-treated groups exhibited a pronounced shift to the depolarized state (Q3). The proportions of depolarized cells were 25.10%, 27.33%, and 31.03% in cells treated with CDDP (10 μM), GA (20 μM), and **DGA** NPs (10 μM), respectively (Fig. 2J and K). Obviously, **DGA** NPs disrupt mitochondrial function more effectively than CDDP or GA alone, leading to compromised cellular energy metabolism and viability.

Due to ROS production is a key mediator of mitochondrial dysfunction, intracellular ROS levels were evaluated using the DCFH-DA probe following 24 h of treatment [28]. Fluorescence microscopy observations showed that **DGA** NPs (10 μM) induced the most intense DCF signal, indicating the highest ROS levels in HepG2 cells, compared to CDDP (10 μM) or GA (20 μM) alone (Fig. 2L and M). Flow cytometry analysis further confirmed that exposure to all three treatments significantly elevated ROS levels relative to the control (Fig. 2N). Notably, the relative ROS fluorescence intensity in the **DGA** NPs-treated group was 2.21-fold higher than in the control, and 1.64-fold and 1.59-fold greater than the CDDP- and GA-treated groups, respectively (Fig. 2O). These processes operate in a mutually reinforcing cycle: ROS promote ΔΨm depolarization, which in turn amplifies ROS generation, thereby driving irreversible apoptosis.

### 2.7 Inhibition of HepG2 cell colony formation and migration

**Fig. 3:**
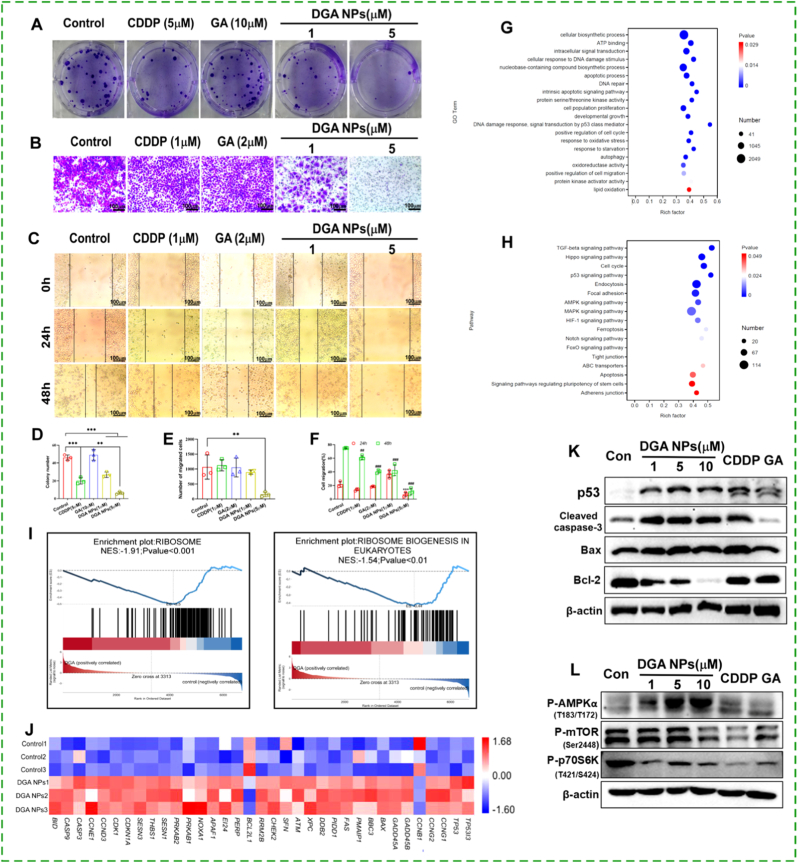
**DGA NPs induce apoptosis through coordinated activation of the p53 and AMPK/mTOR pathways**. (A) Colony formation in HepG2 cells treated with CDDP (5 μM), GA (10 μM), or **DGA** NPs (1, 5 μM) for 24 h. **(B)** Transwell migration and **(C)** wound healing assay in HepG2 cells treated with CDDP (1 μM), GA (2 μM), or **DGA** NPs (1, 5 μM) for 24 h or 48 h. **(D**–**F)** Quantitative results of colony formation, Transwell migration, and wound healing assays. **(G)** GO and **(H)** KEGG enrichment analysis of DEGs after **DGA** NPs (5 μM, 48 h) treatment. **(I)** GSEA of the ribosome pathway. **(J)** Heatmap of key genes in the p53 pathway. **(K)** WB analysis of p53 and apoptosis-related proteins. **(L)** Western blot analysis of the AMPK/mTOR/p70S6K pathway. Data are mean ± SD (n = 3 biologically independent experiments). ∗p < 0.05, ∗∗p < 0.01, ∗∗∗p < 0.001 by ANOVA.

To evaluate the anti-proliferative effect of **DGA** NPs, a colony formation assay was performed. Both CDDP and **DGA** NPs significantly reduced the colony-forming capacity of HepG2 cells, whereas GA alone showed minimal effect (Fig. 3A and D). Notably, at 5 μM, **DGA** NPs exhibited greater potency than either 5 μM CDDP or 10 μM GA. Furthermore, cell migration plays a pivotal role in the regulation of tumor metastasis. To evaluate the potential anti-metastatic effect of **DGA** NPs, its influence on the motility of HepG2 cells was further investigated using wound healing and transwell assays. **DGA** NPs (1 and 5 μM) suppressed HepG2 cell migration in a concentration-dependent manner, with effects substantially stronger than those of CDDP (1 μM) or GA (2 μM) at equivalent concentrations (Fig. 3B and E). For the wound healing assay, cells were pretreated, scratched, and monitored for 48 h. At 24 h, only 5 μM **DGA** NPs significantly inhibited wound closure; however, by 48 h, all treatment groups showed reduced migration compared to the control (Fig. 3C). Quantification revealed that the migration rates in **DGA** NPs-treated groups (42.46% and 11.33% for 1 and 5 μM, respectively) were markedly lower than those in the control (74.96%) and CDDP-treated (61.12%) groups at 48 h (Fig. 3F). Undoubtedly, these results demonstrate that **DGA** NPs not only exhibits enhanced cytotoxicity but also more effectively attenuates the migratory capacity of cancer cells compared to the individual components, CDDP and GA.

### 2.8 Apoptosis induction mechanism

To explore the mechanism of **DGA** NPs-induced cell death, RNA-seq was performed on HepG2 cells treated with 5 μM **DGA** NPs for 48 h. High-quality and reproducible data (average recovery rate >94%, inter-group correlation >0.85; Table S3; Fig. S30) enabled reliable downstream analysis. In contrast to the control, **DGA** NPs treatment yielded a considerable number of differentially expressed genes (DEGs) (defined by |fold change| ≥ 2 and p < 0.05) (Fig. S31). Gene Ontology (GO) enrichment analysis of these DEGs revealed significant enrichment in biological processes related to cellular biosynthesis, DNA damage response, apoptosis, p53 signaling pathway, cell cycle regulation, oxidative stress response, and ATP binding (Fig. 3G). Kyoto Encyclopedia of Genes and Genomes (KEGG) enrichment analysis indicated that **DGA** NPs treatment primarily affected key pathways including apoptosis, ferroptosis, p53 signaling pathway, and AMPK signaling pathway (Fig. 3H). Gene set enrichment analysis (GSEA) highlighted a pronounced downregulation of ribosome-related pathways, suggesting a global suppression of protein synthesis (Fig. 3I). This repression represents a classic stress adaption to conserve energy, which is typically regulated through inhibition of the AMPK/mTORC1 axis and activation of the p53 pathway [29,30]. Consistent with this, p53-related genes were markedly altered upon **DGA** NPs treatment (Fig. 3J).

To validate the transcriptomic findings at the mRNA level, qPCR analysis was performed for key p53 pathway-related genes (Fig. S32). **DGA** NPs treatment significantly increased the mRNA levels of p53 and Bax, while decreasing Bcl-2 expression compared with control, consistent with the RNA-seq data. Western blot analysis further confirmed these transcriptional changes at protein level. **DGA** NPs significantly increased the pro-apoptotic Bax/Bcl-2 ratio. This pro-apoptotic shift was further accompanied by increased levels of cleaved caspase-3, p53, and phosphorylated AMPK (Fig. 3K and L; Figs. S33 and S34). Furthermore, **DGA** NPs markedly suppressed phosphorylation of both mTOR and p70S6K, indicating that AMPK activation by DGA NPs transduces energy stress into apoptotic signaling via inhibition of the mTOR-p70S6K axis [31].

Collectively, these results demonstrate that **DGA** NPs activate two core apoptotic regulators—p53 and AMPK. p53 directly transactivates pro-apoptotic genes and modulates Bcl-2 family proteins to initiate cell death [32,33], while AMPK integrates metabolic stress signals to critically influence cell fate [34]. Notably, these pathways engage in synergistic crosstalk: AMPK phosphorylates and stabilizes p53, while p53 transcriptionally enhances AMPK activity, forming a positive feedback loop that robustly amplifies apoptotic signaling [35]. Thus, **DGA** NPs effectively harness this synergistic interplay between p53 and AMPK to induce apoptotic cell death in HepG2 cells.

### 2.9 Ferroptosis induction mechanism

**Fig. 4:**
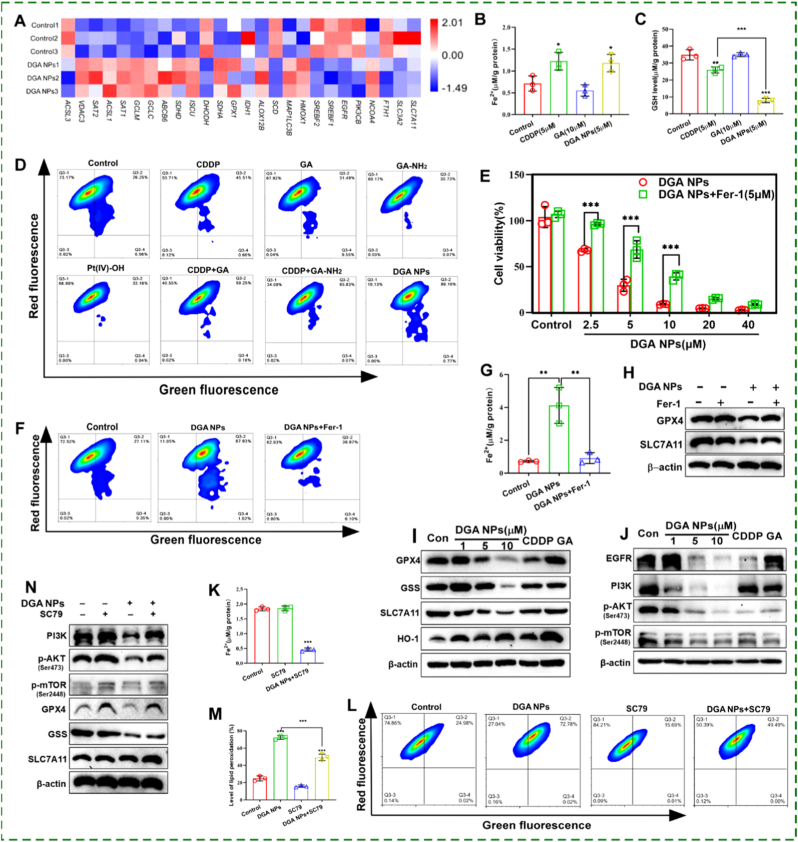
**DGA NPs induce ferroptosis via inhibition of the PI3K/AKT/mTOR pathway. (A)** Heatmap of key ferroptosis-related genes. **(B)** Intracellular Fe^2+^ and **(C)** glutathione (GSH) levels in HepG2 cells treated for 48 h with CDDP (5 μM), GA (10 μM), or **DGA** NPs (5 μM). **(D)** Flow-cytometric analysis of lipid peroxidation in HepG2 cells treated with CDDP (5 μM), GA (10 μM), GA-NH_2_ (10 μM), Pt(IV)-OH (5 μM), CDDP + GA(5 μM and 10 μM), CDDP + GA-NH_2_ (5 μM and 10 μM) and **DGA** NPs (5 μM) for 48 h. **(E)** Cell viability in HepG2 cells following 96-h treatment with varying concentrations of **DGA** NPs and Fer-1 (5 μM). **(F)** Flow cytometric analysis of lipid peroxidation in HepG2 cells treated with **DGA** NPs (5 μM) and Fer-1(5 μM) for 48 h. **(G)** Intracellular Fe^2+^ content in HepG2 cells treated with **DGA** NPs (5 μM) and Fer-1(5 μM) for 48 h. **(H)** Western blot analysis of GPX4 and SLC7A11 in HepG2 cells treated with **DGA** NPs (5 μM) and Fer-1(5 μM) for 48 h. **(I, J)** Western blot analysis of ferroptosis related proteins and the PI3K/AKT/mTOR pathway in HepG2 cells treated with **DGA** NPs for 48 h. (**K**) Intracellular Fe^2+^ levels in HepG2 cells after 48 h DGA NPs and SC79 co treatment. **(L, M)** Flow cytometric analysis of lipid peroxidation after 48 h **DGA** and SC79 co treatment. **(N)** Western blot of ferroptosis related proteins and the PI3K/AKT/mTOR pathway after 48 h **DGA** and SC79 co treatment. Data are mean ± SD (n = 3 biologically independent experiments). ∗p < 0.05, ∗∗p < 0.01, ∗∗∗p < 0.001 by one-way ANOVA.

The hallmark features of ferroptosis include the accumulation of lethal lipid peroxides (LPO), dysregulated iron metabolism, and impaired antioxidant defense, primarily through the glutathione peroxidase 4 (GPX4) axis [[36], [37], [38]]. Transcriptomic data suggested that **DGA** NPs sensitize HepG2 cells to ferroptosis (Fig. 4A). Based on these findings, intracellular Fe^2+^ and GSH levels were measured. **DGA** NPs, CDDP, CDDP + GA and CDDP + GA-NH_2_ all significantly increased the intracellular Fe^2+^ concentration. Both **DGA** NPs and CDDP reduced intracellular GSH concentration, with **DGA** NPs causing a more pronounced decrease than CDDP (Fig. 4B and C; Fig. S35). LPO was monitored via BODIPY™ 581/591C11 staining. As shown in Fig. 4D, **DGA** NPs induced the highest proportion of oxidized cells (89.87%), which was markedly higher than that observed with CDDP alone (46.17%) and the control (26.81%). Notably, **DGA** NPs also significantly elevated LPO compared with the physical combinations CDDP + GA (59.43%) and CDDP + GA-NH_2_ (65.90%). In contrast, neither Pt(IV)-OH (33.20%), GA (32.04%) nor GA-NH_2_ (30.80%) alone promoted LPO and Fe^2+^ level compared to the control (26.81%) group in HepG2 cells (Fig. 4D and Fig. S36). More importantly, in cisplatin-resistant Huh7/DDP cells, only **DGA** NPs markedly increased Fe^2+^ levels (∼1.6-fold relative to control) and also showed a tendency to elevate LPO (Fig. S37). These results demonstrate that conjugation of GA endows **DGA** NPs with a more potent ferroptosis-inducing capability than CDDP or non-conjugated Pt(IV) prodrugs.

To confirm the involvement of ferroptosis, rescue experiments were conducted using the specific ferroptosis inhibitor Fer-1. First, the cytotoxicity of Fer-1 in HepG2 cells was evaluated using the CCK8 assay. The results showed that Fer-1 had no significant cytotoxicity at concentrations of 1, 2, 5, and 10 μM after 96 h of treatment (Fig. S38). Therefore, a concentration of 5 μM was chosen for the following rescue experiments. As shown in Fig. 4E, co-treatment with Fer-1 partially reversed **DGA** NPs-induced cell death. Moreover, Fer-1 significantly attenuated the accumulation of ferrous iron (Fe^2+^) and the elevation of LPO levels induced by **DGA** NPs treatment alone (Fig. 4F and G). Western blot analysis further revealed that co-treatment with Fer-1 upregulated the protein expression levels of GPX4 and SLC7A11 in **DGA** NPs-treated cells (Fig. 4H). These results indicate that **DGA** NPs induce ferroptosis more potently than CDDP, while GA alone fails to trigger this form of cell death.

To further investigate the underlying mechanism, the expression of key ferroptosis related proteins was examined by Western blot. **DGA** NPs treatment led to a more pronounced downregulation of glutathione synthetase (GSS), solute carrier family 7 member 11 (SLC7A11), and GPX4 compared to other treatments, suggesting a stronger capacity to induce ferroptosis than CDDP. Heme oxygenase-1 (HO-1) expression was elevated under all treatment conditions (Fig. 4I and Fig. S39). HO-1 upregulation may further promote lipid peroxidation through iron release, thereby accelerating ferroptosis.

The PI3K-AKT-mTOR pathway plays a central role in regulating cellular susceptibility to ferroptosis [39], primarily by modulating lipid metabolism, iron homeostasis, and antioxidant responses [40]. Western blot analysis revealed that **DGA** NPs treatment markedly downregulated the expression of PI3K and its upstream receptor EGFR, while also suppressing the phosphorylation of AKT and mTOR, compared to other groups (Fig. 4J and Fig. S40). To determine whether the PI3K-AKT-mTOR axis functionally contributes to **DGA** NPs-induced ferroptosis, a rescue experiment was conducted using the AKT agonist SC79. The results showed that co-treatment with SC79 prevented the **DGA** NPs-induced increase in intracellular ferrous iron levels (Fig. 4K). Furthermore, flow cytometric analysis revealed that SC79 significantly alleviated the **DGA** NPs-triggered increase in LPO (Fig. 4L and M). Western blot analysis confirmed that SC79 restored phosphorylation of AKT and mTOR and, notably, reversed the downregulation of key ferroptosis-related proteins induced by **DGA** NPs (Fig. 4N and Fig. S41). Collectively, these data demonstrate that the suppression of the PI3K-AKT-mTOR signaling pathway is a critical mechanism through which **DGA** NPs induces ferroptosis in HepG2 cells.

### 2.10 Nrf2-dependent enhancement of ferroptosis

**Fig. 5:**
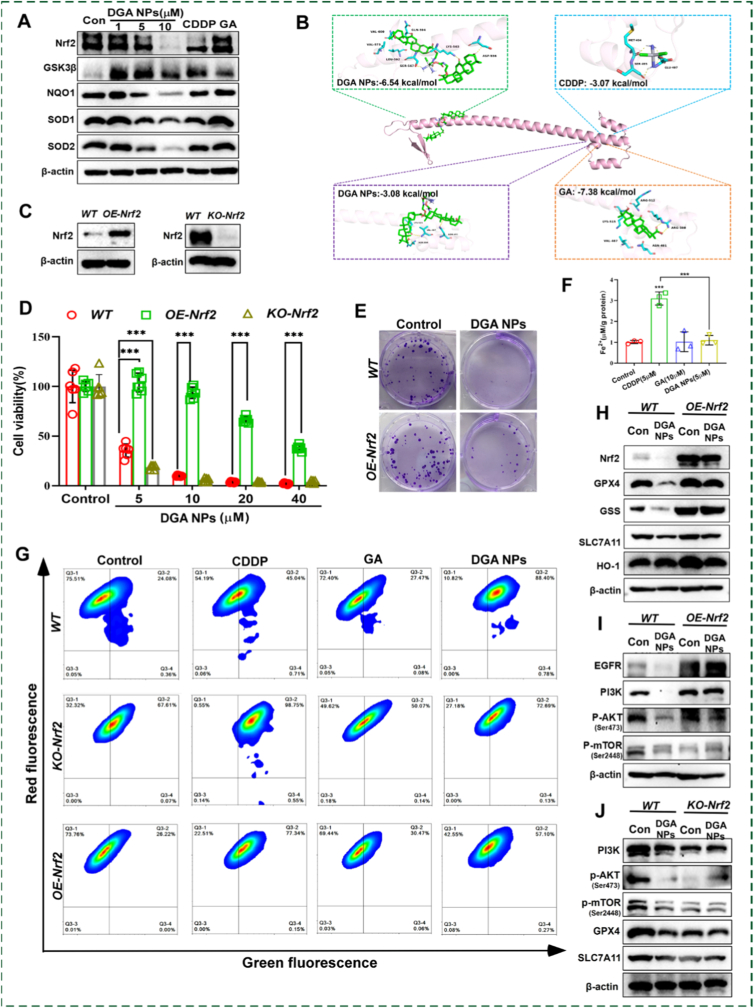
**Nrf2 Mediates DGA NPs-Triggered Ferroptosis in HepG2 Cells. (A)** Western blot assay of proteins related to the Nrf2 Pathway. **(B)** The molecular docking of different compounds and Nrf2 protein. **(C)** Western blot assay of Nrf2 protein expression in *OE-Nrf2* cell lines and *KO-Nrf2* cell lines. **(D)** Cell viability in *OE-Nrf2* cells and *KO-Nrf2* cells following 96-h treatment with varying concentrations of **DGA** NPs. **(E)** Representative pictures of the colony formation assay in *WT* and *OE-Nrf2* cells treated with **DGA** NPs (5 μM) for 24h. **(F)** Fe^2+^ content in *OE-Nrf2* cells following 48-h **DGA** NPs (5 μM) treatment. **(G)** Flow cytometric analysis of LPO on *WT* cells, *OE-Nrf2* cells and *KO-Nrf2* cells after 48h treatment with CDDP (5 μM), GA (10 μM), or **DGA** NPs (5 μM)**. (H, I)** Western blot detection of ferroptosis-related protein and PI3K/AKT/mTOR pathway expression in *OE-Nrf2* cells after 48-h **DGA** NPs (5 μM) treatment. **(J)** Western blot assay of ferroptosis related proteins and the PI3K/AKT/mTOR pathway in *WT* cells and *KO-Nrf2* cells following 48 h treatment. Data are mean ± SD (n = 3 biologically independent experiments). ∗p < 0.05, ∗∗p < 0.01, ∗∗∗p < 0.001 by ANOVA.

Given the central role of Nrf2–a key transcription factor in the cellular antioxidant response in regulating ferroptosis and cellular antioxidant defense, its involvement in **DGA** NPs-induced cell death was investigated [41,42]. WB analysis revealed that **DGA** NPs treatment significantly suppressed Nrf2 protein levels in HepG2 cells. Consistent with this downregulation, key antioxidant targets downstream of Nrf2, including superoxide dismutase 1, 2 (SOD1, SOD2) and dehydrogenase 1 (NQO1), exhibited dose-dependent reduction, while the upstream negative regulator GSK3β was upregulated (Fig. 5A and Fig. S42). These results demonstrate that **DGA** NPs attenuates the cellular antioxidant defense system via inhibition of the Nrf2 signaling axis.

To experimentally validate direct binding between GA and Nrf2, a Cellular Thermal Shift Assay (CETSA) was performed. GA treatment markedly increased the thermal stability of Nrf2, reflected by an elevated Tm value, indicating direct binding between GA and Nrf2 (Fig. S43). Molecular docking further elucidated the interaction: the intact **DGA** NPs conjugate displayed preferential binding to the leucine-zipper region of the Nrf2 Neh1 domain (residues 460–595) with a docking energy of −6.54 kcal/mol. In contrast, the released GA moiety bound with high affinity (GA: −7.38 kcal/mol, GA-NH_2_: −8.01 kcal/mol) to the adjacent basic region (residues 480–504), a site critical for DNA recognition and sMaf heterodimerization. Meanwhile, CDDP exhibited only weak binding (−3.07 kcal/mol) to this region (Fig. 5B and Fig. S44A). Western blot analysis confirmed that both GA and GA-NH_2_ suppressed Nrf2 protein levels and its downstream targets (SOD1, SOD2, NQO1), as well as the PI3K/AKT/mTOR signaling pathway, with GA-NH_2_ exhibiting slightly stronger potency (Fig. S44B and C). However, neither GA nor GA-NH_2_ promoted Fe^2+^ accumulation or LPO (Fig. 4D and S36A), indicating that Nrf2 inhibition alone is insufficient to trigger ferroptosis. In contrast, **DGA** NPs not only suppress Nrf2 but also deliver CDDP to elevate intracellular Fe^2+^ and deplete GSH, thereby synergistically inducing ferroptosis through a combination of antioxidant defense disruption and pro-oxidant iron accumulation.

Subsequently, the contribution of Nrf2 inhibition to **DGA** NPs-induced ferroptosis was validated in stable Nrf2-overexpressing (*OE-Nrf2)* and Nrf2-knockout (*KO-Nrf2)* HepG2 cells (Fig. 5C). *OE-Nrf2* cells displayed significantly higher cell viability, whereas *KO-Nrf2* cells exhibited significantly reduced cell viability compared to wild-type (*WT*) controls upon **DGA** NPs treatment in a dose-dependent manner, indicating that elevated Nrf2 levels mitigate the cytotoxicity of **DGA** NPs. This protective effect was further supported by colony formation assays (Fig. 5D and E).

To further investigate the role of Nrf2 in **DGA** NPs-induced ferroptosis, flow cytometry detection of LPO was performed in *WT, KO-Nrf2*, and *OE-Nrf2* cell lines treated with **DGA** NPs, CDDP, and GA at equivalent doses. As shown in Fig. 5G, *KO-Nrf2* cells displayed elevated basal LPO levels, likely due to the reduced basal antioxidant capacity resulting from Nrf2 deficiency. In *WT* cells, **DGA** NPs induced a 3.7-fold increase in LPO; in *OE-Nrf2* cells, the increase was only 2.1-fold, indicating that Nrf2 overexpression partially reverses **DGA** NPs induced ferroptosis. The partial reversal occurs because CDDP released from **DGA** NPs directly induces ferroptosis in an Nrf2-independent manner. Consistently, free CDDP treatment significantly increased LPO levels across all three cell lines. Importantly, in *KO-Nrf2* cells, **DGA** NPs did not further elevate lipid peroxidation beyond the already elevated basal level. Based on this observation, GSH levels in *WT* and *KO-Nrf2* cells were measured (Fig. S45). **DGA** NPs significantly depleted GSH level in *WT* cells. In contrast, basal GSH levels in *KO-Nrf2* cells were reduced by approximately 49.1% compared to *WT* cells, and GSH levels after **DGA** NPs treatment remained comparable to the basal level. This indicates that, due to severe basal GSH deficiency in *KO*-Nrf2 cells, **DGA** NPs cannot be effectively reduced to release CDDP. Therefore, although CDDP induces ferroptosis in an Nrf2 independent manner, **DGA** NPs are unable to further elevate LPO in *KO-Nrf2* cells. Furthermore, CDDP treatment significantly increased intracellular Fe^2+^ level in *OE-Nrf2* cells. In contrast, **DGA** NPs failed to induce notable changes Fe^2+^ levels (Fig. 5F). Collectively, these results confirm that Nrf2 functions as a critical suppressor of ferroptosis in this system.

Western blot analysis further revealed that Nrf2 overexpression significantly upregulated the protein levels of key ferroptosis-related factors, including GPX4, GSS, and HO-1 compared to *WT* cells, consistent with Nrf2-mediated transcriptional activation. Importantly, the downregulation of these proteins (GPX4, SLC7A11, and GSS) by **DGA** NPs was largely reversed in *OE-Nrf2* cells (Fig. 5H and Fig. S46). Given the established role of **DGA** NPs in suppressing the PI3K-AKT-mTOR pathway to promote ferroptosis, this signaling axis was examined in *OE-Nrf2* cells. Nrf2 overexpression restored the activity of the PI3K-AKT-mTOR pathway and its upstream regulator EGFR, which were suppressed by **DGA** NPs in *WT* cells (Fig. 5I). Conversely, the PI3K/AKT/mTOR pathway was inhibited in the *Nrf2-KO* cells, and the ferroptosis-related proteins GPX4 and SLC7A11 were downregulated (Fig. 5J). Moreover, **DGA** NPs treatment did not further enhance the inhibition of these proteins in *KO-Nrf2* cells, further supporting that **DGA** NPs and Nrf2 loss operate through the same pathway.

To explore the link between Nrf2 inhibition and suppression of the PI3K/AKT/mTOR pathway, qPCR analysis was performed. No significant changes were observed in the mRNA levels of *PIK3CA*, *PIK3R1* and *AKT1* (Fig. S47B). Additionally, PTEN protein levels remained unaltered (Fig. S47A), ruling out transcriptional regulation or PTEN-mediated effects. Notably, p-AMPK levels were significantly increased in *KO-Nrf2* cells and decreased in *OE-Nrf2* cells (Fig. S47C and D), suggesting that AMPK activation is a key event downstream of Nrf2 inhibition. Therefore, the downregulation of the PI3K/AKT/mTOR pathway following Nrf2 inhibition is likely an indirect, metabolically driven effect mediated by oxidative stress and AMPK activation. Taken together, **DGA** NPs exerts its anti-tumor effect by inhibiting Nrf2 to induce ferroptosis, a strategy that specifically overcomes cellular resistance mediated by both antioxidant adaptation and PI3K/AKT/mTOR survival signaling, resulting in synergistic lethality in HCC.

### 2.11 Pharmacokinetics and biodistribution of **DGA** NPs

Based on the unique structural and pharmacological features of **DGA** NPs, its pharmacokinetic (PK) behavior and biodistribution (BD) were evaluated in BALB/c mice bearing HepG2 xenografts following a single intravenous dose of either free CDDP or **DGA** NPs at an equivalent platinum dose of 3 mg-Pt/kg. Blood samples were collected at 15 min, 30 min, 1 h, 2 h, 4 h, 6 h, 8 h, and 24 h post-administration, and total platinum content was determined by ICP-MS. As shown in Fig. S48A and B, the plasma concentration-time profile revealed that **DGA** NPs achieved a 3.1-fold higher peak plasma concentration (C_max_: 2.53 ± 0.08 *vs.* 0.83 ± 0.05 μg/mL for CDDP). While free CDDP exhibited a rapid monophasic decline, **DGA** NPs displayed a markedly prolonged monophasic elimination phase, resulting in a substantially extended terminal half-life (t_1/2_: ∼18.5 vs. ∼2.0 h for CDDP). This sustained circulation is attributed to the nanoparticulate formulation, which minimizes renal clearance and reduces uptake by the reticuloendothelial system (RES). Consequently, the total systemic exposure (AUC_0-24h_) of **DGA** NPs was 11.9-fold higher than that of free CDDP (38.7 ± 1.8 *vs.* 3.25 ± 0.21 μg h/mL).

Biodistribution was assessed 24 h post-injection by measuring tissue platinum content via ICP-MS. Leveraging its prolonged circulation and EPR effect, **DGA** NPs achieved a 4.1-fold higher intra-tumoral platinum concentration than CDDP (3.37 ± 0.23 *vs.* 0.82 ± 0.08 μg/g). Notably, **DGA** NPs exhibited significantly reduced kidney retention, with a platinum concentration of 1.22 ± 0.05 μg/g, representing a 42% reduction compared to CDDP (2.10 ± 0.14 μg/g, Fig. S48C). Consequently, the Tumor/Kidney platinum ratio was 7.1-fold higher for **DGA** NPs (2.76) than for CDDP (0.39) (Fig. S48D). This favorable distribution profile indicates a substantially reduced risk of nephrotoxicity, a major dose-limiting factor for platinum-based therapies. Hepatic accumulation of **DGA** NPs was moderately elevated relative to CDDP (1.43 ± 0.08 *vs.* 1.05 ± 0.09 μg/g), consistent with the expected uptake of nanoparticles by the reticuloendothelial system. Collectively, these data demonstrate that **DGA** NPs, through the combined advantages of GA-mediated active targeting and the EPR effect, achieve sustained systemic exposure and preferential tumor accumulation while mitigating off-target renal exposure. This optimized PK/BD profile provides a robust pharmacokinetic foundation for the superior antitumor efficacy and improved safety profile of **DGA** NPs observed in vivo.

### 2.12 Antitumor efficacy in vivo

**Fig. 6:**
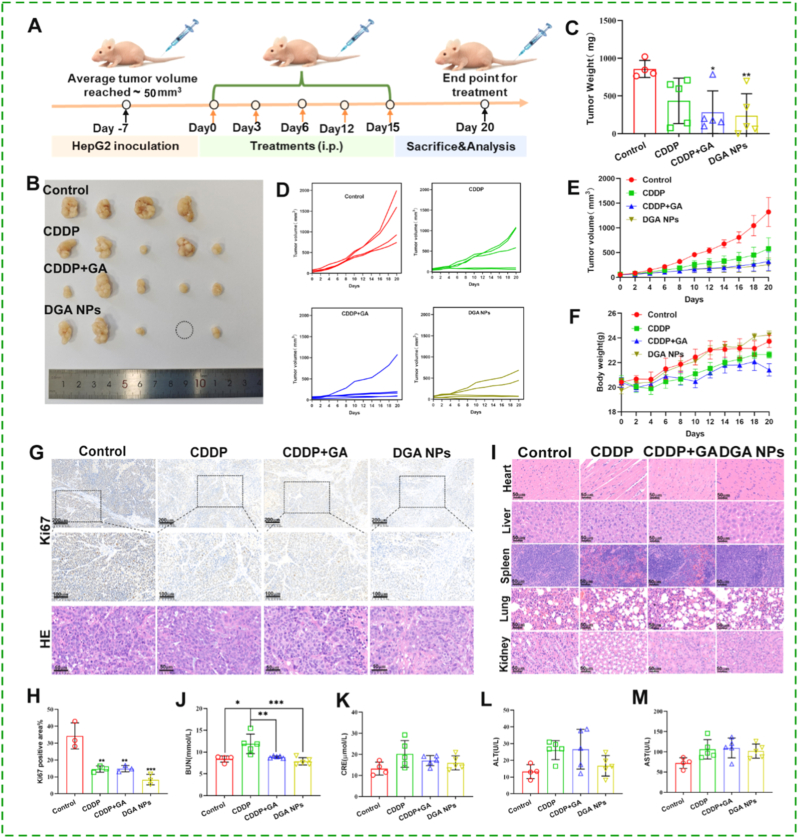
**Antitumor Effect of DGA NPs in BALB/c nude mice bearing HepG2 xenograft tumors. (A)** Treatment schedule. **(B)** Representative tumor images after saline (0.9%), CDDP (3 mg-Pt/kg), the combo of CDDP and GA (3 mg-Pt/kg + 8 mg/kg) and **DGA** NPs (3 mg-Pt/kg) treatment for 20 days. **(C)** Tumor weight**.** (**D, E**) tumor volume throughout the whole treatment period. **(F)** Monitoring of body weight. (**G**) Ki67 immunohistochemistry and Histological (HE) staining of tumor tissues. (**H**) Semi-quantitative analysis of Ki67 positivity rate on immunohistochemical. (**I**) HE staining of mouse major organs. (**J-M**) Serum levels of kidney function markers (BUN, CRE) and liver enzymes (ALT, AST). Data are mean ± SD (n = 4 mice for control group, n = 5 mice for other groups). ∗p < 0.05, ∗∗p < 0.01, ∗∗∗p < 0.001 by ANOVA.

The antitumor efficacy of **DGA** NPs was assessed in a HepG2 xenograft model established in BALB/c nude mice. Upon reaching a tumor volume of ∼100 mm^3^, the mice were randomly assigned into four groups: control (n = 4), CDDP (3 mg-Pt/kg, n = 5), CDDP + GA (3 mg-Pt/kg + 8 mg/kg, n = 5), and **DGA** NPs (3 mg-Pt/kg, n = 5). One mouse in the control group was excluded due to failure to develop tumors. Free GA (rather than GA-NH_2_ prodrug) was used for combination control, as both ultimately deliver the same active species. All treatments were administered intravenously every three days for a total of five doses (Fig. 6A). Tumor volume and body weight were recorded every two days to assess antitumor activity and systemic toxicity, respectively. **DGA** NPs treatment resulted in stronger tumor growth inhibition (TGI = 71.81%) compared with the CDDP (49.21%) and CDDP + GA (66.76%) groups (Fig. 6B–E). Consistently, the mean tumor weight at the endpoint was significantly lower in the **DGA** NPs group than in all other groups (Fig. 6C). Notably, this enhanced antitumor effect was accompanied by reduced systemic toxicity. Mice treated with CDDP alone or CDDP + GA showed significantly slower body weight gain, lethargy, and reduced activity, whereas animals receiving **DGA** NPs maintained stable body weight throughout the study (Fig. 6F), indicating an improved safety profile for the nano-prodrug formulation.

To further evaluate antitumor effects and potential organ toxicity, major organs and tumor tissues were obtained for histopathological and immunohistochemical analysis. Ki-67 staining revealed a significantly lower proliferation index in tumors from the **DGA** NPs group compared to all other groups (Fig. 6G and H). H&E staining showed no notable pathological changes in the spleen, lung, heart, or liver across treatment groups. However, the CDDP-alone group displayed severe renal tubular injury, characterized by disorganized architecture and cystic cavity formation, which was not observed in the **DGA** NPs or CDDP + GA groups (Fig. 6I). Serum biochemistry confirmed CDDP-induced nephrotoxicity, with elevated blood urea nitrogen (BUN) in the CDDP and CDDP + GA groups, whereas BUN levels in the **DGA** NPs group remained comparable to the control (Fig. 6J). Although mild increases in serum creatinine (CRE), aspartate aminotransferase (AST), and alanine aminotransferase (ALT) were observed across treatment groups, no statistically significant inter-group differences were detected, with the smallest increase seen in the **DGA** NPs group (Fig. 6K–M). Detailed body weight and serum biochemistry data are provided in Tables S5–S9. These results underscore the dual advantage of **DGA** NPs: enhanced antitumor efficacy coupled with significantly reduced systemic and renal toxicity compared to conventional CDDP therapy.

**Fig. 7:**
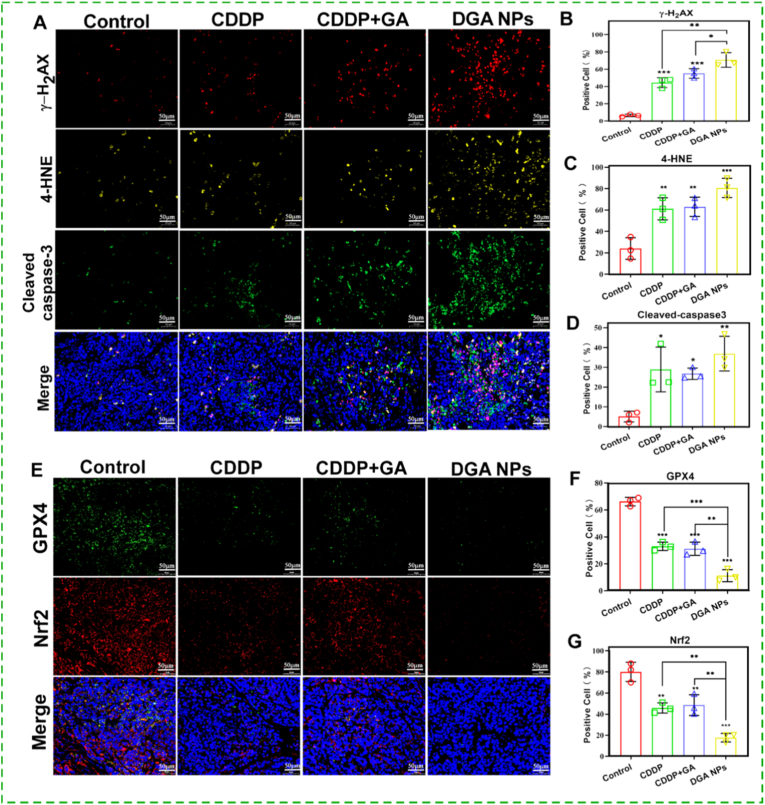
**Immunofluorescence analysis of tumor tissues. (A)** Representative images of triple staining for γ-H_2_AX, cleaved caspase-3 and 4-HNE. **(B**–**D)** Quantitative analysis of γ-H_2_AX-, cleaved caspase-3-, and 4-HNE-positive cells. **(E)** Representative images of double staining for GPX4 and Nrf2. **(F, G)** Quantitative analysis of GPX4-and Nrf2-positive cells. Data are presented as mean ± SD (n = 3 samples). ∗p < 0.05, ∗∗p < 0.01, ∗∗∗p < 0.001 (one-way ANOVA).

To investigate the apoptosis and ferroptosis mechanisms underlying the in vivo efficacy, tumor tissues were subjected to multiplex immunofluorescence staining. Compared with control group, all treatment groups showed increased positivity for γ-H_2_AX (DNA damage), cleaved caspase-3 (apoptosis), and 4-HNE (lipid peroxidation) (Fig. 7A).

In particular, the **DGA** NPs group exhibited the highest proportion of γ-H_2_AX-positive cells (Figs. 7B and 70.82%). The percentages of cleaved caspase-3 and 4-HNE-positive cells in the **DGA** NPs group were 36.95% and 80.71%, respectively, also exceeding those in other groups (Fig. 7C and D). Conversely, the expression of Nrf2 and GPX4 was markedly downregulated in all treatment groups, with the most pronounced reduction occurring in the **DGA** NPs group—only 17.76% for Nrf2 and 11.26% for GPX4 (Fig. 7E–G). These in vivo data demonstrate that **DGA** NPs exert potent antitumor activity through dual mechanisms: enhancing cytotoxic stress (DNA damage, apoptosis, and lipid peroxidation) while suppressing the Nrf2/GPX4 antioxidant defense axis, thereby sensitizing tumors to ferroptosis. This combination results in superior efficacy and a favorable safety profile compared with CDDP alone or CDDP combined with free GA.

## 3 Conclusions

In summary, we successfully designed and prepared **DGA** NPs, a self-assembled Pt(IV) prodrug nanoplatform that covalently conjugates CDDP with GA. The amphiphilic conjugate self-assembled into stable, mono-dispersed nanoparticles that demonstrated significantly enhanced cellular uptake and tumor-targeting capability. **DGA** NPs demonstrated potent antitumor activity through a dual-mechanism attack on HCC: it simultaneously induces apoptosis through DNA damage and AMPK/p53 activation, and drives ferroptosis via potent inhibition of the Nrf2 antioxidant pathway and its downstream PI3K/AKT/mTOR survival axis. This synergistic induction of two complementary cell death pathways underpinned the superior *in vitro* cytotoxicity of **DGA** NPs against a panel of cancer cells, including a CDDP-resistant model, and its potent anti-migratory effects. Strikingly, in vivo evaluations in a HepG2 xenograft model confirmed that **DGA** NPs achieved markedly enhanced antitumor efficacy compared to CDDP monotherapy or a CDDP/GA combination, while notably mitigating the systemic and nephrotoxic liabilities associated with conventional CDDP. Collectively, **DGA** NPs disrupt redox homeostasis and shift the balance from survival to death, co-activating apoptosis and ferroptosis. This multi-target strategy offers two key therapeutic advantages, including minimized systemic toxicity from tumor-selective activation and the overcoming of apoptosis resistance through Nrf2-inhibition-induced ferroptosis. As an integrated nanoplatform combining cytotoxic action, targeting capability, and multi-pathway modulation, **DGA** NPs represent a promising and innovative strategy for treating CDDP-resistant hepatocellular carcinoma.

## CRediT authorship contribution statement

**Xi Chen:** Conceptualization, Data curation, Formal analysis, Investigation, Methodology, Writing – original draft. **Siqian Cui:** Conceptualization, Data curation, Funding acquisition, Writing – review & editing. **Rongzhen Deng:** Formal analysis, Software, Visualization. **Hongwei Zhang:** Software, Visualization. **Zhengwen Zhang:** Software, Supervision, Validation. **Yiguo Zhang:** Conceptualization, Funding acquisition, Methodology, Project administration, Writing – review & editing.

## Declaration of competing interest

The authors declare that they have no known competing financial interests or personal relationships that could have appeared to influence the work reported in this paper.

## Data Availability

Data will be made available on request.
