# Supplementary material for: A highly biocompatible self-assembled Pt(IV) nanoplatform integrating Nrf2 inhibition for synergistic apoptosis and ferroptosis in hepatocellular carcinoma
Source: Mater Today Bio. 2026 Apr 20;38:103138. doi: 10.1016/j.mtbio.2026.103138 (PMC13134034; doi:10.1016/j.mtbio.2026.103138)
Supplement: Multimedia component 1 [file mmc1.docx]

**Supporting Information**

**A highly biocompatible self-assembled Pt(IV) nanoplatform integrating Nrf2 inhibition for synergistic apoptosis and ferroptosis in hepatocellular carcinoma**

Xi Chen ^a^, Siqian Cui ^b,c*^, Rongzhen Deng ^a^, Hongwei Zhang ^a^, Zhengwen Zhang ^d^, Yiguo Zhang ^a,e**^

***^a^ The Laboratory of Cell Biochemistry and Topogenetic Regulation, College of Bioengineering, Chongqing University, Chongqing, 400044, China***

***^b^*** ***Department of Oncology, Chongqing University Jiangjin Hospital, Chongqing, 402260, China***

***^c^ College of Chemistry and Chemical Engineering, Chongqing University, Chongqing, 400044, China***

*^d^ Laboratory of Neuroscience, Institute of Cognitive Neuroscience and School of Pharmacy, University College London, 29-39 Brunswick Square, London, WC1N 1AX, England, United Kingdom*

*^e^ Department of Laboratory Medicine, Chongqing Center for Clinical Laboratory, Chongqing Academy of Medical Sciences, Chongqing General Hospital, School of Medicine, Chongqing University, Chongqing, 401147, China*

*Corresponding author: Department of Oncology, Chongqing University Jiangjin Hospital, Chongqing, 402260, China

**Corresponding author: College of Bioengineering, Chongqing University, Chongqing, 400044, China

E-mail addresses: Dr. Cui (cuisq742278@163.com); Prof. Zhang ([yiguozhang@cqu.edu.cn](mailto:yiguozhang@cqu.edu.cn)).

**Material and methods**

**Chemicals and Materials**

All chemicals and reagents are purchased from MERYER Chemical Technology Co., Ltd (Shanghai, China) ，MREDA Technology Co., Ltd (Beijing, China) and HEOWNS Biochemical Technology Co., Ltd (Tianjin，China) without further purification, unless otherwise indicated.

Reagents and assay kits were obtained from commercial suppliers as specified. Cell Counting Kit-8 was sourced from BIOGROUND (Chongqing, China). The following were acquired from Beyotime (Shanghai, China): Ferrostatin-1 (Fer-1), Penicillin-Streptomycin Solution, Trypsin-EDTA Solution, Annexin V-FITC Apoptosis Detection Kit, ROS Assay Kit, Cell Cycle and Apoptosis Analysis Kit, GSH and GSSG Assay Kit, BODIPY™ 581/591 C11 Lipidation Peroxidation Assay Kit, Crystal Violet Staining Solution, MMP Assay Kit with JC-1, Cy3-labeled Goat Anti-Rabbit IgG (H + L), and HRP-labeled Goat Anti-Rabbit/Mouse IgG (H + L). The Live/Dead Cell Staining Kit was supplied by BestBio (Shanghai, China). DMEM and RPMI 1640 were purchased from Gibco (USA). FBS was procured from Procell (Wuhan, China). Ferrous Ion and Lipid Peroxide (LPO) Content Assay Kits were obtained from Macklin (Shanghai, China). Urea, Cr, AST, and ALT assay kits were purchased from Solarbio (Beijing, China). RNAiso Plus was purchased from Takara. SC79 (HY-18749) was purchased from MedChemExpress. The Nrf2 (ab62352) rabbit mAb was purchased from Abcam. The SLC7A11 (R382036) rabbit mAb, GPX4 (R381958) rabbit mAb, HO1 (R24541) rabbit mAb, SOD2 (306208) rabbit mAb, SOD1 (R25829) rabbit mAb, NQO1 (#R381695) rabbit mAb, p-AKT (#R381555) Rabbit mAb, p-mTOR (381557) Rabbit pAb, GSK3β (R22868) rabbit mAb, γ-H_2_AX (201082-7G9) mouse mAb, Bcl2 (#381702) rabbit pAb, Bax (#R380709) rabbit mAb were purchased from Zenbio. The p-AMPKα (CY5608) rabbit mAb, p-p70S6K (CY5261) rabbit mAb, Cleaved caspase-3 (CY5501) rabbit mAb, GSS(CY8598) rabbit mAb were purchased from Abways. The p53 mouse mAb was purchased from Huabio. The PI3K (A19742) rabbit mAb, EGFR (L858R) rabbit mAb, β-actin (AC004) mouse mAb were purchased from Abclonal.

**Cell culture**

The cell lines HepG2, MHCC-97H, Huh7, A549, HCT116, Hepa1-6, and the cisplatin-resistant subline Huh7/DDP were maintained in DMEM. CT26 cells were cultured in RPMI 1640. All media were supplemented with 10% FBS, 100 U/mL penicillin G, and 0.1 mg/mL streptomycin. Cells were incubated at 37 °C in a humidified atmosphere containing 5% CO₂.

**CCK8 assay**

Cells were seeded into 96-well plates at a density of 3,000 cells/well. Following a 24-hour attachment period, they were exposed to a range of compound concentrations. After 96 hours of treatment, cell viability was assessed by adding CCK-8 reagent (10% v/v in fresh medium) to each well, taking care to avoid bubble formation. The plates were then incubated for 1 hour before measuring the absorbance at 450 nm.

**Cell apoptosis**

Cells were seeded into 6-well plates at 3×10⁵ cells per well. Following seeding, the plates were incubated overnight. After cell adhesion, drug treatments were administered (cisplatin at 5 μM, GA at 10 μM, and DGA at 1, 5, and 10 μM, respectively). Following 48 hours of continued incubation, detection of apoptosis was carried out as recommended by the manufacturer.

**Live/dead assay**

Dishes were seeded with 3×10⁴ cells each and incubated overnight. Adherent cells were then treated with 5 μM **DGA NPs** for 96 hours prior to analysis. Following the staining procedure as described in the kit manual, observation and image acquisition were conducted using a laser scanning confocal microscope.

**Cell Cycle Arrest.**

Cells were seeded in 6-well plates at 3×10⁵ cells per well and cultured overnight. Following this, they were treated for 48 hours with CDDP (10 μM), GA (20 μM), or **DGA** NPs (10 μM). The culture medium and cell suspension were collected,and centrifuged, fixed in 70% pre-chilled ethanol for 30 min, stained as per the kit instructions, and analyzed on a flow cytometer.

**Colony forming assay**

Cells were plated in 6-well plates at 1000 cells per well and cultured overnight. CDDP (5 μM), GA (10 μM), and DGA (5 and 10 μM) was applied to treat the cells for 24 hours. Thereafter, the medium was replaced every 48 hours with freshly prepared DMEM complete medium to ensure nutrient supply and promote clone formation. After the culture period, colonies were fixed at 25 °C for 10–15 minutes and then stained with 1% crystal violet for 30 minutes prior to visualization.

**Wound healing assay**

Cells were plated at 5×10⁵ cells/well in 6-well plates and cultured to approximately 90% confluence. Following treatment with CDDP (1 μM), GA (2 μM), or DGA NPs (1, 2.5, 5 μM) for 48 h, a scratch was made using a sterile 10 μL tip. Then, the wells were rinsed and supplied with 2 mL of serum-free DMEM. The wound area was imaged at 0, 24, and 48 h under an inverted microscope to assess migration.

**Transwell assay**

Cells were plated overnight in 6‑well plates (3×10⁵ cells/well), then treated for 48 h with CDDP (1 μM), GA (2 μM), or **DGA** NPs (1 or 5 μM). After replacing the medium with serum‑free medium for 6 h, cells were trypsinized, counted, and seeded into the upper Transwell chamber (5×10⁴ cells/insert). The lower chamber was filled with 700 μL of medium supplemented with 10% FBS. Following 24 h of migration, cells on the membrane were fixed (20 min, 25 ℃), washed with PBS, and stained with crystal violet (30 min). Membranes were imaged under an inverted microscope.

**Mitochondrial membrane potential (MMP) assay**

To assess MMP by flow cytometry, adherent cells (3×10⁵/well in 6-well plates) were treated for 48 h with the indicated compounds (CDDP 10 μM, GA 20 μM, or **DGA** NPs 10 μM). After treatment, cells were collected, stained with JC-1 (37 °C, 20 min), washed, and resuspended in PBS for analysis. For parallel visualization by confocal microscopy, cells grown in confocal dishes underwent the same treatment protocol, followed by JC-1 staining (1 mL, 20 min, 37 °C). Following a buffer wash, samples were immersed in 2 mL of culture medium and immediately examined under a confocal microscope.

**Intracellular ROS assay**

For analysis of intracellular ROS by flow cytometry, adherent cells (3×10⁵/well in 6‑well plates) were treated for 48 h with the indicated compounds (CDDP 10 μM, GA 20 μM, or DGA NPs 10 μM), then harvested and centrifuged. The cell pellet was incubated with DCFH-DA solution (37 °C, 20 min, mixed every 3-5 min), washed twice with PBS, and resuspended for cytometry. For visualization by confocal microscopy, cells in confocal dishes received an 8‑h drug treatment, followed by incubation with diluted DCFH-DA (37 °C, 20 min). After three PBS washes, the samples were immediately examined under a confocal microscope.

**Immunostaining of** **γ-H_2_AX**

cells were first plated in confocal dishes (1×10⁵/dish) and exposed to CDDP (10 μM), GA (20 μM), or **DGA** NPs (10 μM) for 48 h. Subsequently, cells were fixed (4% PFA, 20 min, 25 ℃), permeabilized (0.5% Triton X-100, 15 min), and blocked with 5% BSA (60 min, 25 ℃). Immunostaining was performed by incubating with the primary antibody against γ-H2AX overnight at 4 °C, followed by a CY3-conjugated secondary antibody (1 h, 25 ℃, dark). Finally, after washing with PBST, nuclei were counterstained for imaging.

**Lipid peroxidation assay**

Cells were plated overnight in 6-well plates at a density of 3×10⁵ cells/well. They were subsequently treated for 48 h with CDDP (5 μM), GA (10 μM), or **DGA** NPs (5 μM). After digestion and collection, cells were incubated with BODIPY 581/591 C11 working solution (37 °C, 30 min). Finally, the samples were washed twice with PBS, resuspended, and subjected to flow cytometry.

**Intracellular GSH、LPO，Fe^2+^ level determination，**

Cells were plated in 6-well plates at 3×10^5^ cells per well and cultured overnight. CDDP (5 μM), GA (10 μM), and **DGA** (5μM) were applied to treat the cells for 48 hours. Following incubation, the cells were trypsinized and centrifuged. The resulting cell pellets were then analyzed using the following assay kits: GSH and GSSG Assay Kit (Beyotime Biotechnology, Cat# S0053)，Ferrous Ion Content Assay Kit (Solarbio, Cat# BC5410)，Lipid Peroxide (LPO) Content Assay Kit (Solarbio, Cat# BC5240). All assays were performed according to the manufacturers' instructions. Simultaneously, the protein concentration of the cell samples was quantified using the BCA method.

**Western blot assay**

After overnight culture in 6-well plates (3×10⁵ cells/well), cells were treated for 48 h with CDDP (5 μM), GA (10 μM), or **DGA** NPs (1, 5, 10 μM). Cells were then lysed on ice (20 min), and the protein supernatants were collected, mixed with loading buffer, and boiled for 15 min. Proteins were separated by 8-10% SDS-PAGE and transferred to a PVDF membrane. Subsequently, the membrane was blocked with 5% skim milk in PBST (1 h, 25 ℃), probed with primary antibody overnight (4 °C), and incubated with HRP-conjugated secondary antibody (1 h, 25 ℃). Following final washes, bands were detected using an ECL system and documented.

**Molecular docking**

The structural preprocessing of the receptor and ligand was performed using AutoDock Tools-1.5.7. Subsequent molecular docking studies were carried out with AutoDock, and the optimal conformation was ultimately selected based on its docking score and reasonable spatial orientation.

**Cellular Thermal Shift Assay (CETSA)**

HepG2 cells were grown to approximately 80% confluence in DMEM supplemented with 10% fetal bovine serum. Cells were treated with GA at a concentration of 20 μM or an equivalent volume of DMSO for 24 h. Following treatment, cells were harvested by trypsinization, washed twice with ice-cold phosphate-buffered saline (PBS), and resuspended in PBS containing protease inhibitor cocktail. The cell suspensions were aliquoted into PCR tubes (50 μL per tube) and subjected to a temperature gradient ranging from 37°C to 67°C at 5°C increments using a thermal cycler. Each temperature was maintained for 3 min, followed by a 3 min incubation at room temperature. The heated cell suspensions were then lysed by three freeze-thaw cycles .The lysates were clarified by centrifugation at 20,000 × g for 20 min at 4°C to separate soluble protein fractions from precipitated proteins. The supernatants (soluble fractions) were collected, mixed with 5× SDS loading buffer, and boiled at 95°C for 10 min.

**Quantitative Real-Time PCR (qPCR)**

Total RNA was extracted from HepG2 cells treated with the indicated compounds (CDDP 10 μM, GA 20 μM, or DGA NPs 10 μM) using BGMG Tissue/Cell RNA Kit according to the manufacturer’s instructions. RNA concentration and purity were assessed by measuring the absorbance at 260 nm and 280 nm using a NanoDrop spectrophotometer. Reverse transcription was performed using. PrimeScript RT Master Mix (Takara). The resulting cDNA was diluted [1:10] with nuclease-free water for further analysis. Quantitative real-time PCR was performed using SYBR Prime qPCR Set (BioGround) on a CFX96 Real-Time PCR System (Bio-Rad).Each reaction mixture contained a final volume of 10 μL. The thermal cycling conditions were as follows: initial denaturation at 95°C for 10 min, followed by 40 cycles of 95°C for 15 s and 60°C for 1 min. A melting curve analysis was performed for SYBR Green assays from 60°C to 95°Cwith increments of 0.5°C every 5 s to verify the specificity of the amplification and the absence of primer-dimer formation. The relative expression levels of target genes were calculated using the 2^(-ΔΔCt) method. The expression levels were normalized to the housekeeping gene GAPDH as an internal control. The primer sequences are listed in Table S4 and were synthesized by Beijing Tsingke Biotech Co., Ltd.

**RNA-seq**

HepG2 cells were seeded at 3×10^5^ per well in 6-well plates, with three replicates each for the negative control and DGA (5μM) treatment groups. After 48 hours, RNA was extracted with RNAiso Plus Reagent (Takara) and checked for quality (NanoDrop, Thermo Fisher). From 3 µg of total RNA, mRNA was isolated using poly‑T magnetic beads and fragmented under high‑temperature conditions. First‑ and second‑strand cDNA was synthesized with random primers/SuperScript II and DNA Polymerase I/RNase H, respectively. After end‑blunting, A‑tailing, and adapter ligation, libraries were size‑selected (400 - 500 bp) with AMPure XP beads, PCR‑amplified (15 cycles), and repurified. Quality control was conducted on an Agilent Bioanalyzer 2100, and sequencing was carried out on an Illumina NovaSeq 6000 at Shanghai Personal Biotechnology Co., Ltd.

**In vivo biodistribution experiment**

The pharmacokinetics and tissue distribution of **DGA** NPs and CDDP were assessed in HepG2 tumor-bearing mice following a single intravenous dose (3.0 mg Pt/kg, equimolar). Blood was collected at serial time points for plasma preparation. At 24 h post‑administration, mice were euthanized, and major organs and tumors were harvested. Platinum concentrations in all samples were determined by ICP‑MS.

**In vivo antitumor activity**

All animal experiments were approved by the Institutional Animal Care and Use Committee of Chongqing University (CQU-IACUC-RE-202510-002) and performed in compliance with relevant ethical guidelines. Twenty male BALB/c-nu mice (6–8 weeks old) were obtained from SPF (Beijing) Biotechnology Co., Ltd. After one week of acclimation, HepG2 cells (2 × 10⁶) were subcutaneously implanted into the right flank of each mouse. When tumor volumes reached ~ 50 mm³, the mice were stratified by tumor size and body weight into four groups (n = 5, except the control group which had n = 4 due to one mouse failing to form a tumor). Treatments were administered via tail vein injection every three days for five total doses, starting on day 0. Tumor dimensions and body weight were measured every two days; volume was calculated as (width² × length)/2. The study concluded on day 20. Following orbital blood collection and euthanasia, tumors and major organs (heart, liver, spleen, lungs, kidneys) were harvested and fixed in 4% paraformaldehyde. Serum was isolated from blood for biochemical analysis. Fixed tissues were processed for H&E staining, Ki67 immunohistochemistry (tumor), and multiplex immunofluorescence targeting Nrf2, GPX4, γ-H2AX, 4-HNE, and Cleaved Caspase-3.

**Experimental Section**

The reaction progress was monitored by thin-layer chromatography (TLC). The spectroscopic data of the compounds were obtained using the following instruments: i）FT-IR: Thermo Fisher iS5 spectrometer. ii）NMR: Bruker Avance spectrometer (¹H: 400/500 MHz; ¹³C: 100/125 MHz, ^195^Pt: 129 MHz), using DMSO‑*d*_6_ as the solvent. iii）High‑resolution mass spectrometric (HRMS) & LC‑MS: LCMS-8065XE instrument fitted with an ESI ion source and run at negative ion mode. iv) X‑ray photoelectron spectroscopy (XPS): Thermo Scientific K‑Alpha⁺ spectrometer with monochromatic Al Kα X‑ray source.

The morphology and structure of the nanoparticles were systematically characterized using SEM (Hitachi SU8010, Japan), TEM (JEOL JEM-2100F, Japan), and AFM (Bruker Multimode 8, USA). Hydrodynamic diameter and zeta potential were assessed using a Malvern Zetasizer Nano ZS (Malvern Panalytical, UK) via dynamic light scattering (DLS). The FT-IR、^1^H and ^13^C NMR、HRMS and LC-MS spectra of the synthesized compounds are displayed in Figures S1−S18.

**Scheme S1.** Synthesis route of **DGA**.

**Synthesis steps of intermediates 1-4 and target compound DGA**

**Synthesis of intermediate** **1：**Firstly, cisplatin (10.00 mmol, 3.00 g) was suspended in 20 mL ultrapure water, then 30% H_2_O_2_ (30 mL) was slowly added under stirring conditions. The reaction was conducted at 60 °C for 10 h in the dark and tracked by TLC. After completion, 50% of the solvent was removed by vacuum concentration. Crystallization of the remaining mixture at 5 °C, followed by filtration and sequential washing of the solid with ice water and ethanol, gave intermediate **3** as a yellow solid (2.28 mmol, 1.34 g). The structural characteristics data are as follows:

**1**: IR (KBr) 3459 (O-H), 3221 (N-H), 3141, 1577, 1441, 1374, 1075, 857, 575 (Pt-O), 539. In the Pt 4f XPS spectrum, the main peaks observed at 74.9 eV and 78.25 eV are assigned to the Pt 4f_7/2_ and Pt 4f_5/2_ spin-orbit doublet, respectively, corresponding to the Pt^4+^ oxidation state​.

**Synthesis of intermediate** **2:** Compound **1** (0.66 g, 2.00 mmol) in anhydrous DMF (5 mL) was charged with N, N′-disuccinimidyl carbonate (1.15 g, 4.5 mmol). The reaction was maintained at 70 °C for 8 h with TLC monitoring. After completion, the mixture was diluted with diethyl ether (20 mL) to induce precipitation. The collected solid was subjected to silica gel column chromatography (eluent: CH₂Cl₂/CH₃OH), affording compound **2** as a yellow solid (1.15 g, 1.87 mmol). The structural characteristics data are as follows:

**2**: ^1^H NMR (500 MHz, DMSO-*d*_6_) δ 6.61 (s, 6H), 2.74 (s, 8H). ^13^C NMR (125 MHz, DMSO-*d*_6_) δ 170.79, 154.75, 26.40. ^195^Pt NMR (129 MHz, DMSO-*d*_6_) δ 1293.34. HRMS (m/z) (ESI): calcd for C_10_H_14_Cl_2_N_4_O_10_Pt [M + H] ^+^, 613.9656; Found, 614.0870.

**Synthesis of intermediate** **3:** To a solution of 18β-GA (3.00 mmol, 1.41 g) in 10 mL dry DMF, NaHCO_3_ (4.5 mmol, 0.48 g) and 2-(boc-amino)ethyl bromide (6.00 mmol, 1.34 g) were added and then the mixture was stirred at 60 °C for 8 h, and the reaction process was monitored by TLC. The reaction mixture was concentrated under vacuum to remove DMF. The resulting crude product was then subjected to purification by silica gel column chromatography (CH₂Cl₂/CH₃OH), giving compound 3 as a white solid (2.28 mmol, 1.34 g). The structural characteristics data are as follows:

**3**: FT-IR (cm^-1^): 3435, 2957, 2867, 1727, 1657, 1451, 1389, 1328, 1249, 1216, 1155, 1077, 1039, 981, 875, 763, 661, 537. ^1^H NMR (400 MHz, DMSO-*d*_6_) δ 7.47 (s, 1H), 6.89 (s, 1H), 5.46 (s, 1H), 4.32 – 4.27 (m, 2H), 4.04 (t, *J* = 5.6 Hz, 2H), 3.17 (d, *J* = 5.2 Hz, 2H), 3.05 – 2.96 (m, 1H), 2.32 (s, 1H), 2.13 – 1.98 (m, 2H), 1.85-1.64 (m, 6H), 1.54-1.50 (m, 2H), 1.40-1.31 (m, 16H), 1.25 – 1.20 (m, 2H), 1.10 (s, 3H), 1.03 (d, *J* = 2.4 Hz, 6H), 0.98 – 0.92 (m, 2H), 0.91 (s, 3H), 0.75 (s, 3H), 0.69 (s, 3H). ^13^C NMR (100 MHz, DMSO) δ 199.46, 176.28, 169.67, 156.07, 127.99, 78.19, 77.06, 64.59, 63.16, 61.61, 54.56, 48.33, 45.32, 43.98, 43.33, 37.82, 37.12, 36.25, 32.59, 31.99, 31.24, 30.71, 28.66, 28.60, 28.18, 27.43, 26.51, 26.25, 23.48, 18.79, 17.62, 16.63, 16.46. HRMS (m/z) (ESI): calcd for C_37_H_60_NO_6_ [M + H] ^+^, 614.4421; Found, 614.4832.

**Synthesis of intermediate** **4**: To a solution of compound **3** (2.00 mmol, 1.18 g) in anhydrous CH₂Cl₂ (5 mL) was added TFA (2 mL). The reaction was stirred at 25 ℃ for 1 h (monitored by TLC), then quenched with saturated NaHCO₃ solution. After extraction with CH₂Cl₂ (2 × 100 mL), the organic phase was washed with brine, dried over Na₂SO₄, and concentrated in vacuo. The residue was subjected to silica gel column chromatography (eluent: CH₂Cl₂/CH₃OH), giving compound 4 as a white solid (1.82 mmol, 0.91 g). The structural characteristics data are as follows:

**4**: FT-IR (cm^-1^): 3392, 2970, 2870, 1722, 1660, 1529, 1458, 1388, 1251, 1163, 1087, 1039, 989, 871, 771, 665, 541. ^1^H NMR (500 MHz, DMSO-*d*_6_) δ 5.45 – 5.43 (m, 2H), 4.30 (s, 2H), 4.09 – 4.04 (m, 2H), 3.02 (dt, *J* = 10.0, 4.5 Hz, 2H), 2.89 (t, *J* = 6.0 Hz, 1H), 2.10-2.07 (m, 2H), 2.02 – 1.99 (m, 1H), 1.91 – 1.61 (m, 6H), 1.53 – 1.50 (m, 2H), 1.47 – 1.34 (m, 7H), 1.26 – 1.22 (m, 2H), 1.11 – 1.08 (m, 4H), 1.03 (s, 6H), 0.97 – 0.94 (m, 2H), 0.91 (s, 3H), 0.77 – 0.69 (m, 7H). ^13^C NMR (125 MHz, DMSO-*d*_6_) δ 199.39, 175.39, 170.17, 127.94, 77.07, 61.64, 54.57, 48.45, 45.35, 44.03, 43.37, 39.24, 38.97, 37.13, 32.60, 32.02, 28.70, 28.61, 28.12, 27.43, 26.53, 26.24, 23.46, 18.82, 17.62, 16.63, 16.45. HRMS (m/z) (ESI): calcd for C_32_H_51_NO_4_ [M + H] ^+^, 514.3896; Found, 514.3888.

**Synthesis of the targeted DGA**: To a solution of compound **2** (0.20 mmol, 0.12 g) in anhydrous DMF (2 mL) was charged compound **4** (0.42 mmol, 0.21 g). After stirring at 50 °C overnight (progress monitored by TLC), the mixture was concentrated under vacuum. Purification by silica gel column chromatography using V_CH₂Cl₂_/V_CH₃OH_ (4:1) as eluent gave the final product **DGA** conjugate as a white solid (0.08 mmol, 0.11 g). The structural characteristics data are as follows:

**DGA**: FT-IR (cm^-1^): 3459, 3402, 3251, 2939, 2865, 1729, 1651, 1504, 1455, 1389, 1332, 1257, 1217, 1143, 1087, 1032, 995, 880, 786, 672, 594, 504. ^1^H NMR (500 MHz, DMSO-*d*_6_) δ 6.72 – 6.64 (m, 6H), 5.42 (s, 2H), 4.30 (d, *J* = 5.0 Hz, 2H), 4.04 – 3.98 (m, 4H), 3.13 (q, *J* = 6.5 Hz, 4H), 3.02 (dt, *J* = 10.5, 4.5 Hz, 2H), 2.59 – 2.56 (m, 4H), 2.33 (s, 2H), 2.12 – 2.06 (m, 2H), 2.0 – 1.97 (m, 2H), 1.84 (d, *J* = 12.5 Hz, 2H), 1.76 – 1.70 (m, 4H), 1.67 – 1.62 (m, 2H), 1.53 – 1.51 (m, 4H), 1.45 – 1.31 (m, 14H), 1.26 – 1.23 (m, 4H), 1.17 – 1.11 (m, 14H), 1.04 – 1.03 (m, 10H), 0.97 – 0.94 (m, 4H), 0.91 (s, 6H), 0.75 (s, 6H), 0.69 (s, 6H). ^13^C NMR (125 MHz, DMSO-*d*_6_) δ 199.58, 176.22, 173.19, 169.87, 127.88, 77.06, 61.62, 54.57, 48.48, 45.34, 43.97, 43.37, 37.84, 37.14, 32.60, 32.01, 30.90, 30.75, 28.74, 28.60, 28.19, 27.44, 26.53, 26.25, 25.70, 23.45, 18.83, 17.62, 16.65, 16.45. ^195^Pt NMR (129 MHz, DMSO-*d*_6_) δ 1228.48. HRMS (m/z) (ESI): calcd for C_16_H_106_Cl_2_N_4_O_12_Pt [M – H]^–^, 1411.6832; Found, 1411.6770. Purity 95.79% (HPLC).


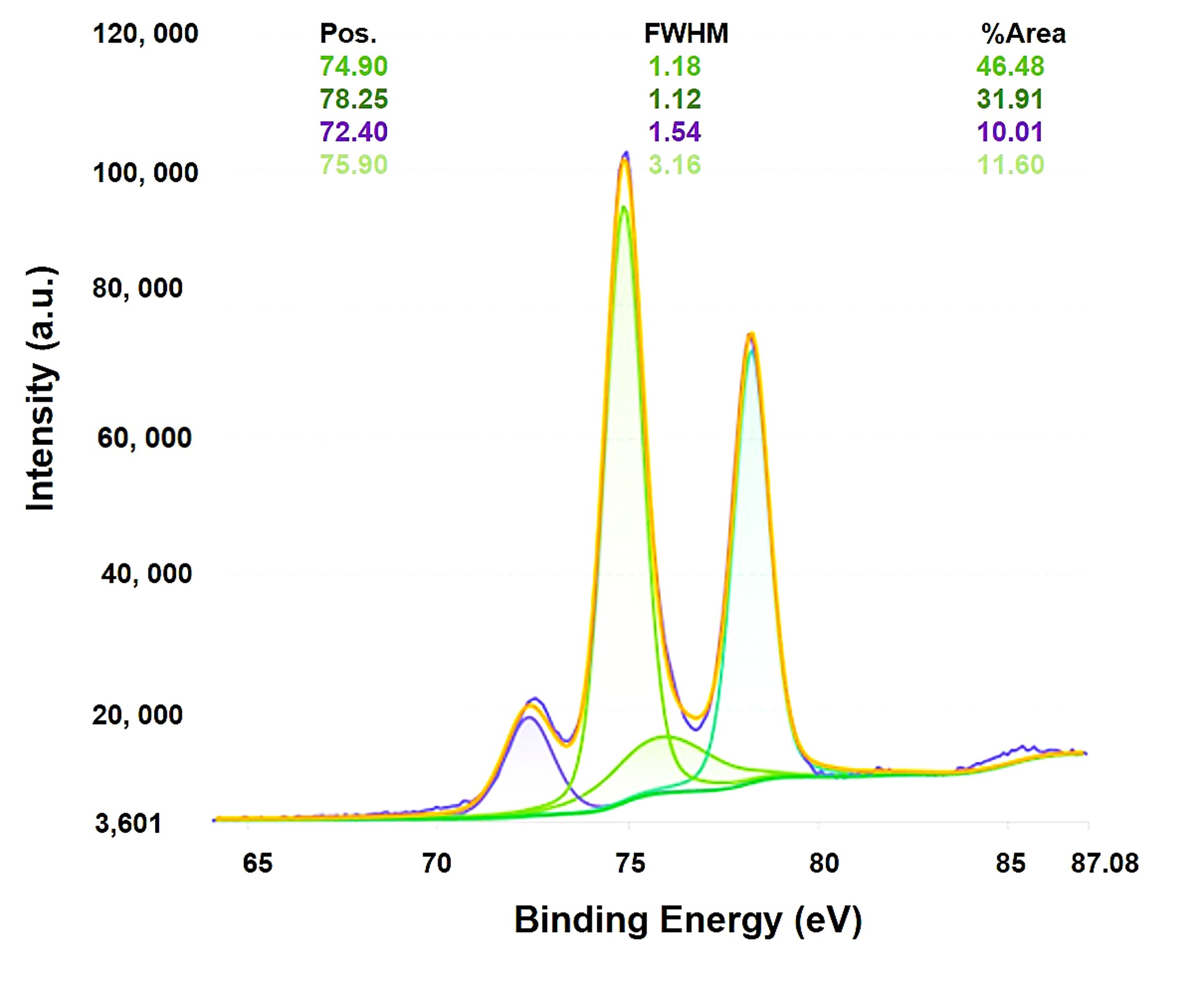
**Fig. S1** XPS spectra of intermediate **1**.


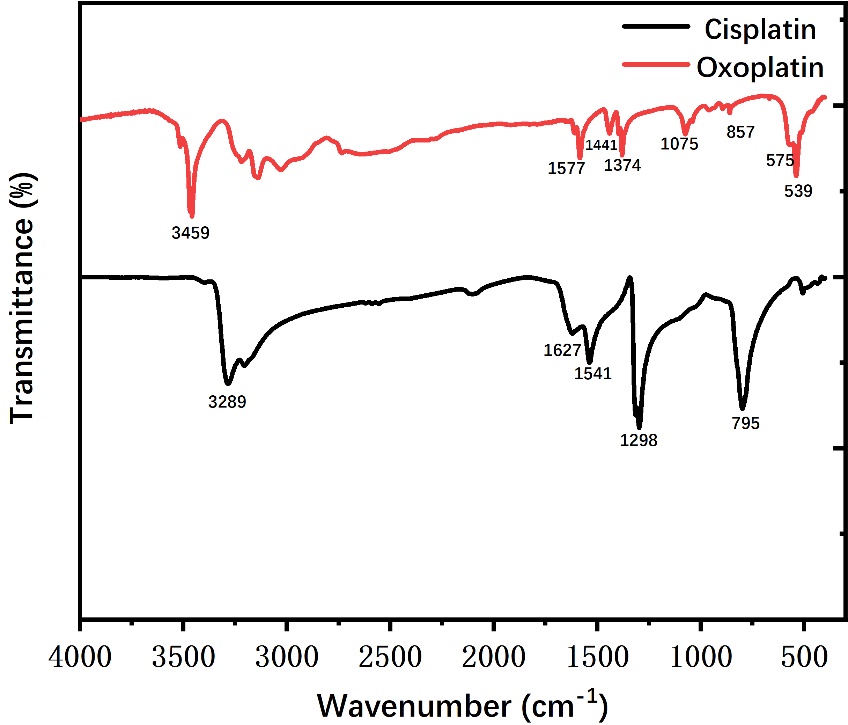


**Fig. S2**. FT-IR spectra of CDPP and Cis (IV)-2OH (intermediate **1**).


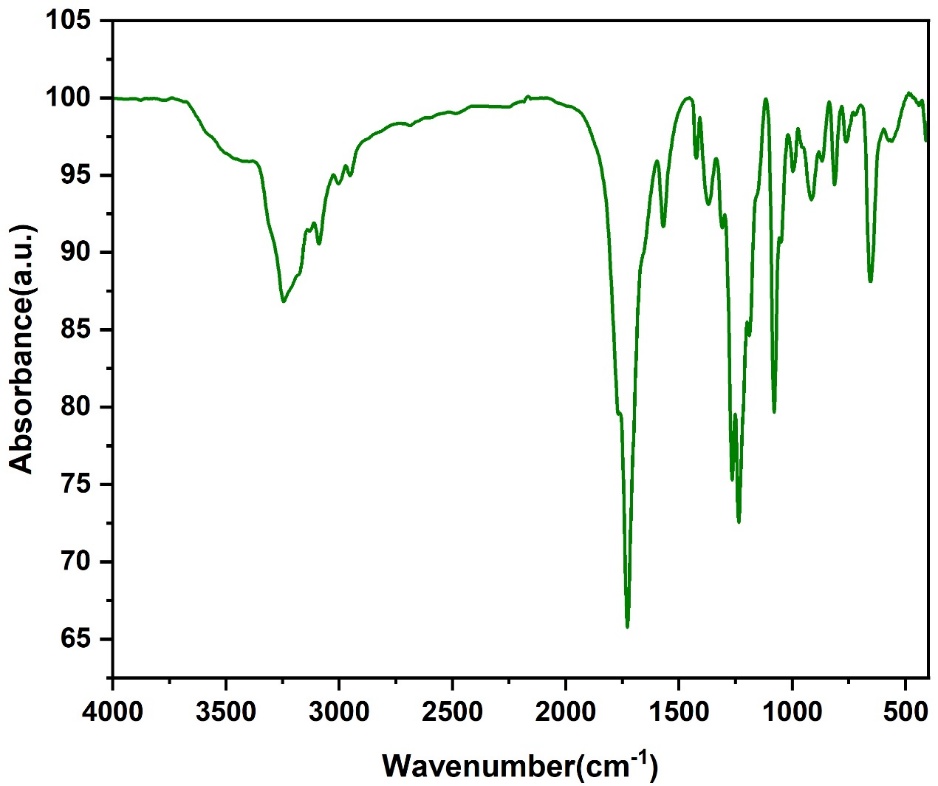


**Fig. S3**. FT-IR spectra of intermediate **2**.


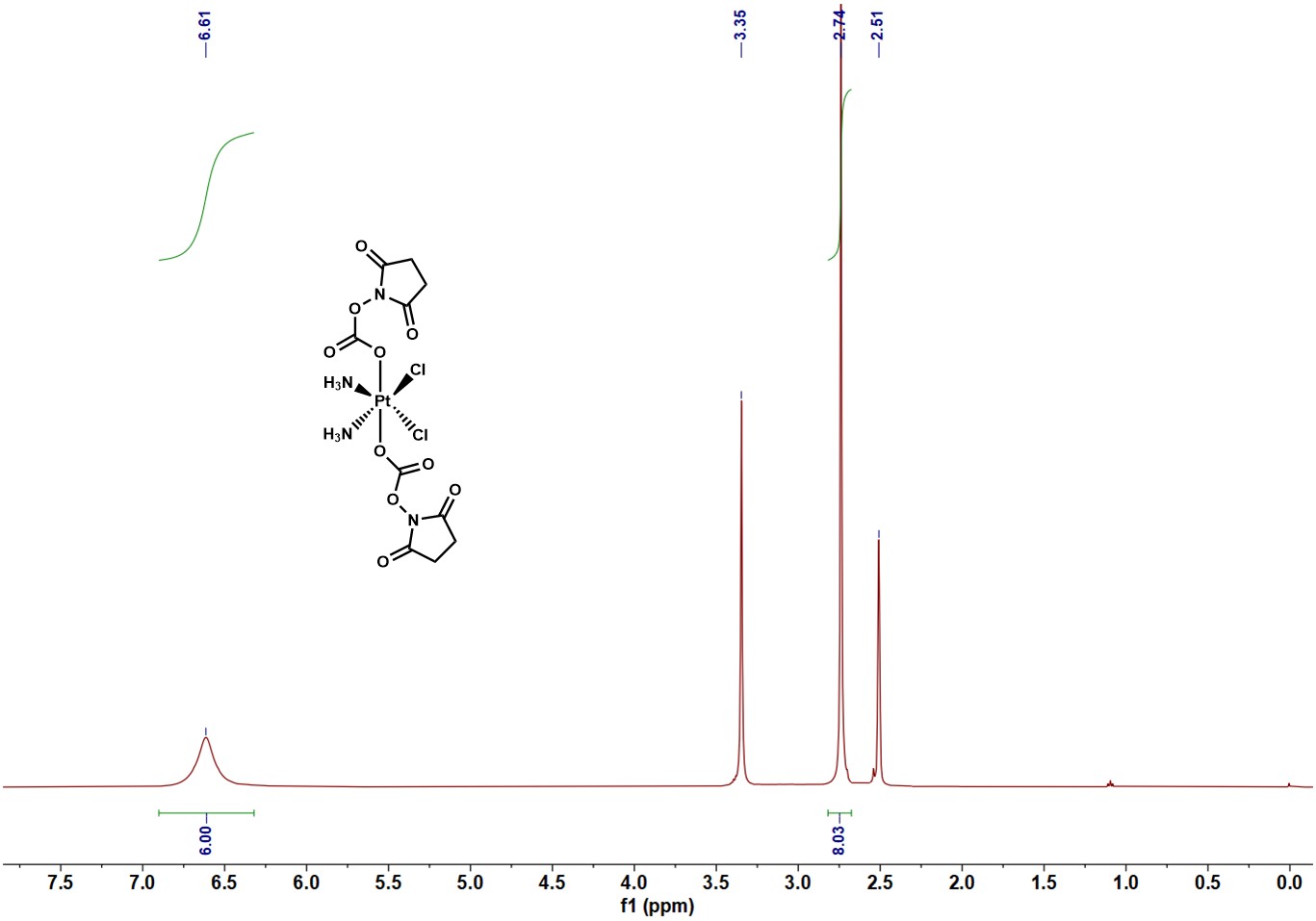
**Fig. S4**. ^1^H NMR spectra of intermediate **2**.


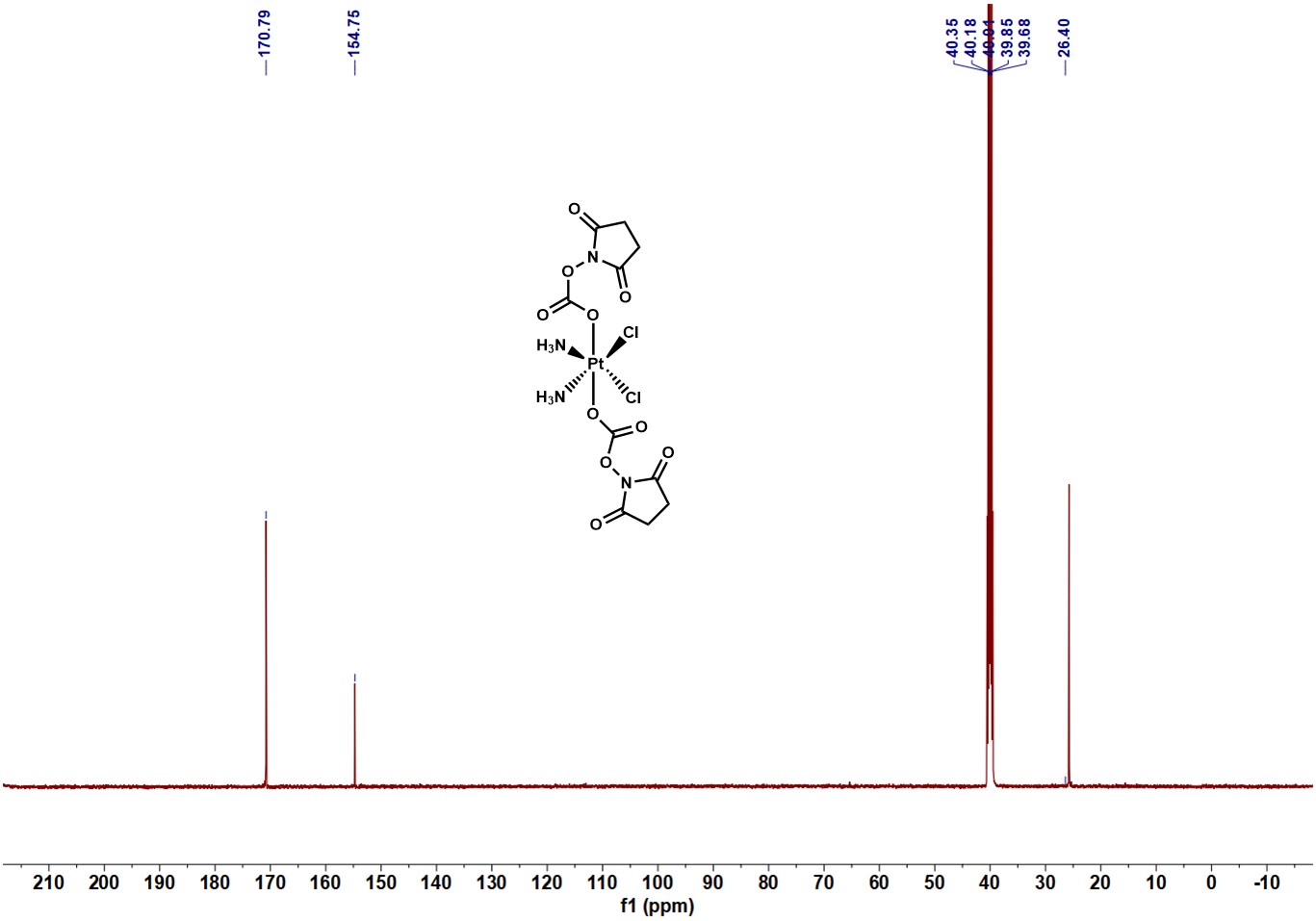


**Fig. S5**. ^13^C NMR spectra of intermediate **2**.


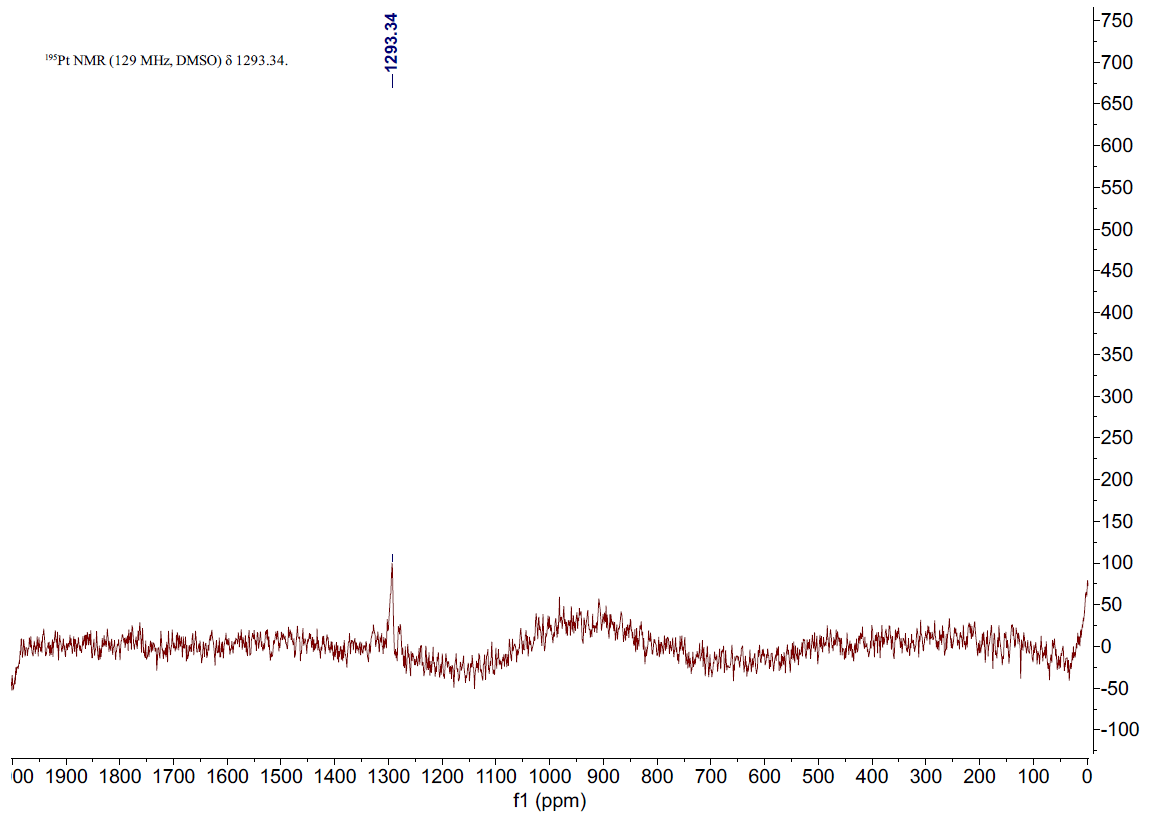
**Fig. S6**. ^195^Pt NMR spectra of intermediate **2**.


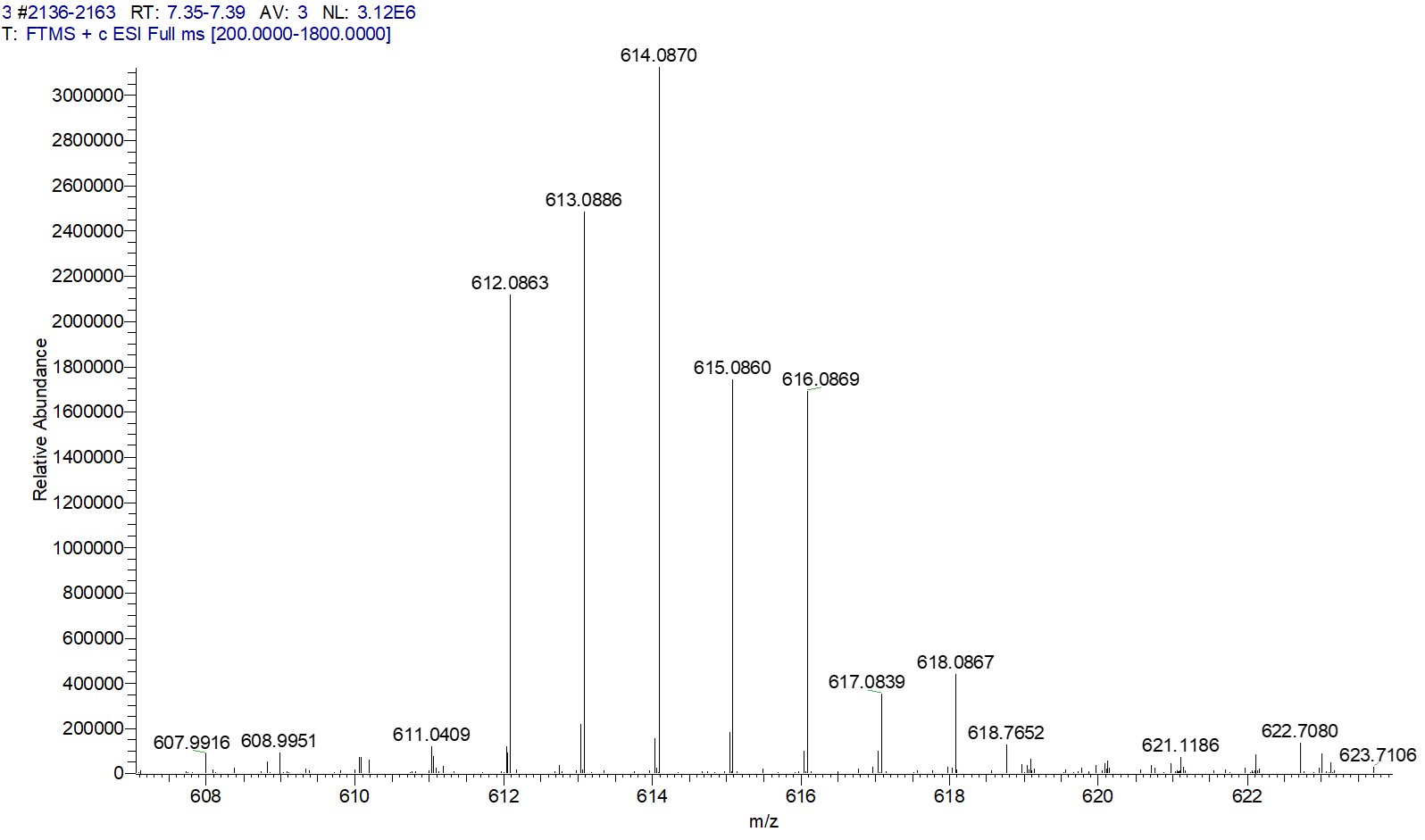
**Fig. S7**. HRMS spectra of intermediate **2**.


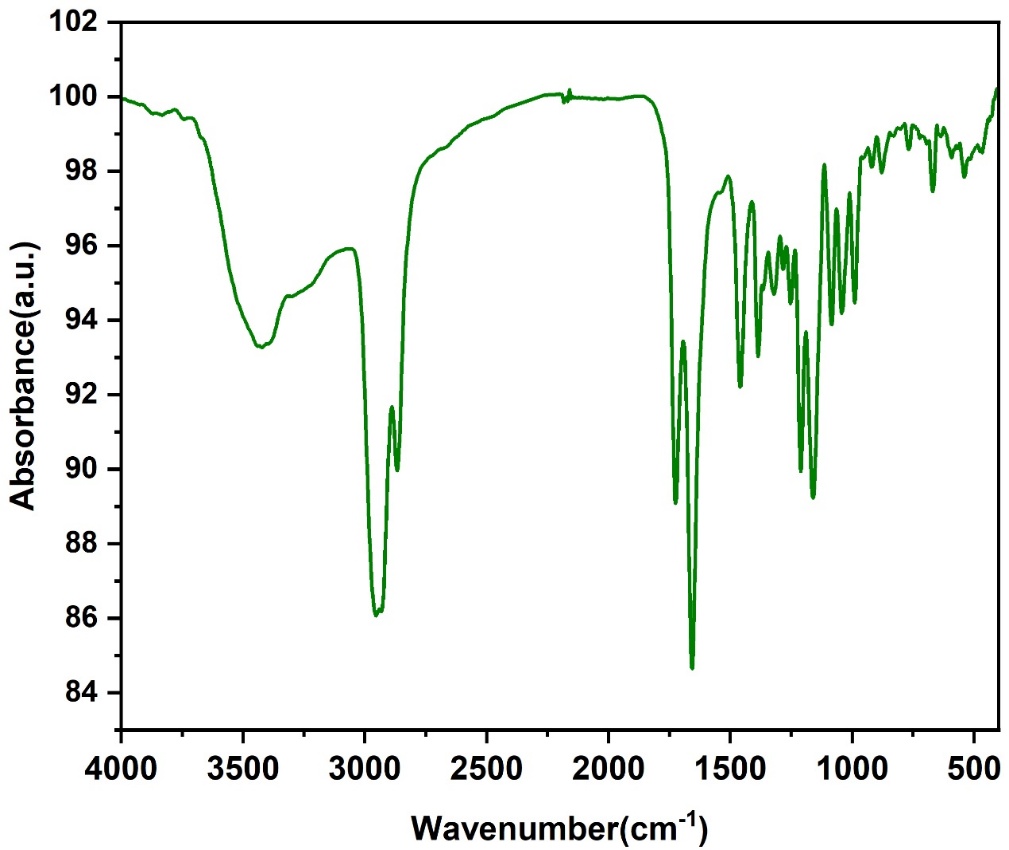
**Fig. S8**. FT-IR spectra of intermediate **3**



**Fig. S9**. ^1^H NMR spectra of intermediate **3.**



**Fig. S10**. ^13^C NMR spectra of intermediate **3.**

**Fig. S11**. HRMS spectra of intermediate **3.**


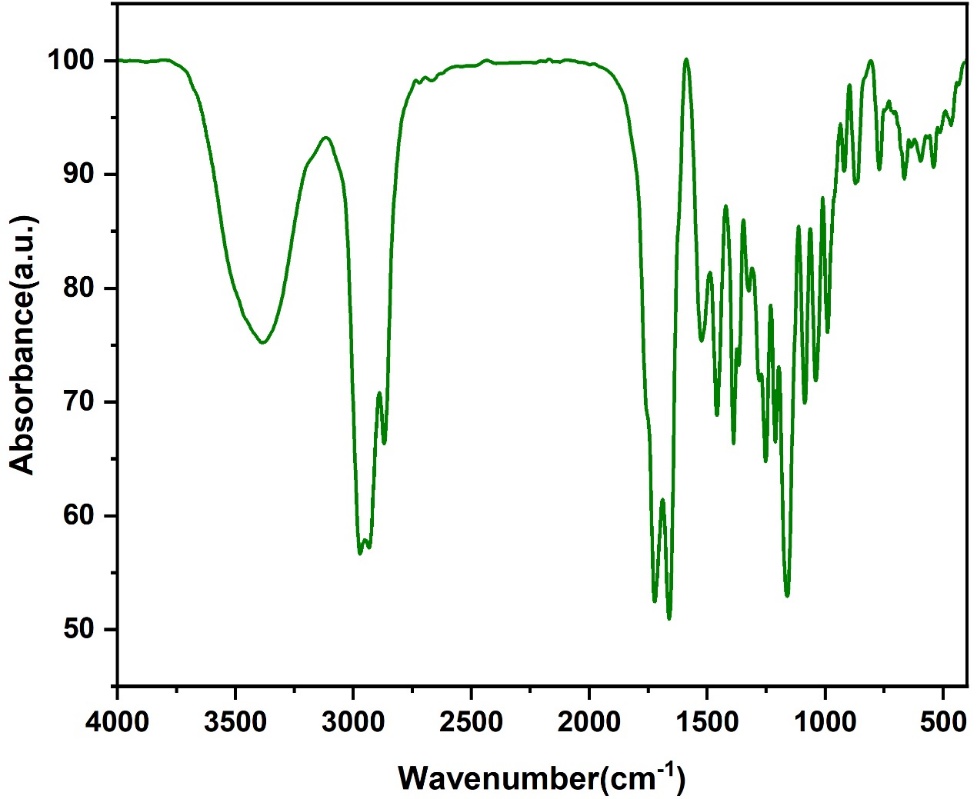
**Fig. S12**. FT-IR spectra of intermediate **4.**


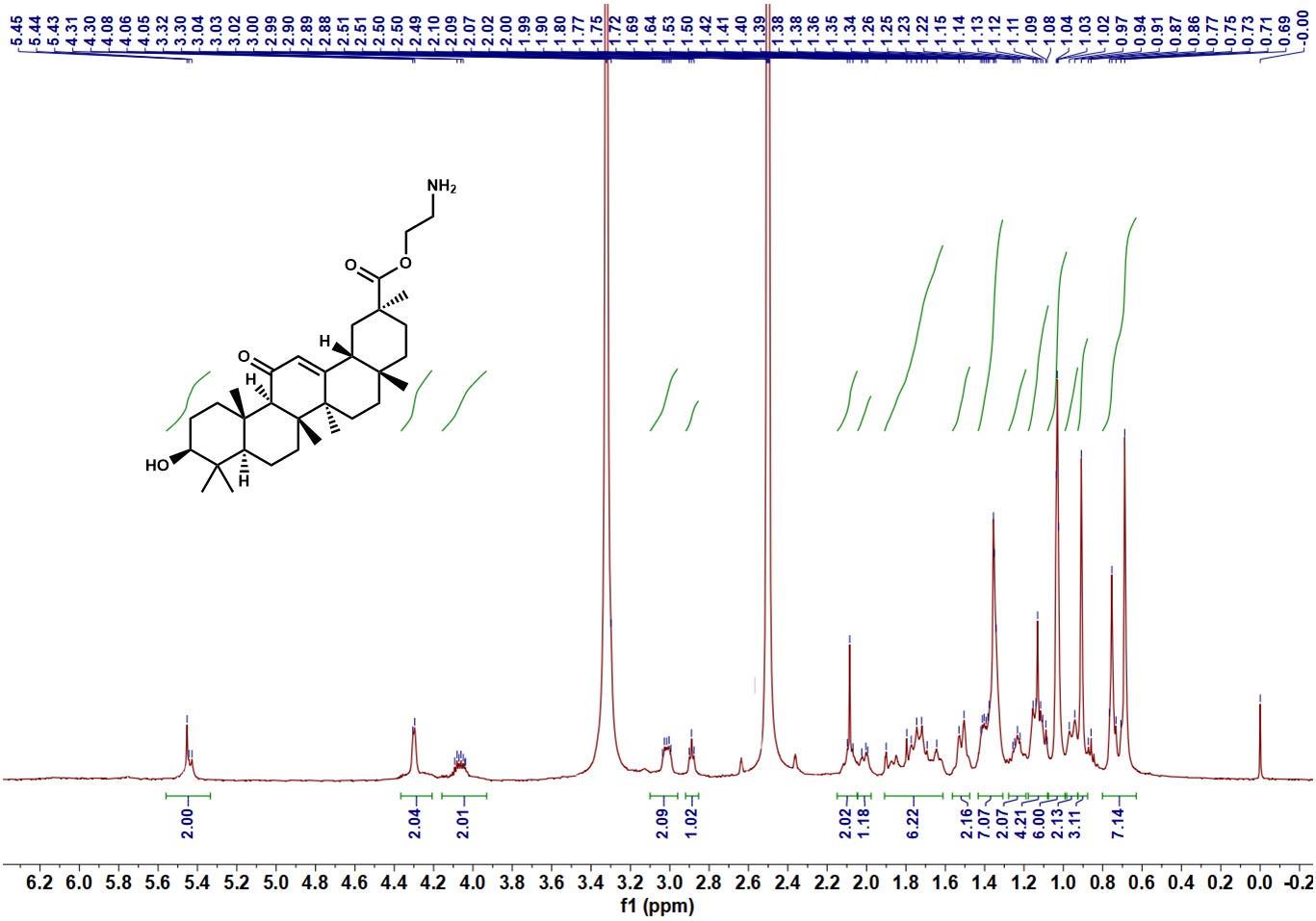
**Fig. S13**. ^1^H NMR spectra of intermediate **4.**


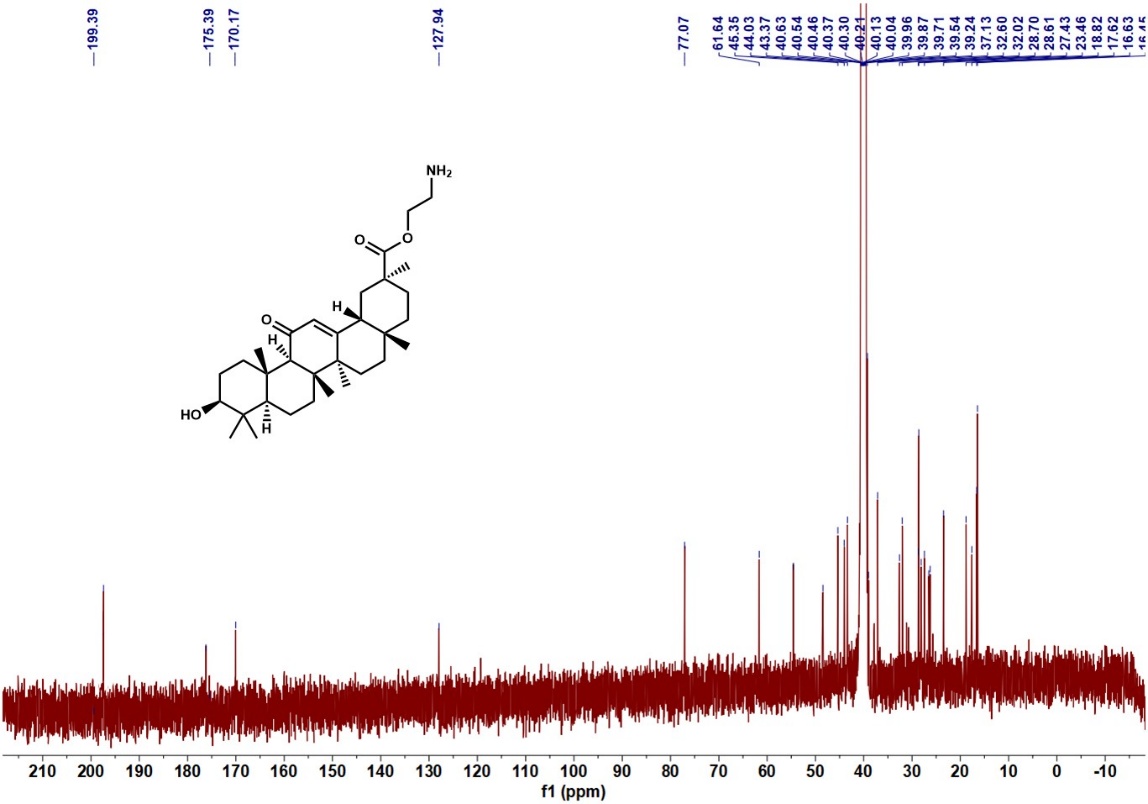
**Fig. S14**. ^13^C NMR spectra of intermediate **4.**

^
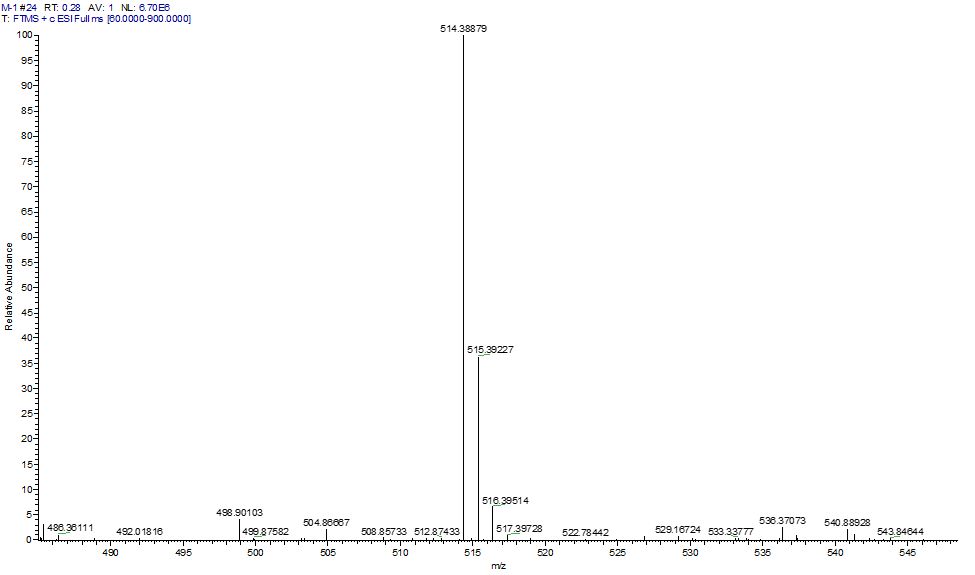
^**Fig. S15**. HRMS spectra of intermediate **4**


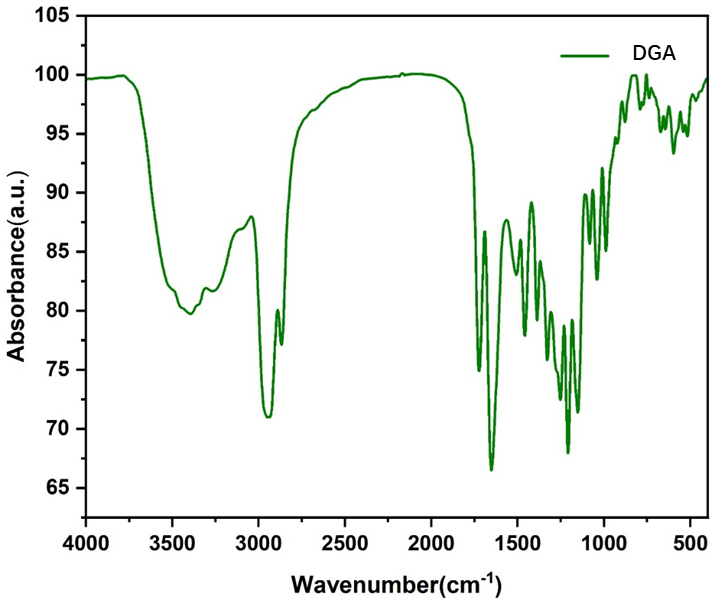
**Fig. S16**. FT-IR spectra of **DGA** conjugate.


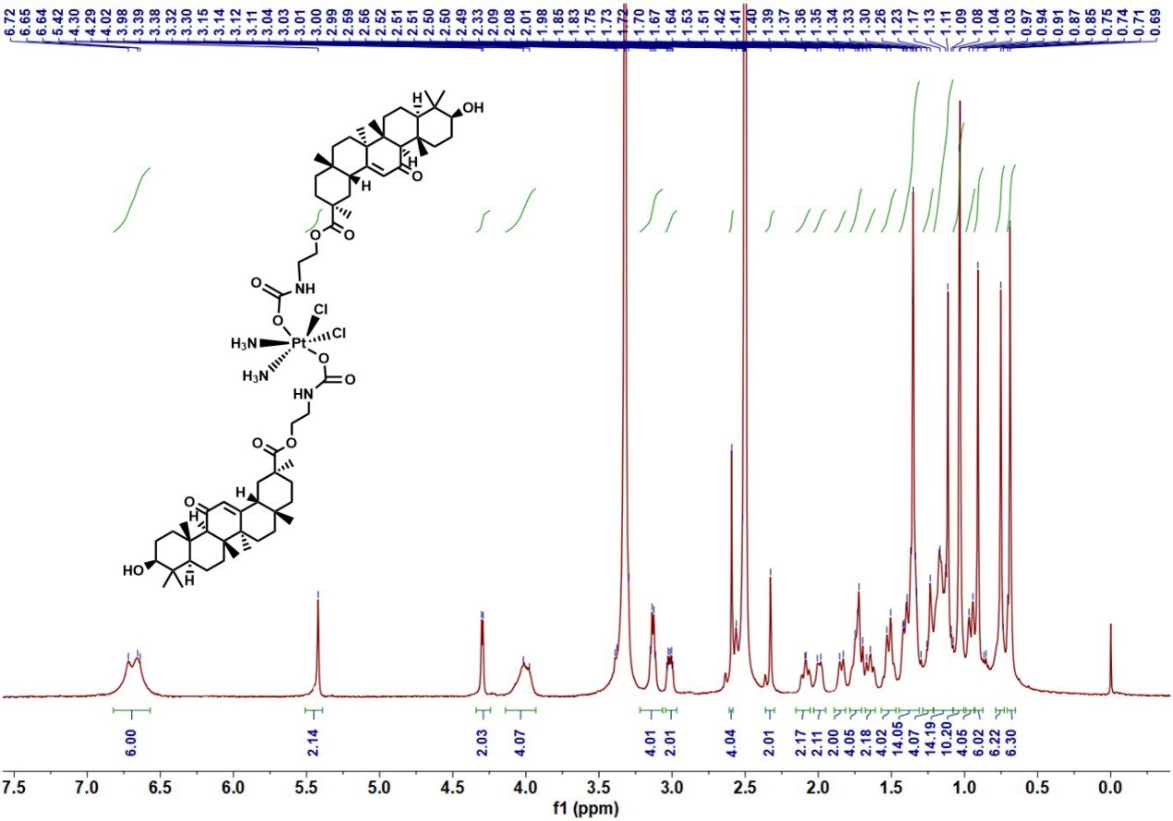


**Fig. S17**. ^1^H NMR spectra of **DGA** conjugate**.**


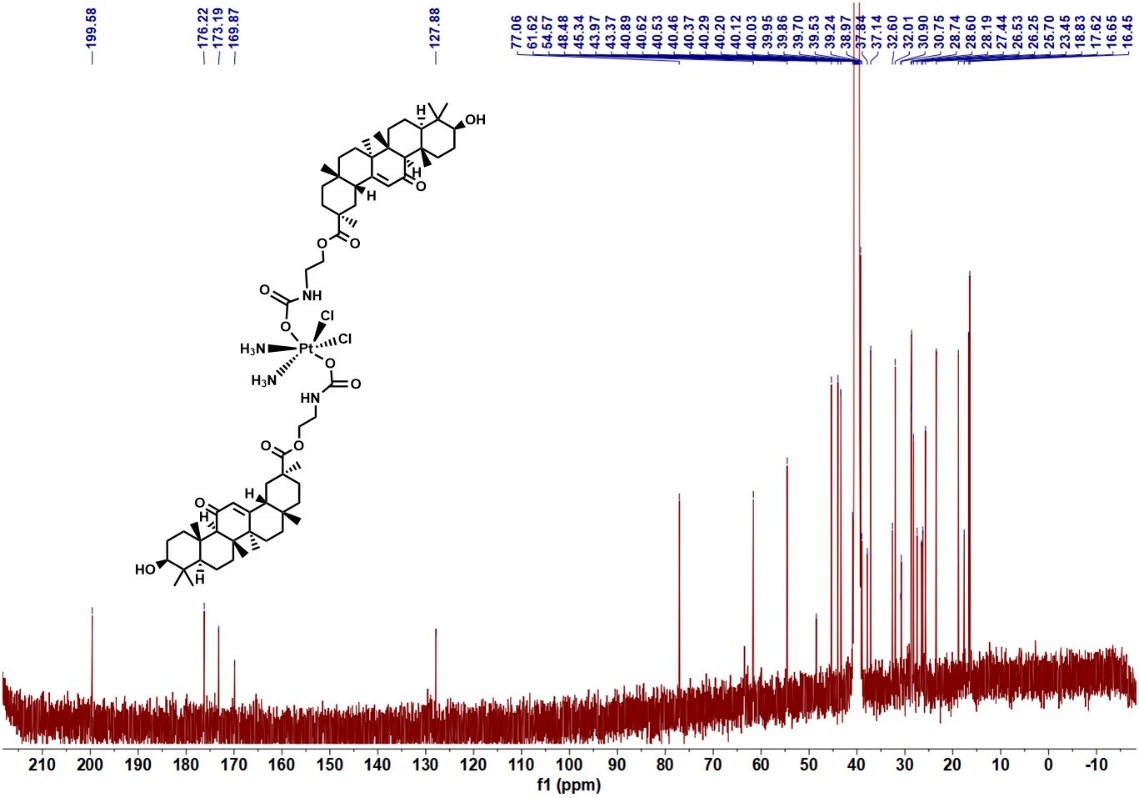
**Fig. S18**. ^13^C NMR spectra of **DGA** conjugate.


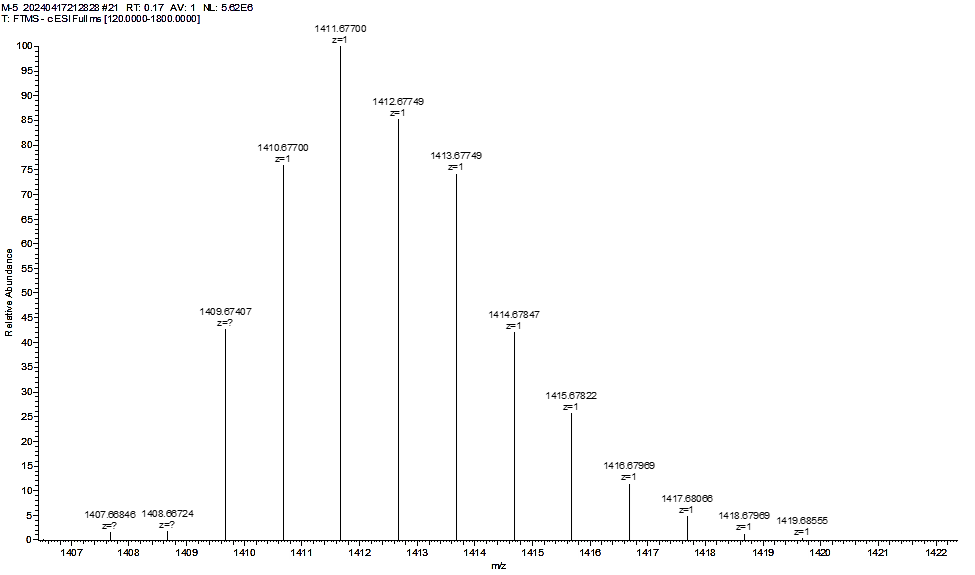


**Fig. S19**. HRMS spectra of **DGA** conjugate.


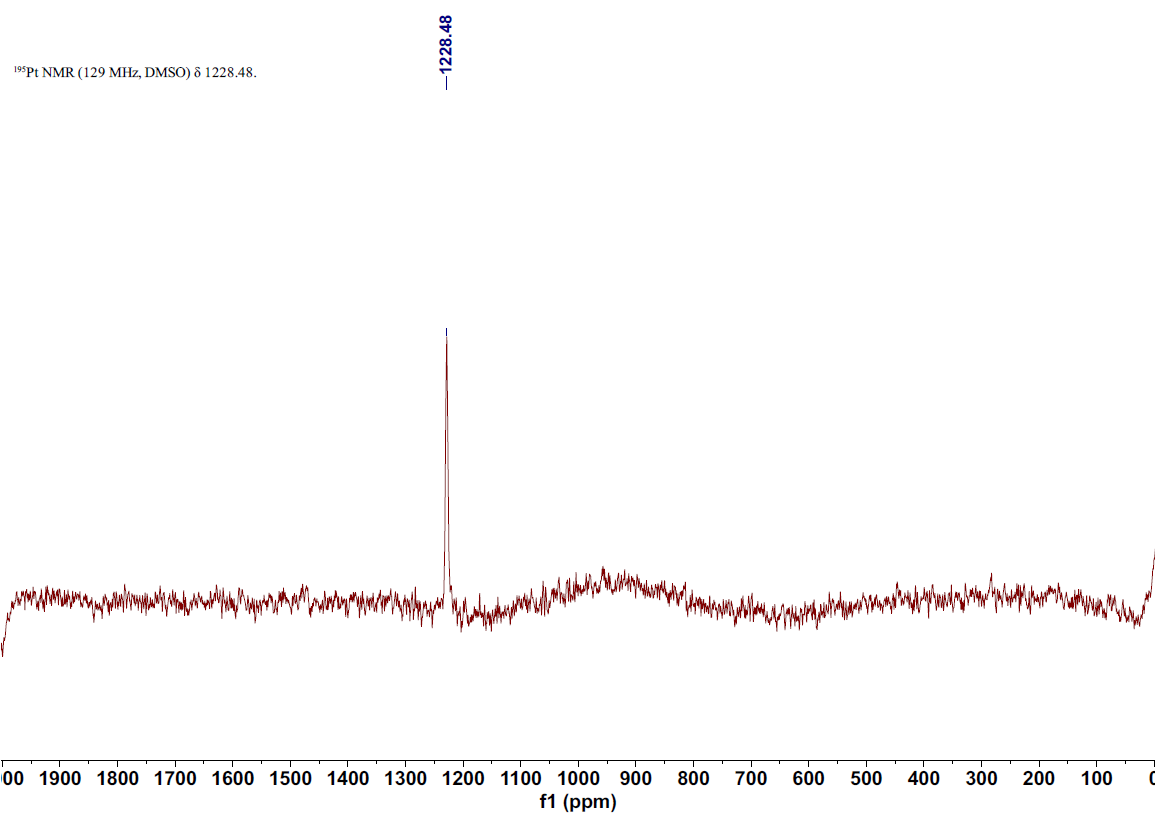
**Fig. S20**. ^195^Pt spectra of **DGA** conjugate.


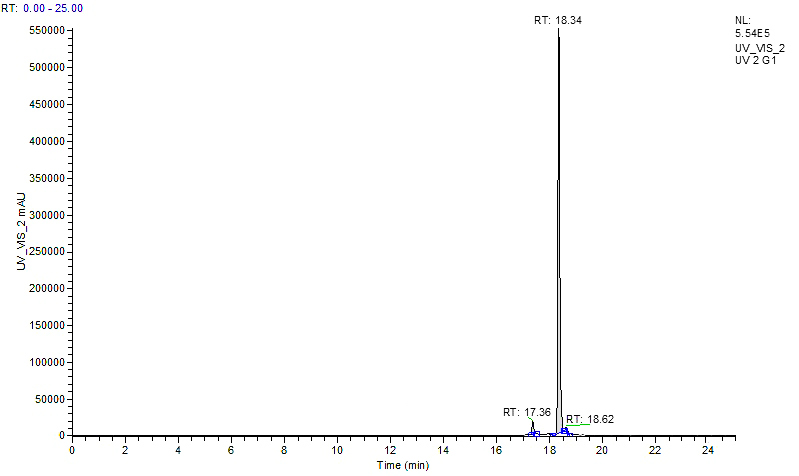
**Fig. S21**. HPLC spectra and of **DGA** conjugate.

**Table S1**. The purity of **DGA** conjugate.

| Apex RT | Start RT | End RT | Area %Area | Height | %Area |
| --- | --- | --- | --- | --- | --- |
| 17.36 | 17.30 | 17.51 | 88312.354 | 2.91 | 3.02 |
| 18.34 | 18.12 | 18.53 | 2906926.634 | 95.64 | 95.79 |
| 18.62 | 18.57 | 18.76 | 44282.567 | 1.46 | 1.19 |


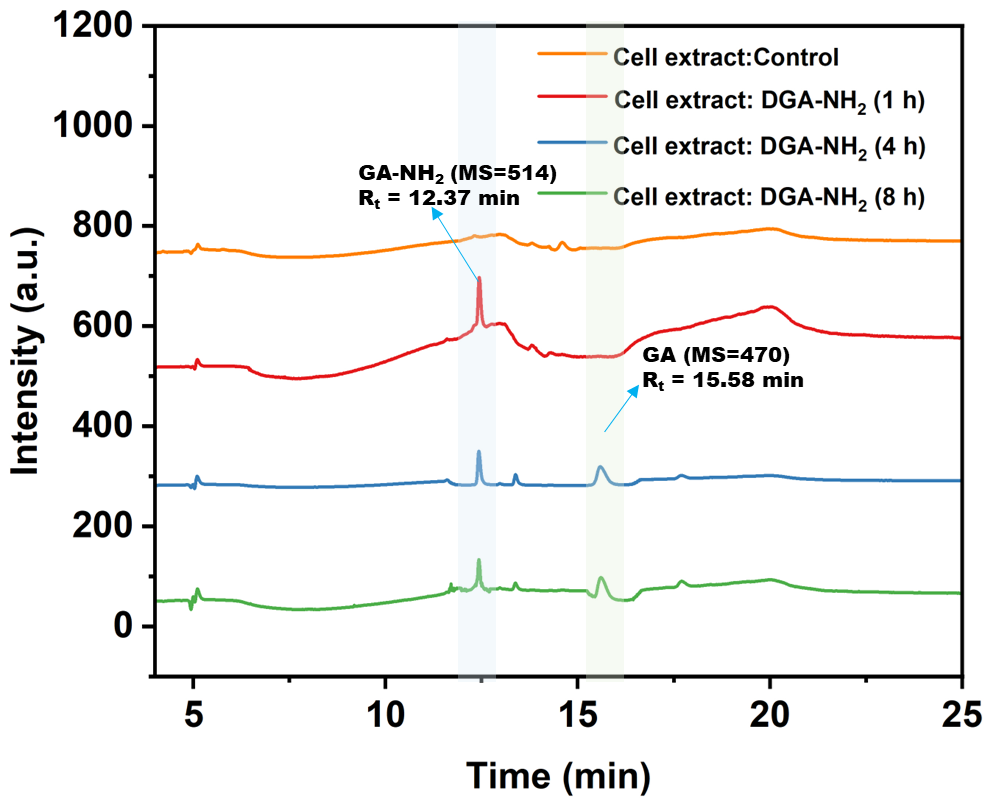
**Fig. S22.** LCMS analysis of GA-NH_2_ hydrolysis to GA in HepG2 cells at 2, 4 and 8 h.


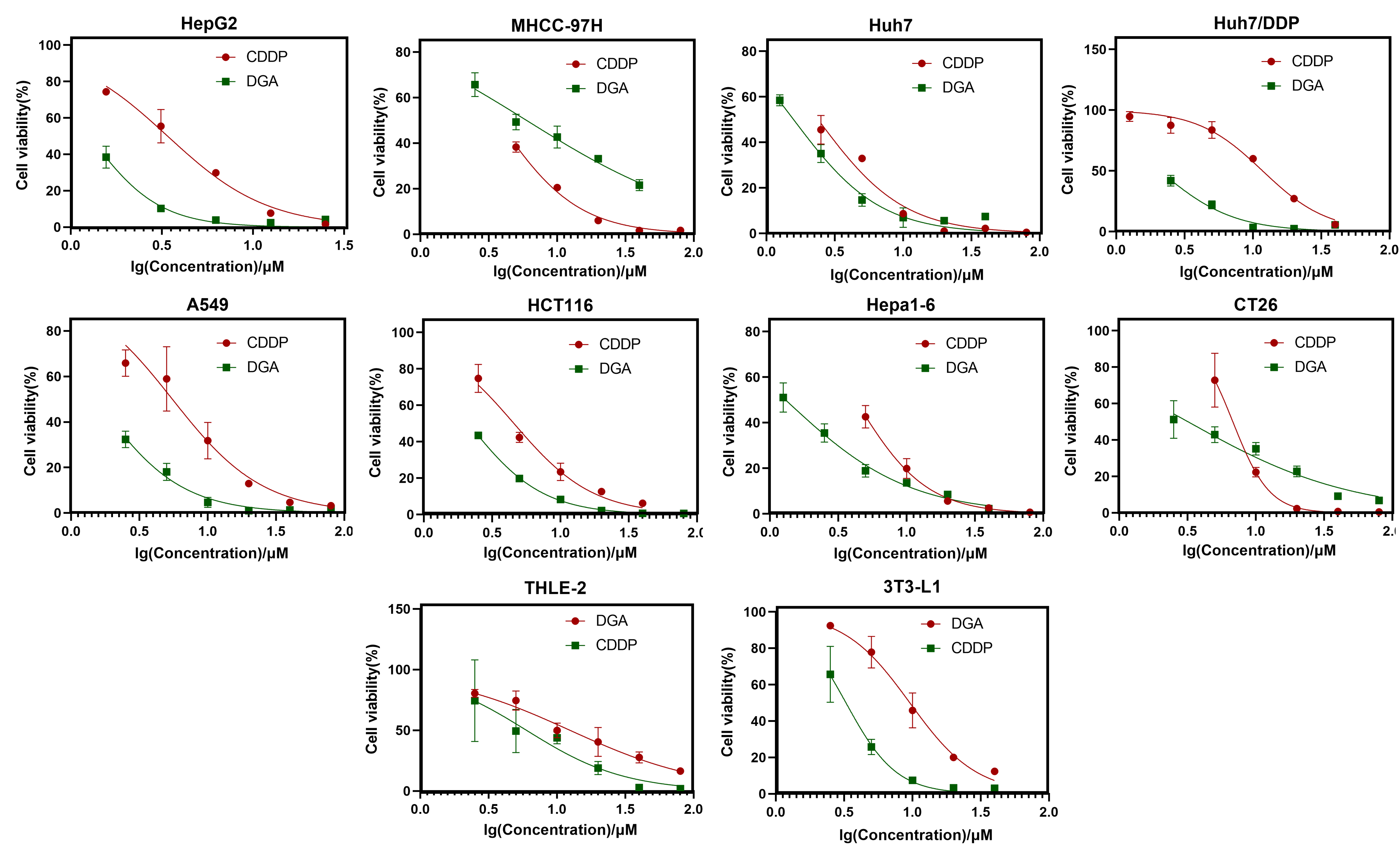
**Fig. S23.** CCK8 assay showing the biological activity (IC_50_ curves) of CDDP and **DGA** NPs on different cell lines.

**
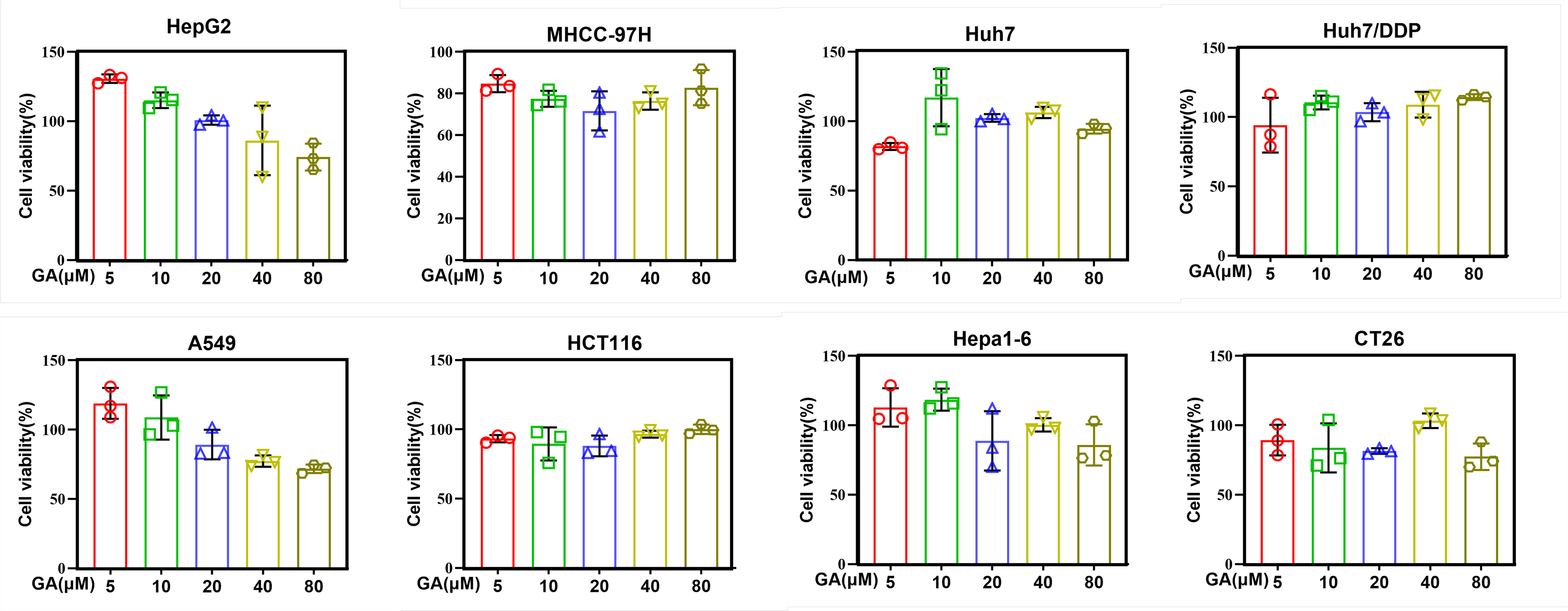
Fig. S24.** Bar plots depicting cell viability as a function of GA concentration.

**
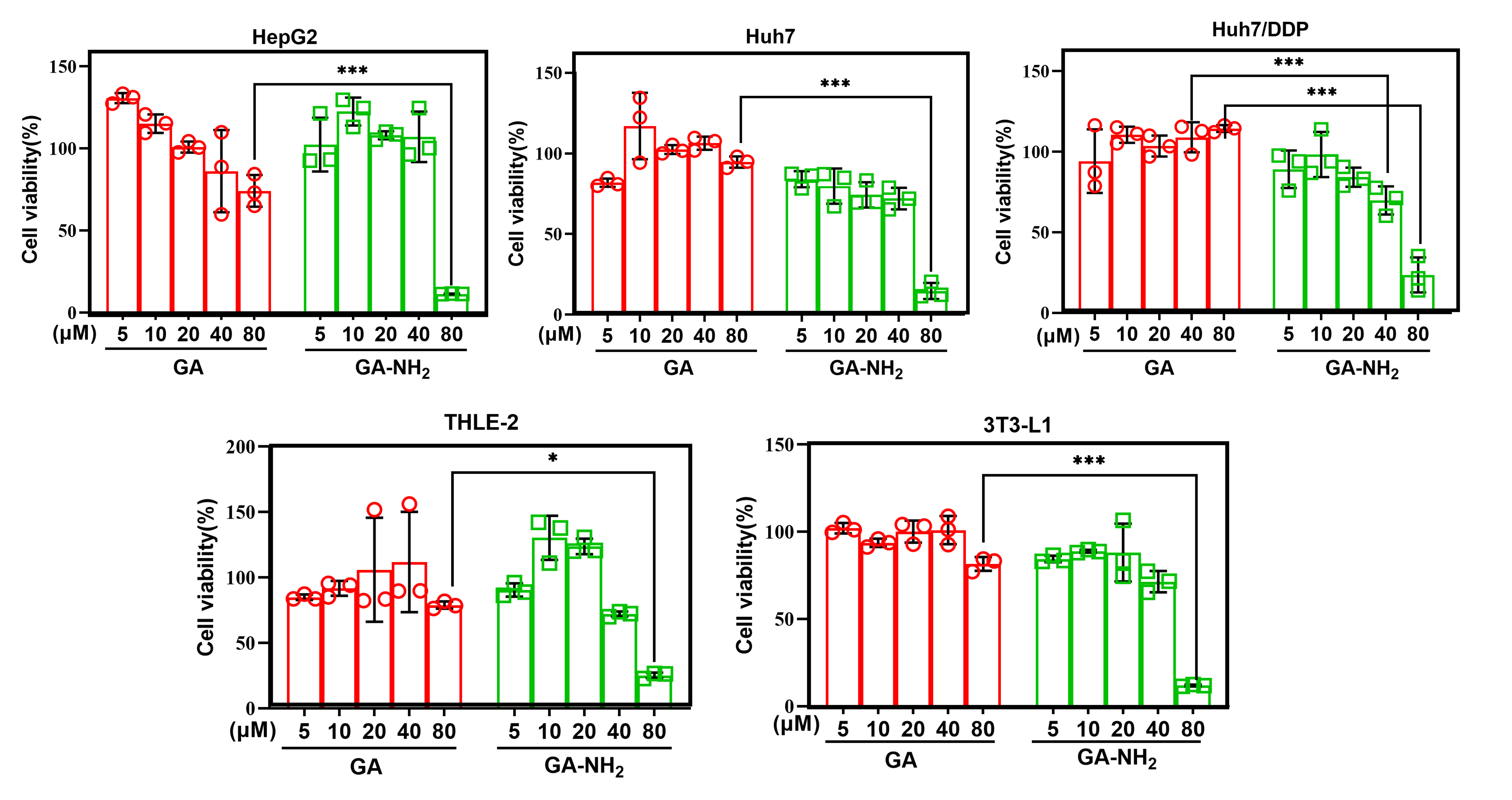
Fig. S25.** Bar plots depicting cell viability as a function of GA-NH_2_ concentration. Data are presented as mean ± SD (n = 3 biologically independent experiments). *p < 0.05, ***p < 0.001 by ANOVA.

**
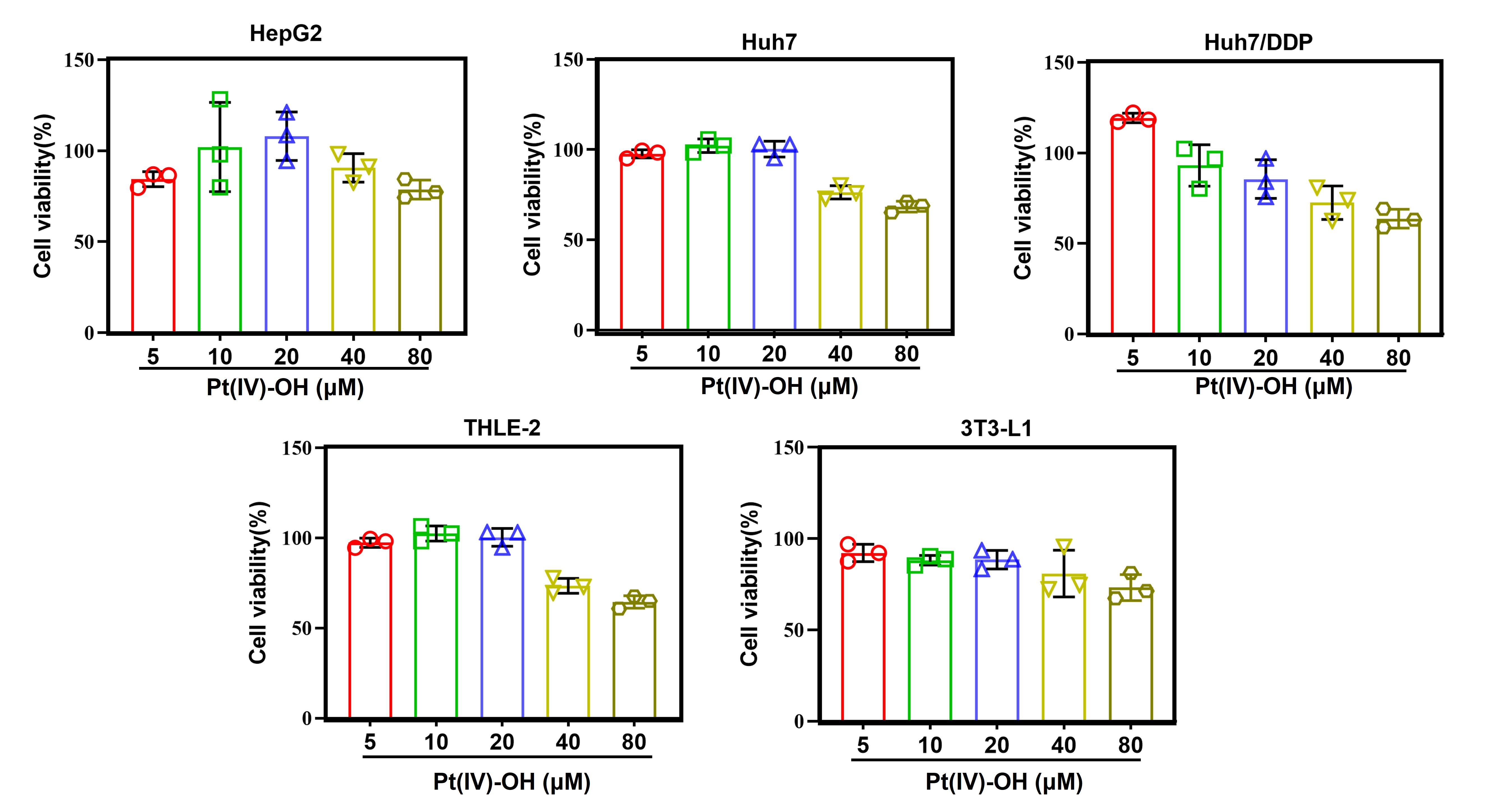
Fig. S26.** Bar plots depicting cell viability as a function of Pt(IV)-OH concentration. Data are presented as mean ± SD (n = 3 biologically independent experiments).

**Table S2.** IC_50_ (μM) of these compounds against different cell lines at 96 h^a^

| **Compounds** | **HepG2** | **Huh7** | **Huh7/DDP** | **THLE-2** | **3T3-L1** |
| --- | --- | --- | --- | --- | --- |
| **CDDP** | 5.26 ± 2.06 | 2.29 ± 0.09 | 11.45 ± 0.81 | 3.61 ± 1.78 | 4.52 ± 1.34 |
| **Pt(IV)-OH** | > 80 | > 80 | > 80 | > 80 | > 80 |
| **GA** | > 80 | > 80 | > 80 | > 80 | > 80 |
| **GA-NH_2_** | > 40 | > 40 | > 40 | > 40 | > 40 |
| **CDDP+GA^b^** | 3.27 ± 0.45 | 3.42 ± 1.40 | 11.48 ± 0.37 | 3.49 ± 1.53 | 3.32 ± 2.31 |
| **CDDP+GA-NH_2_^c^** | 3.85 ± 2.08 | 3.54 ± 1.48 | 9.87 ± 2.68 | 3.08 ± 1.92 | 3.77 ± 1.86 |
| **DGA** NPs | 2.98 ± 1.73 | 1.54 ± 0.79 | 1.18 ± 0.78 | 11.44 ± 1.74 | 8.37 ± 0.55 |

*^a^ IC_50_ values are represented as mean ± SD (n = 3 biologically independent experiments).*

*^b^ The combo of CDDP and GA (1:2).*

*^c^ The combo of CDDP and GA-NH_2_ (1:2).*

**
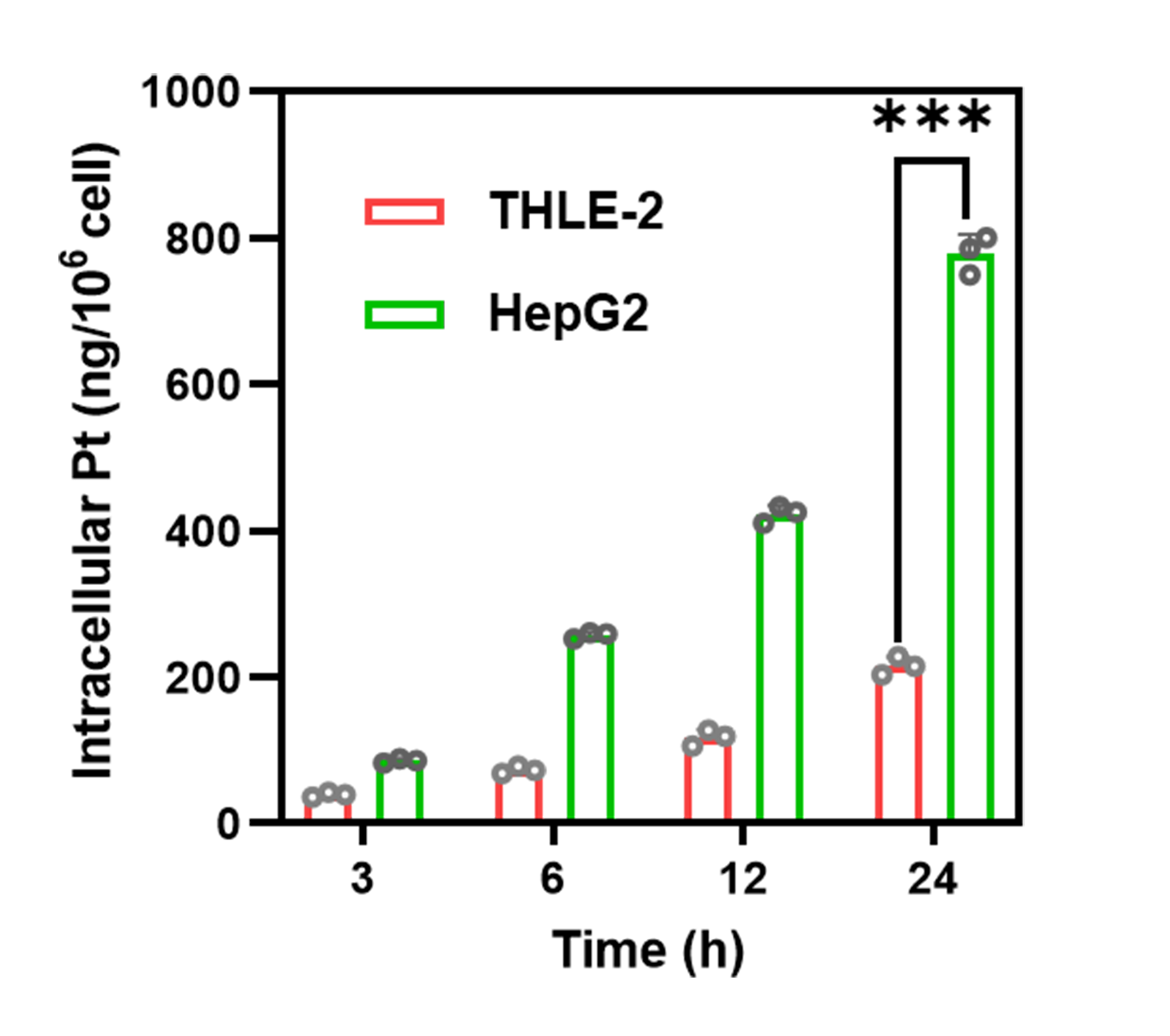
Fig. S27.** Intracellular platinum content in THLE-2 and HepG2 cells after treatment with **DGA** NPs (5 μM) for 0-24 h. Data are presented as mean ± SD (n = 3 biologically independent experiments). ***P < 0.001 (one way ANOVA).


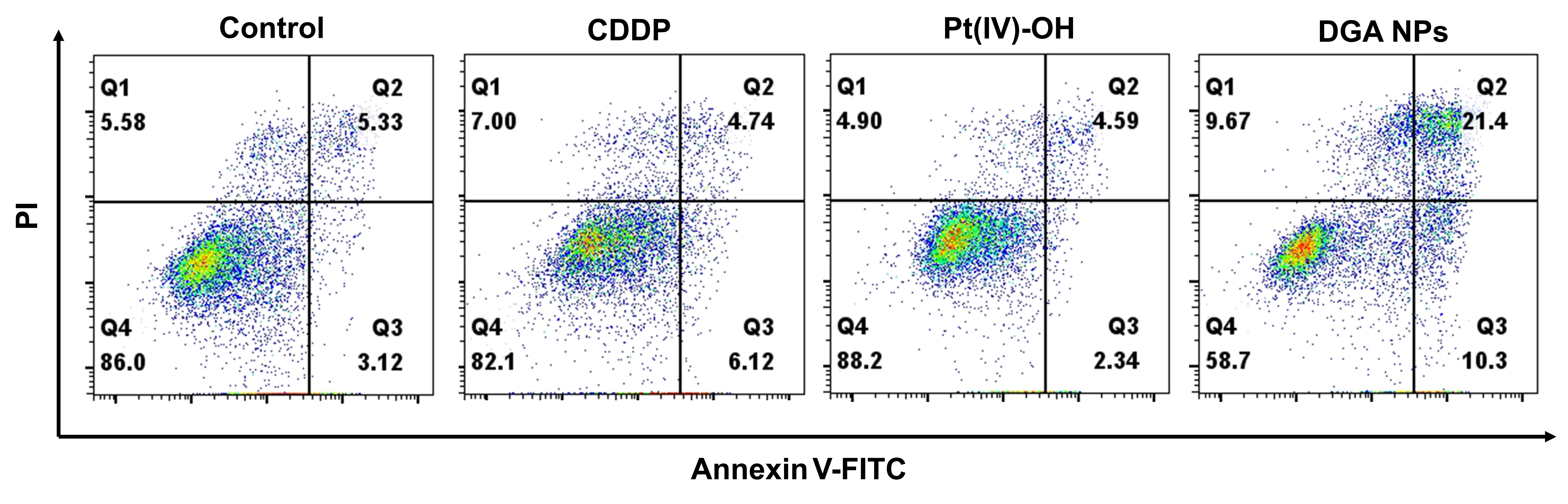
**Fig. S28.** Apoptosis analysis in Cisplatin-resistant cell line Huh7/DDP cells treated with CDDP (5 μM), Pt(IV)-OH (5 μM), and **DGA** NPs (5 μM) for 48 h.


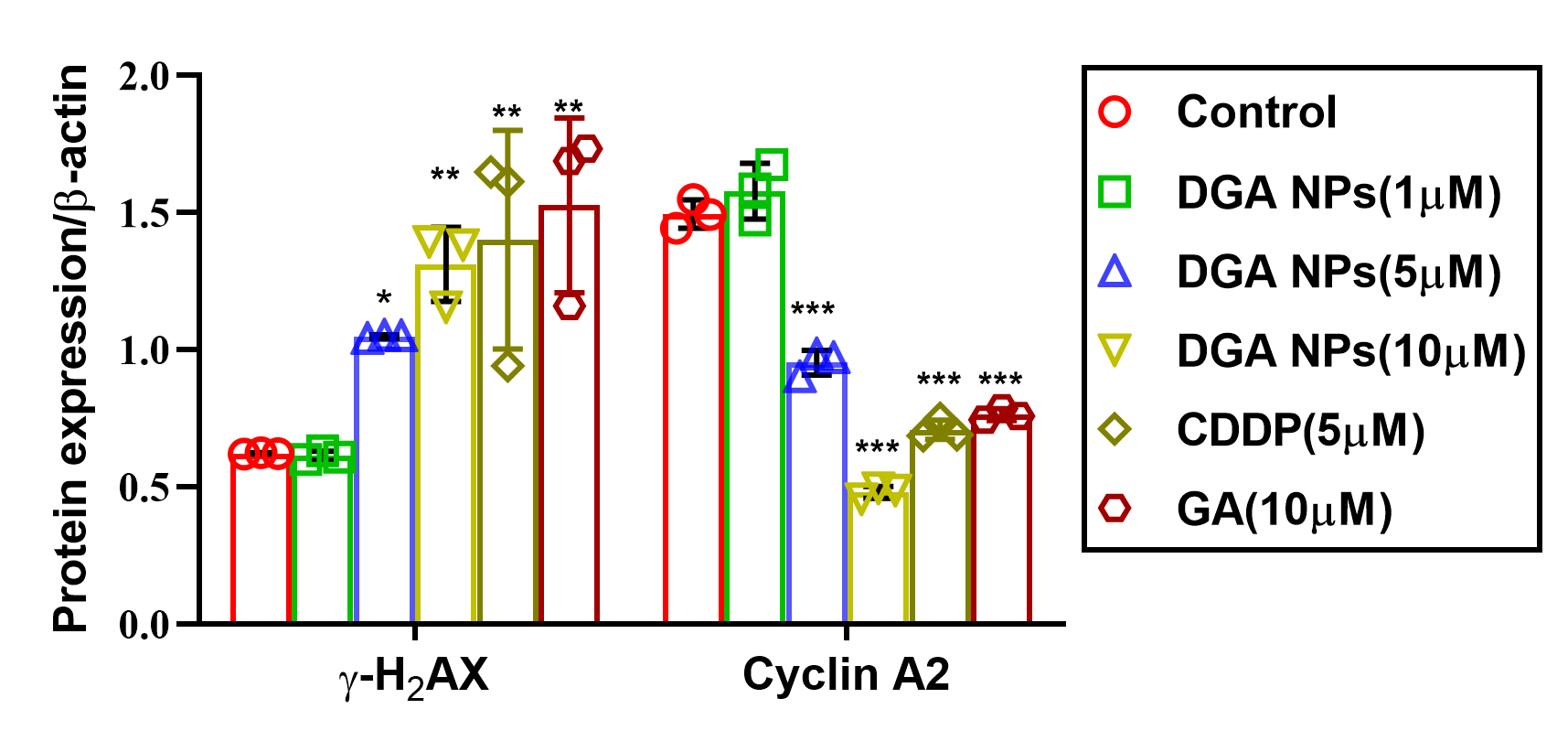
**Fig. S29.** Relative density of γ-H_2_AX and Cyclin A2 to β-actin**.** Data are presented as mean ± SD. Representative blots from three independent experiments. *p < 0.05, **p < 0.01, ***p < 0.001 by one-way ANOVA.


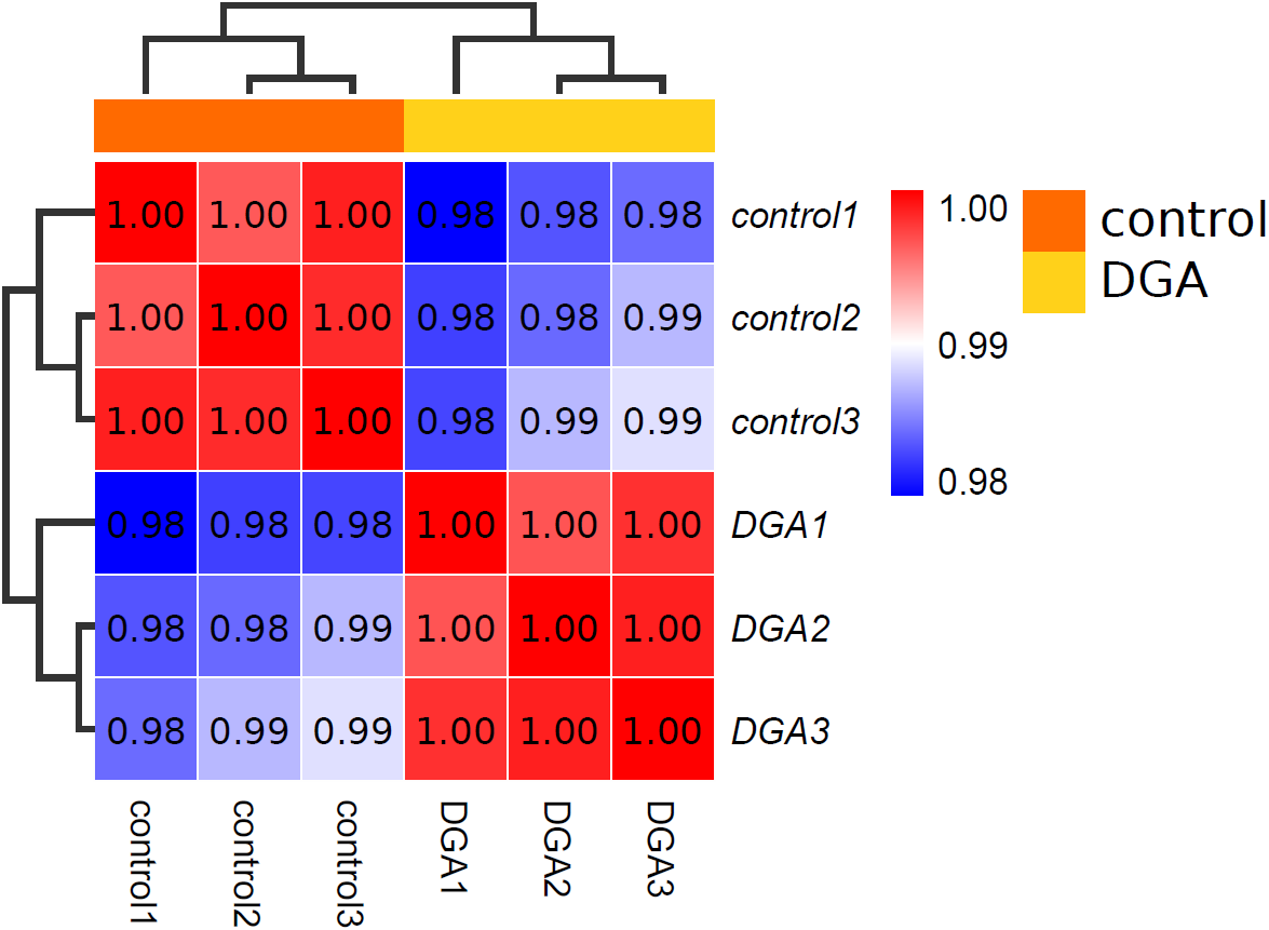
**Fig. S30.** Heat map diagram of Pearson correlation coefficients between **DGA** NPs and control RNA-seq samples.


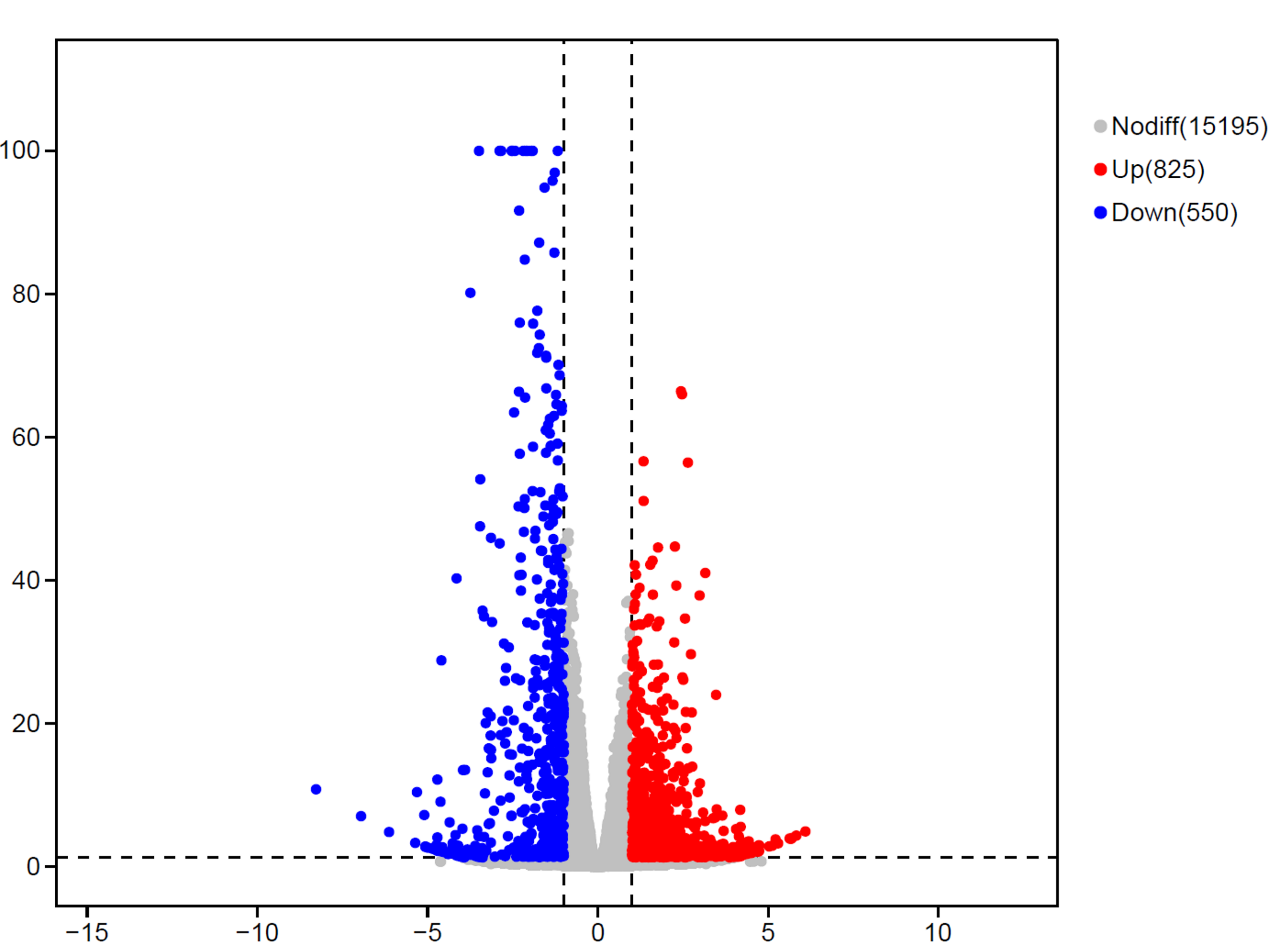
**Fig. S31.** The number of significantly different genes in **DGA** NPs treated group.

**Table S3.** Statistics of RNA-Seq for the control and the **DGA** NPs-treated groups

| Sample | Clean_Reads | Total Mapped |
| --- | --- | --- |
| control1 | 49947258 | 47419717 (94.94%) |
| control2 | 51938916 | 49348437 (95.01%) |
| control3 | 42939352 | 40754753 (94.91%) |
| **DGA**-NPs 1 | 48348076 | 46475981 (96.13%) |
| **DGA**-NPs 2 | 46367476 | 44640736 (96.28%) |
| **DGA**-NPs 3 | 39941284 | 38384361 (96.10%) |

**Table S4.** Primer sequences used for qPCR analysis.

| **Gene** | **Primer sequence** | |
| --- | --- | --- |
|  | **Forward** | **Reverse** |
| **PIK3CA** | CCACGACCATCATCAGGTGAA | CCTCACGGAGGCATTCTAAAGT |
| **PIK3R1** | GCCAGTACCGTGTGGACAAT | TGCTGTGGGAGATAGCTGTG |
| **AKT1** | AGCGACGTGGCTATTGTGAAG | GCCATCATTCTTGAGGAGGAAGT |
| **p53** | TTACTCCCTCCATCTCCACC | TCATCAAACCCTTCAGCCAG |
| **BAX** | TGGAGCTGCAGAGGATGATTG | GAAGTTGCCGTCAGAAAACATG |
| **Bcl-2** | CCTGTGCACCAAGGTGCCGGAACT | CCACCCTGGTCTTGGATCCAGCC |
| **Caspase3** | GAGGCGGTTGTAGAAGAGTTCGTG | TGGGGGAAGAGGCAGGTGCA |
| **Caspase9** | GTGGACATTGGTTCTGGAGGAT | GTGGACATTGGTTCTGGAGGAT |
| **GAPDH** | GGAGCGAGATCCCTCCAAAAT | GGCTGTTGTCATACTTCTCATGG |


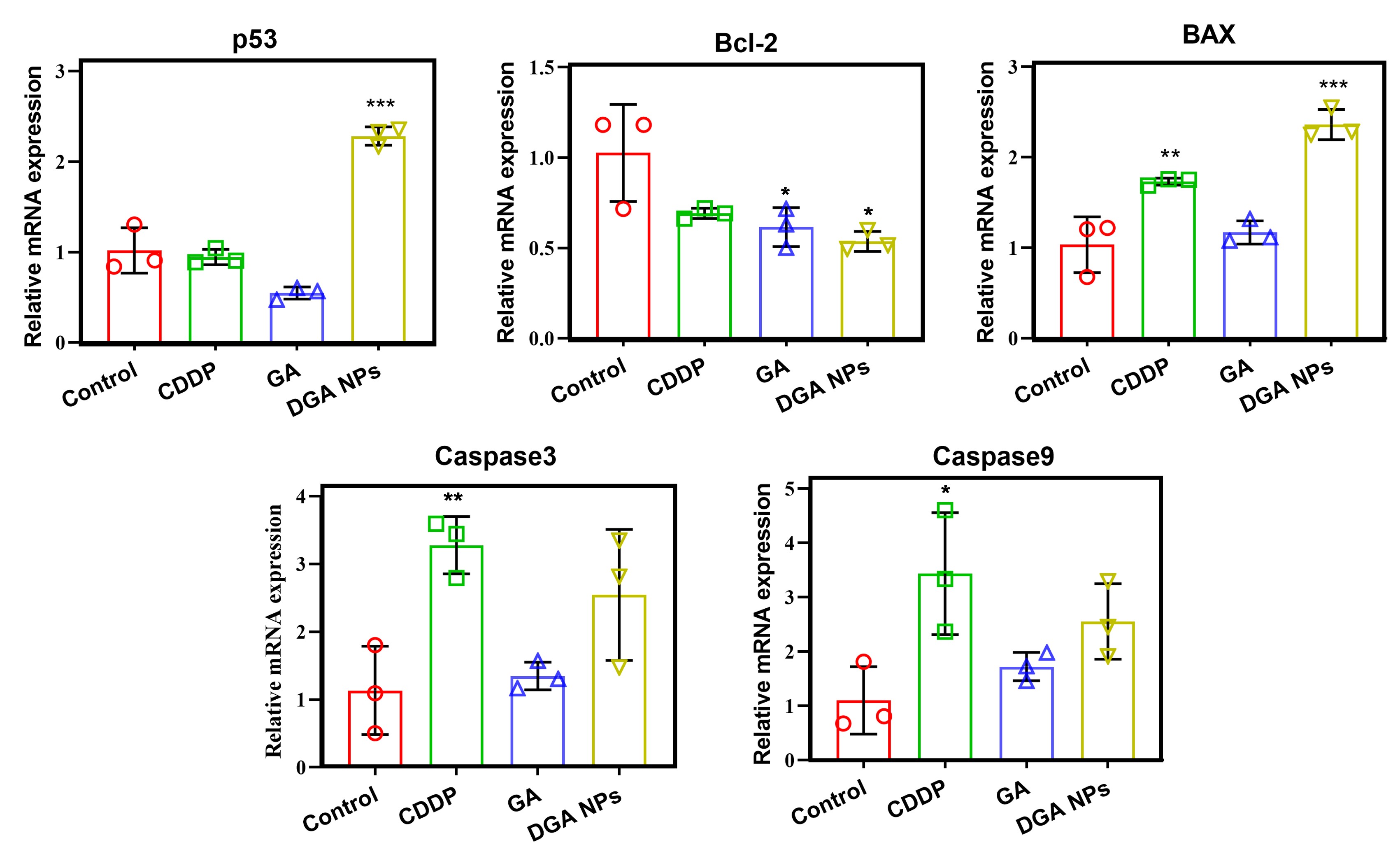
**Fig. S32.** Detection of mRNA levels of p53 pathway-related genes in HepG2 cells after treatment with CDDP (5 μM), GA (10 μM) or **DGA** NPs (5 μM) for 48 hours. Data are presented as mean ± SD (n = 3 biologically independent experiments). *p < 0.05, **p < 0.01, ***p < 0.001 by ANOVA.


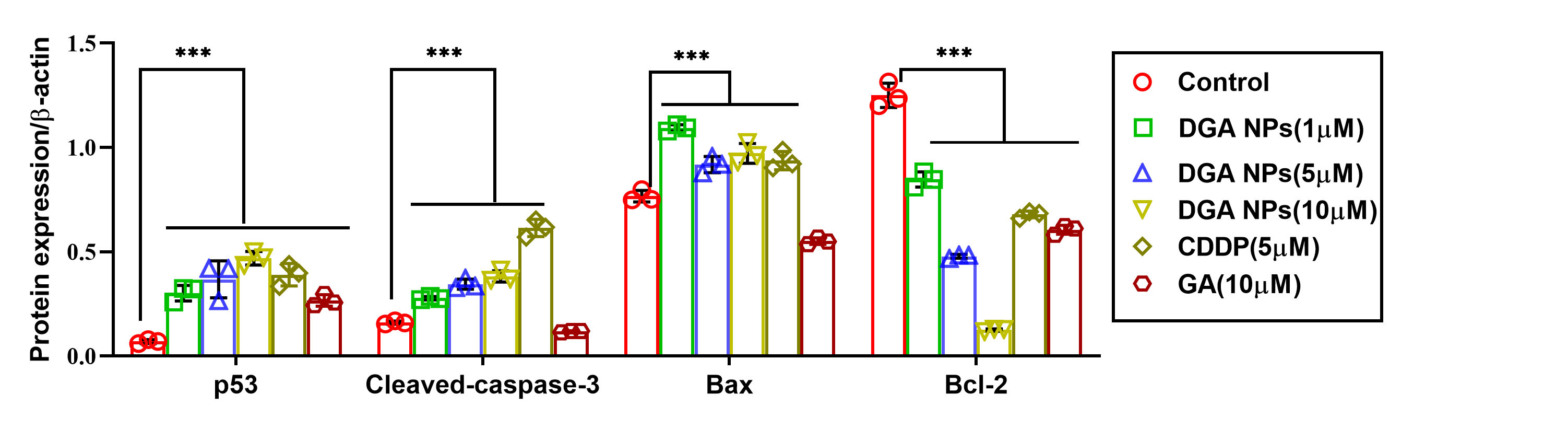
**Fig. S33.** Relative density of p53 and apoptosis-related protein to β-actin. Data are presented as mean ± SD. Representative blots from three independent experiments are shown. *p < 0.05, ** p < 0.01, *** p < 0.001 relative to the control group by ANOVA.


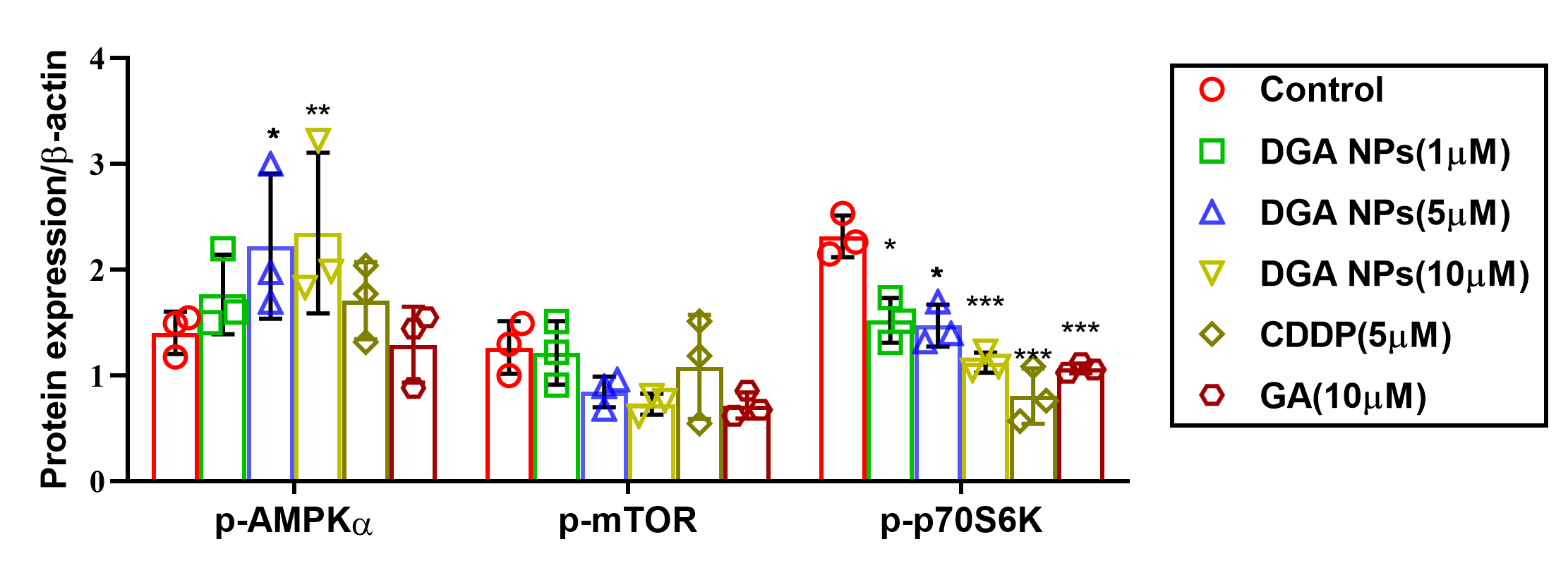


**Fig. S34.** Relative density of AMPK/mTOR/p70s6k pathway protein to β-actin. Data are presented as mean ± SD. Data are presented as mean ± SD. Representative blots from three independent experiments are shown. * p < 0.05, ** p < 0.01, *** p < 0.001 relative to the control group by ANOVA.


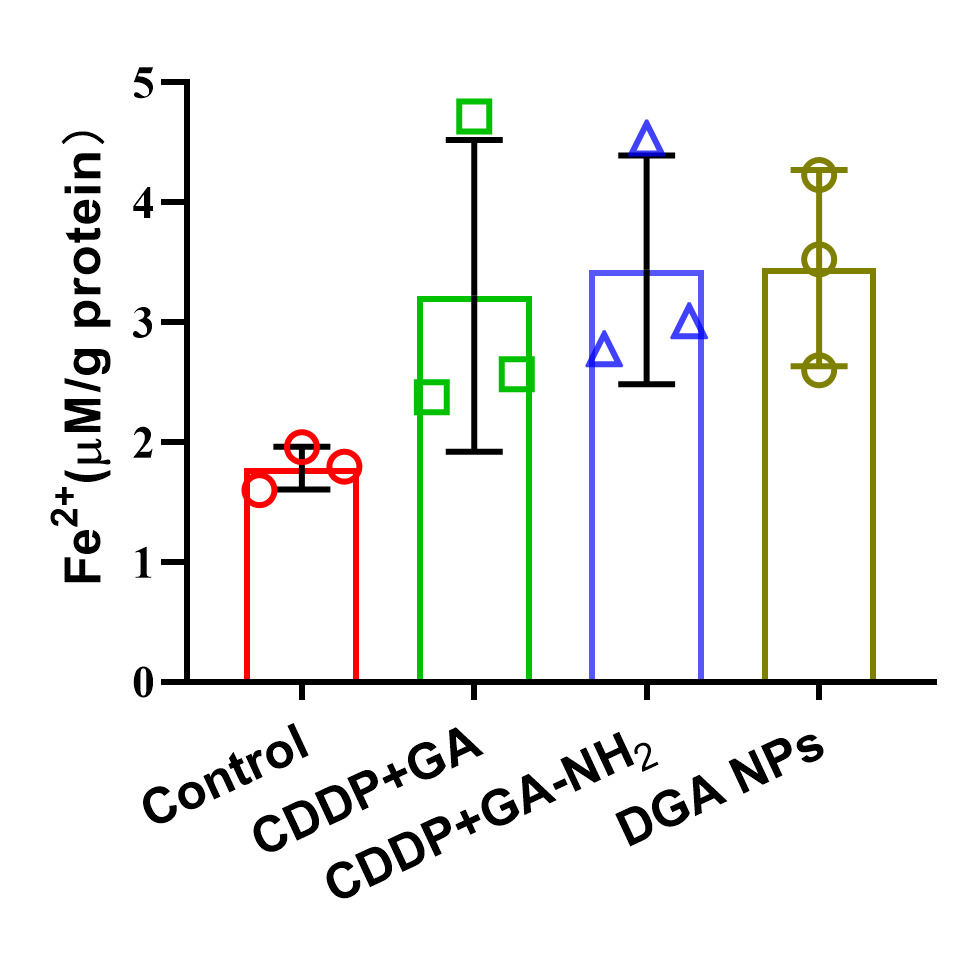
**Fig. S35**. Intracellular Fe²⁺ levels in HepG2 cells treated for 48 h with CDDP+GA (5 μM and 10μM), CDDP+GA-NH_2_ (5 μM and 10 μM) and **DGA** NPs (5 μM)**.** Data are presented as mean ± SD (n = 3 biologically independent experiments).


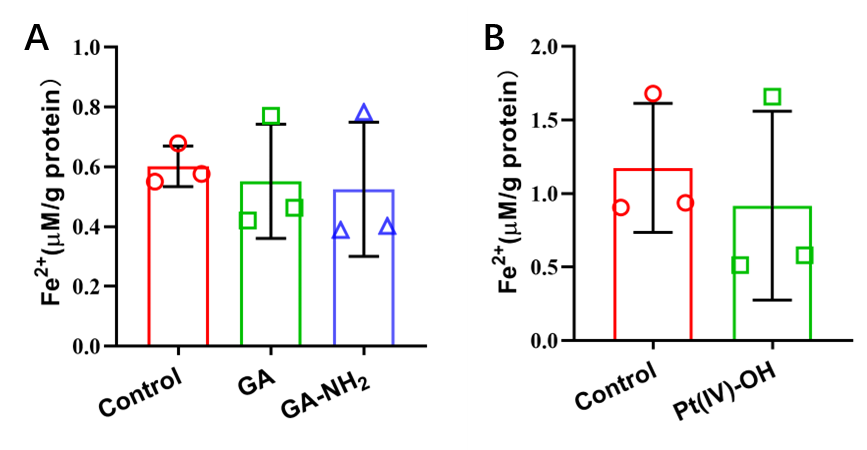
**Fig. S36.** Intracellular Fe²⁺ levels in HepG2 cells treated for 48 h with Pt(IV)-OH (5 μM). Data are presented as mean ± SD (n = 3 biologically independent experiments).


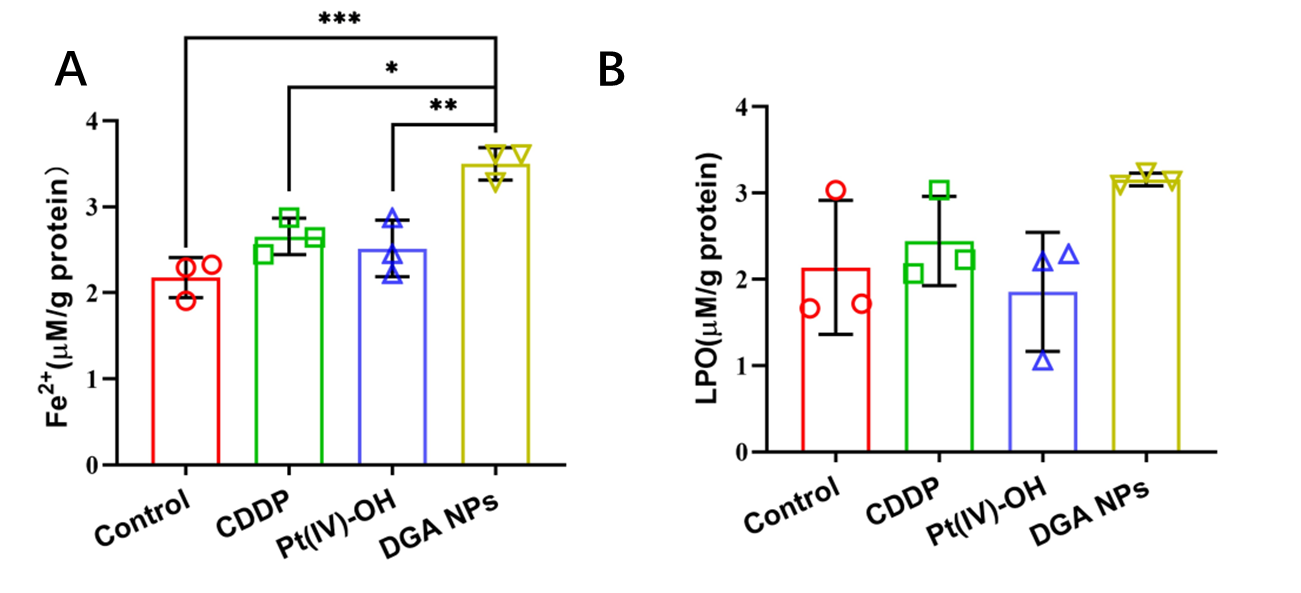
**Fig. S37. (A)** Intracellular Fe²⁺ and **(B)** LPO levels in cisplatin-resistant cell line Huh7/DDP cells treated for 48 h with CDDP (5 μM), GA (10 μM), or **DGA** NPs (5 μM). Data are presented as mean ± SD (n = 3 biologically independent experiments). *p < 0.05, **p < 0.01, ***p < 0.001 by ANOVA.


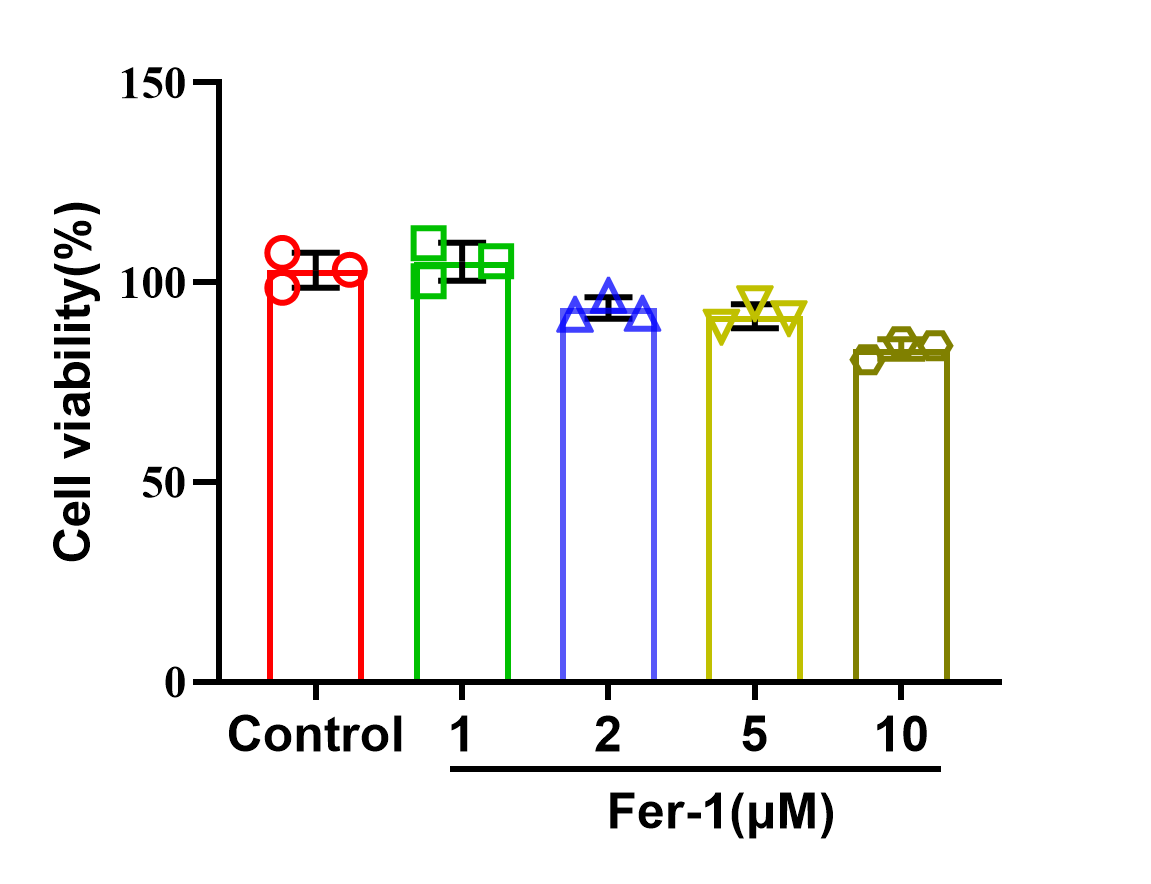
**Fig. S38**. Cell viability in HepG2 cells following 96-hour treatment with varying concentrations of Fer-1. Data are presented as mean ± SD (n = 3 biologically independent experiments).

**
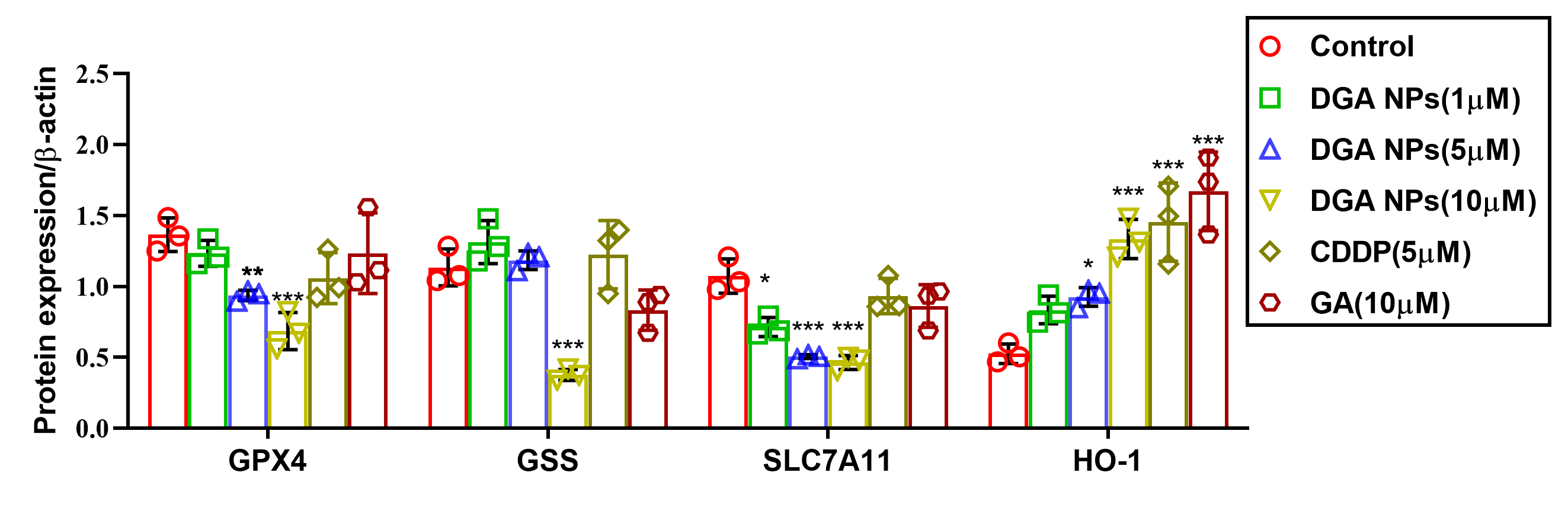
Fig. S39.** Relative density of ferroptosis-related proteins to β-actin. Data are presented as mean ± SD. Representative blots from three independent experiments are shown. * p < 0.05, ** p < 0.01, *** p < 0.001 relative to the control group by ANOVA.


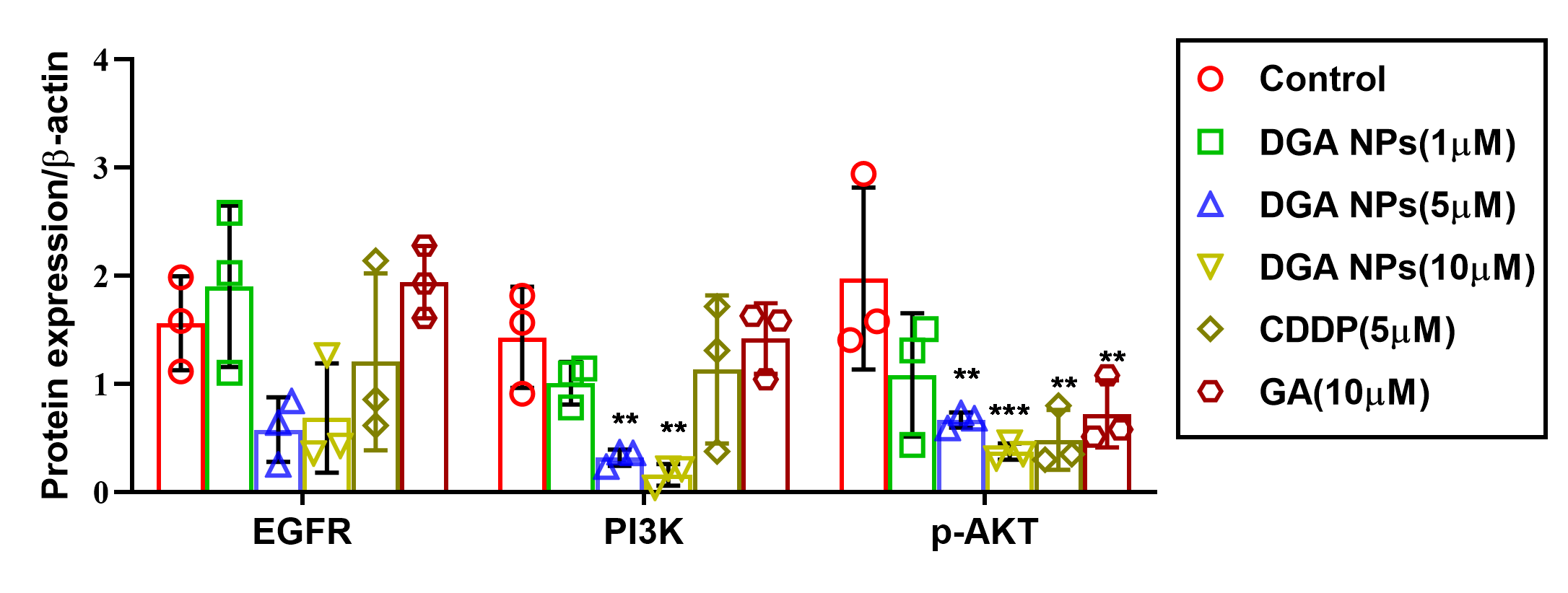


**Fig. S40.** Relative density of PI3K/AKT/mTOR pathway to β-actin. Data are presented as mean ± SD. Representative blots from three independent experiments are shown. *p < 0.05, **p < 0.01, ***p < 0.001 relative to the control group by ANOVA.


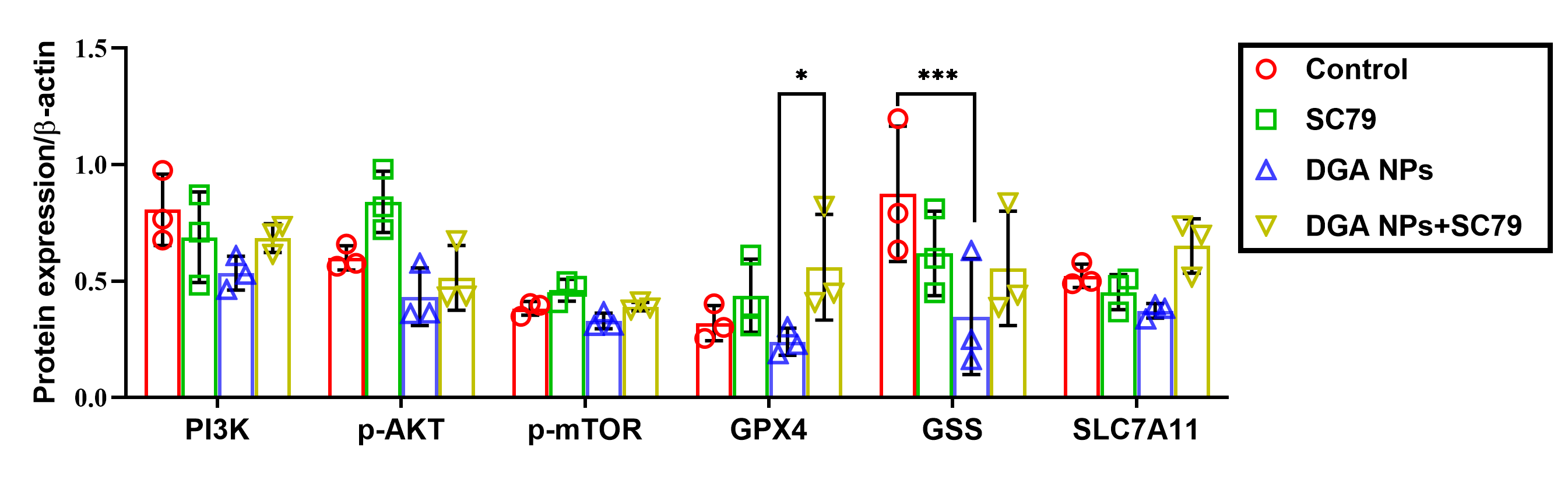
**Fig. S41.** Relative density of PI3K/AKT/mTOR pathway and ferroptosis-related proteins to β-actin. Data are presented as mean ± SD. Representative blots from three independent experiments are shown. * p < 0.05, ** p < 0.01, *** p < 0.001 relative to the DGA group by ANOVA.


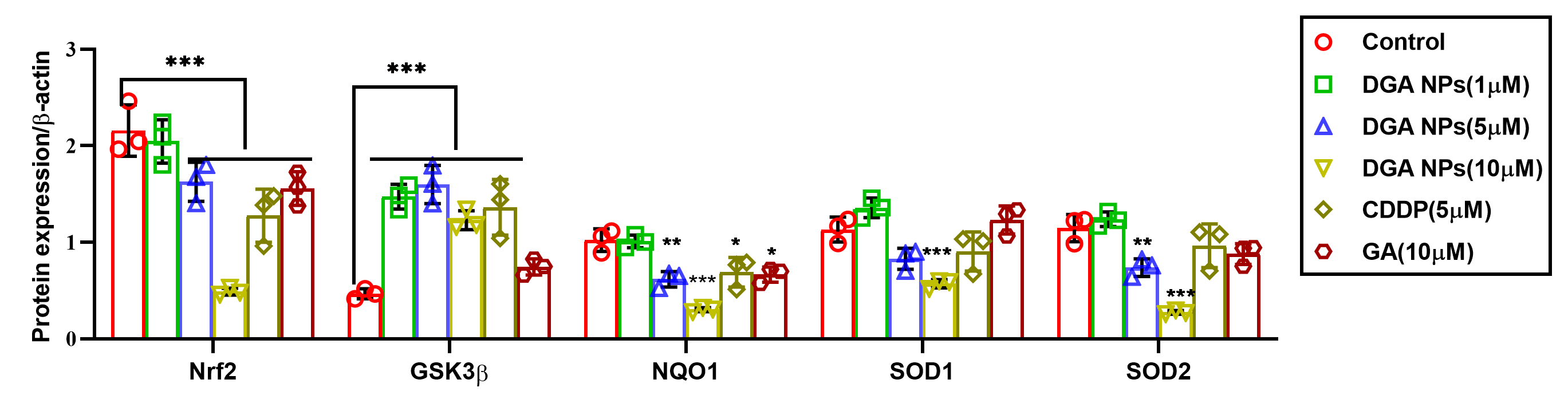
**Fig. S42.** Relative density of Nrf2 pathway related proteins to β-actin. Data are presented as mean ± SD. Representative blots from three independent experiments are shown. *p < 0.05, **p < 0.01, ***p < 0.001 relative to the control group by ANOVA.


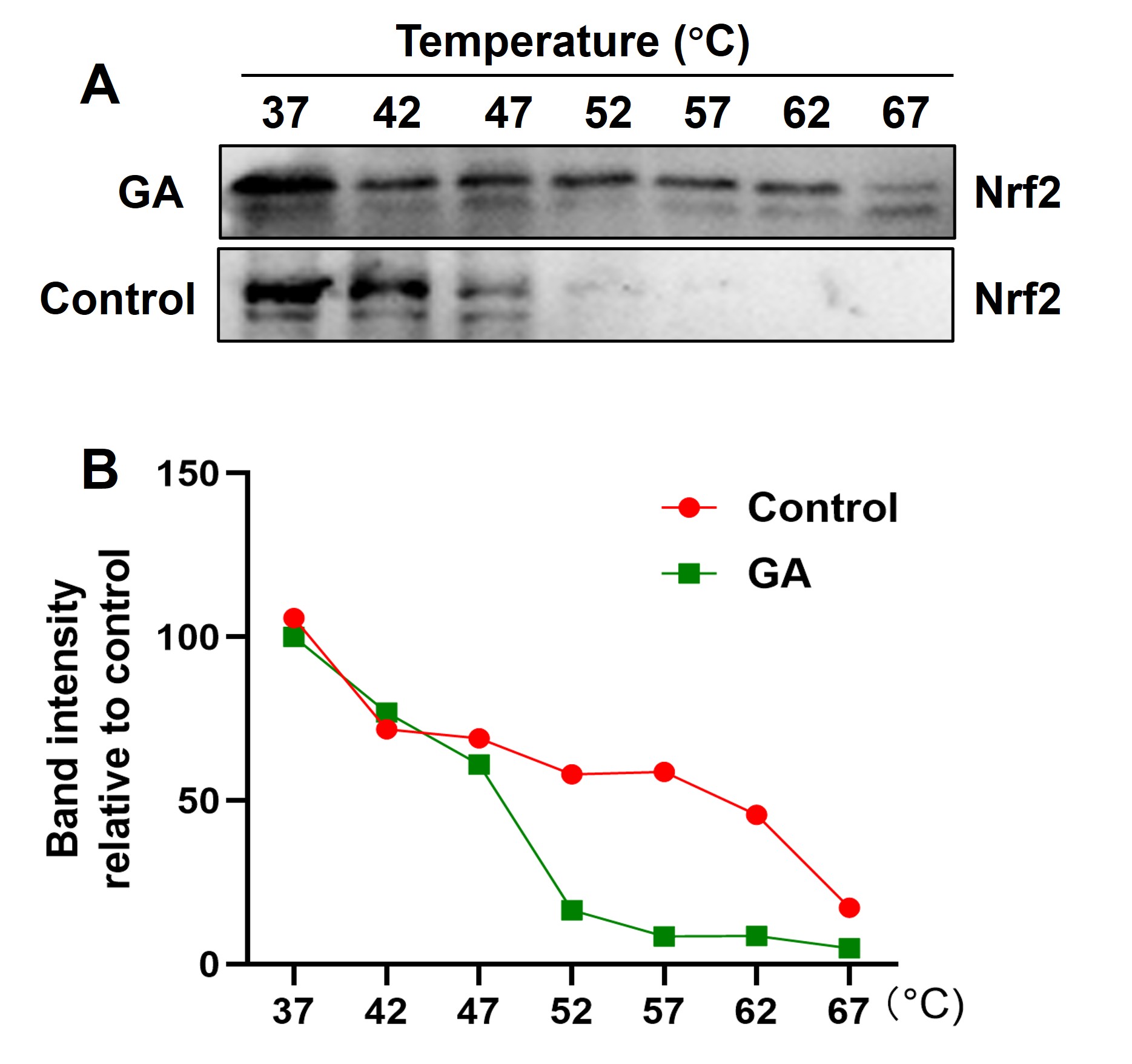
**Fig. S43**. CETSA analyzed the thermal stabilization of Nrf2 with GA (20 μM) in HepG2 cell lysates. (A) Western blotting assay of Nrf2. (B) CETSA melting curve.

**
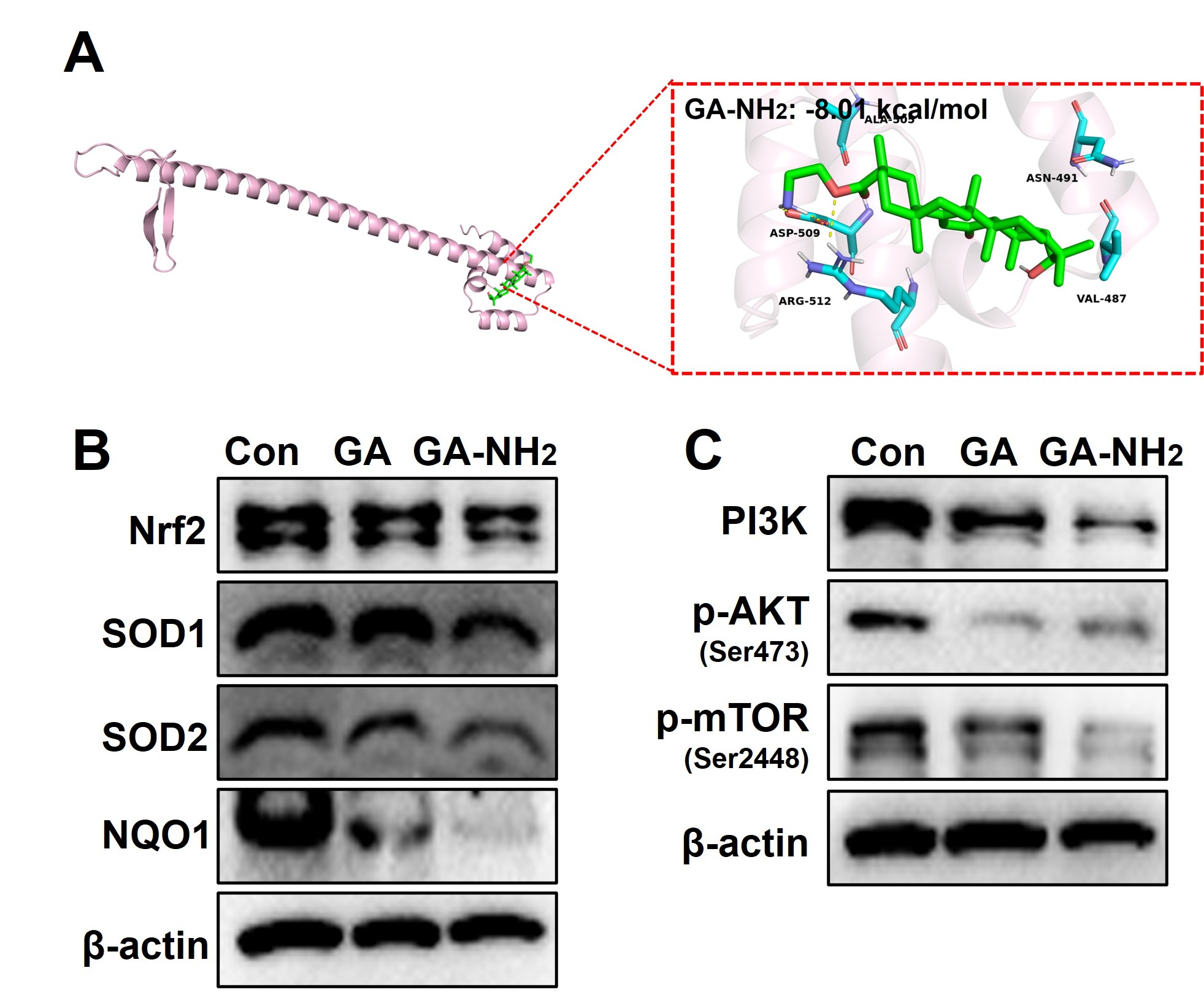
Fig. S44**. (A) The molecular docking model of GA-NH_2_ and Nrf2 protein. (B) Western blot assay of proteins related to the Nrf2 Pathway after 48h treatment with GA (20 μM) and GA-NH_2_ (20 μM). (C) Western blot assay of PI3K/AKT/mTOR pathway expression after 48 h treatment with GA (20 μM) and GA-NH_2_ (20 μM). (n = 3 biologically independent experiments). *p < 0.05 by ANOVA.


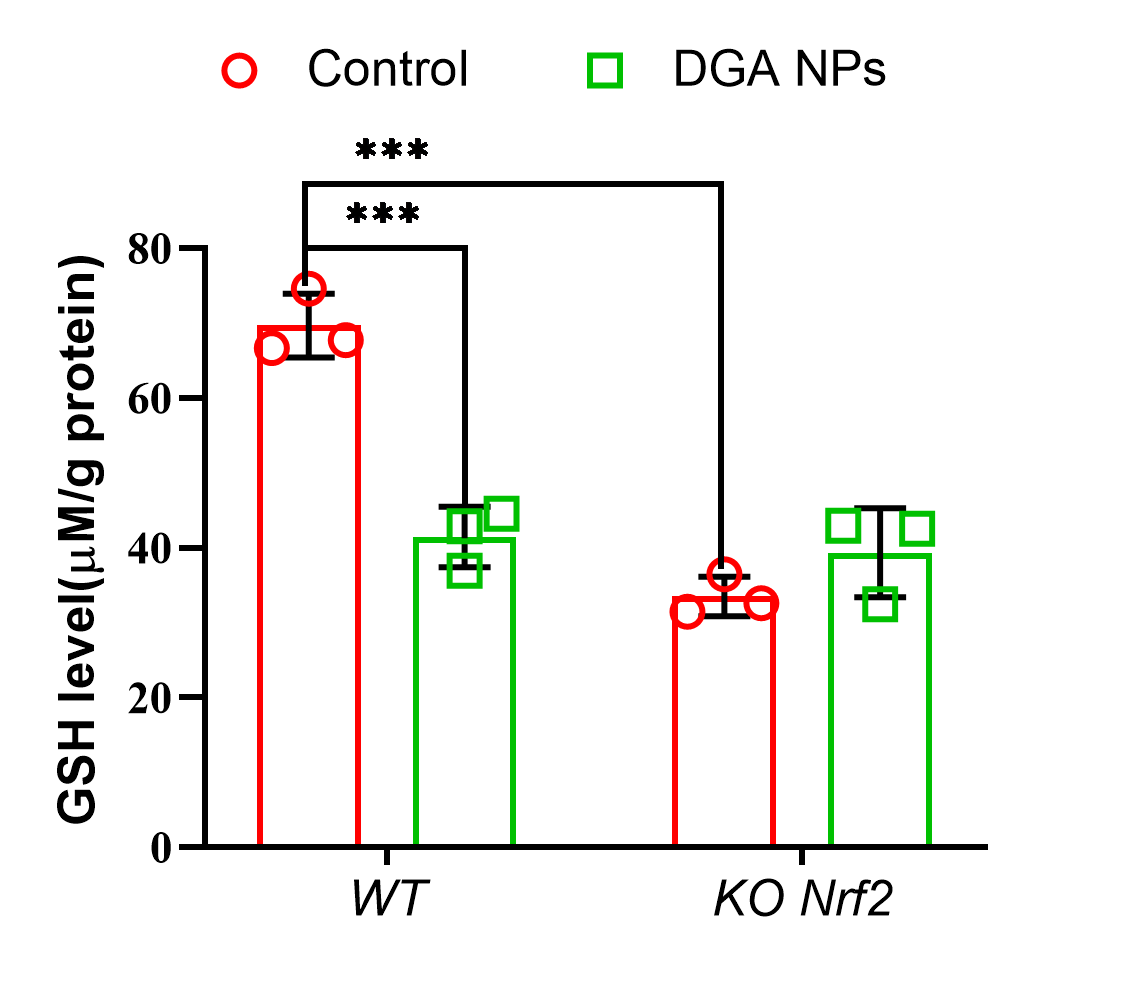
**Fig. S45**. Intracellular GSH levels in *WT* and *KO-Nrf2* cells treated with **DGA** NPs (5 μM) for 48h.

**
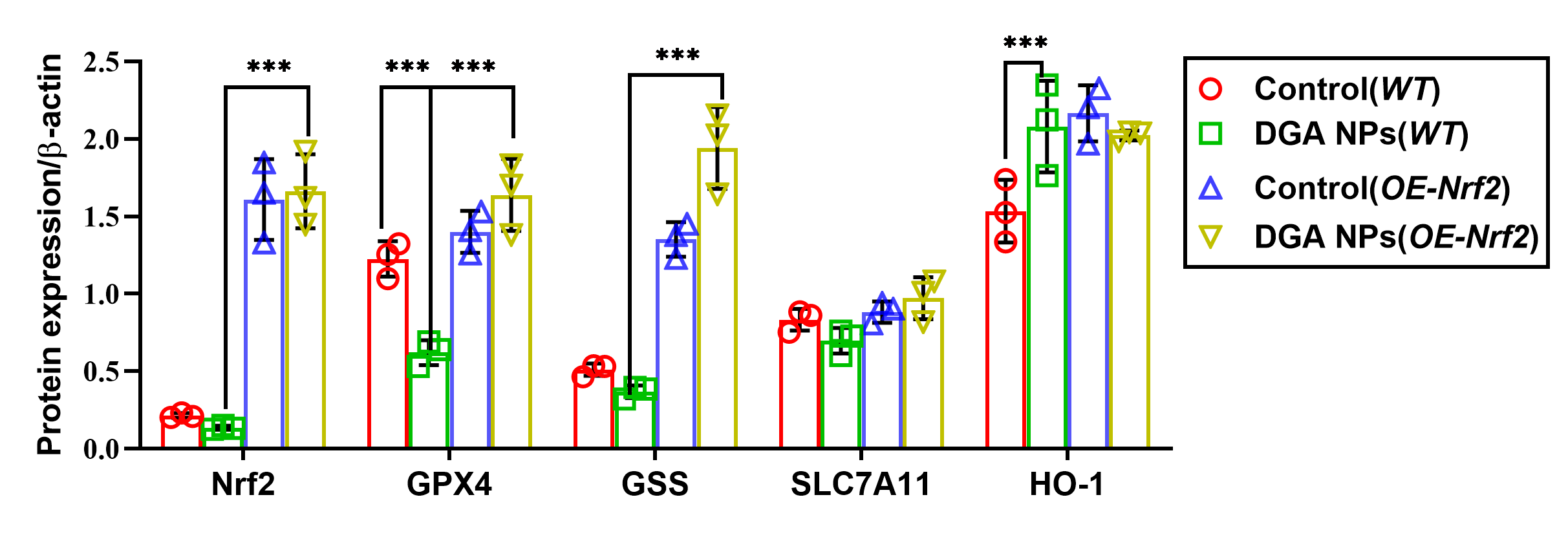
Fig. S46.** Relative density of Nrf2 and ferroptosis-related proteins to β-actin. Data are presented as mean ± SD. Representative blots from three independent experiments are shown. ***p < 0.001 relative to the control (*WT*) or **DGA** NPs (*WT*) group by ANOVA.


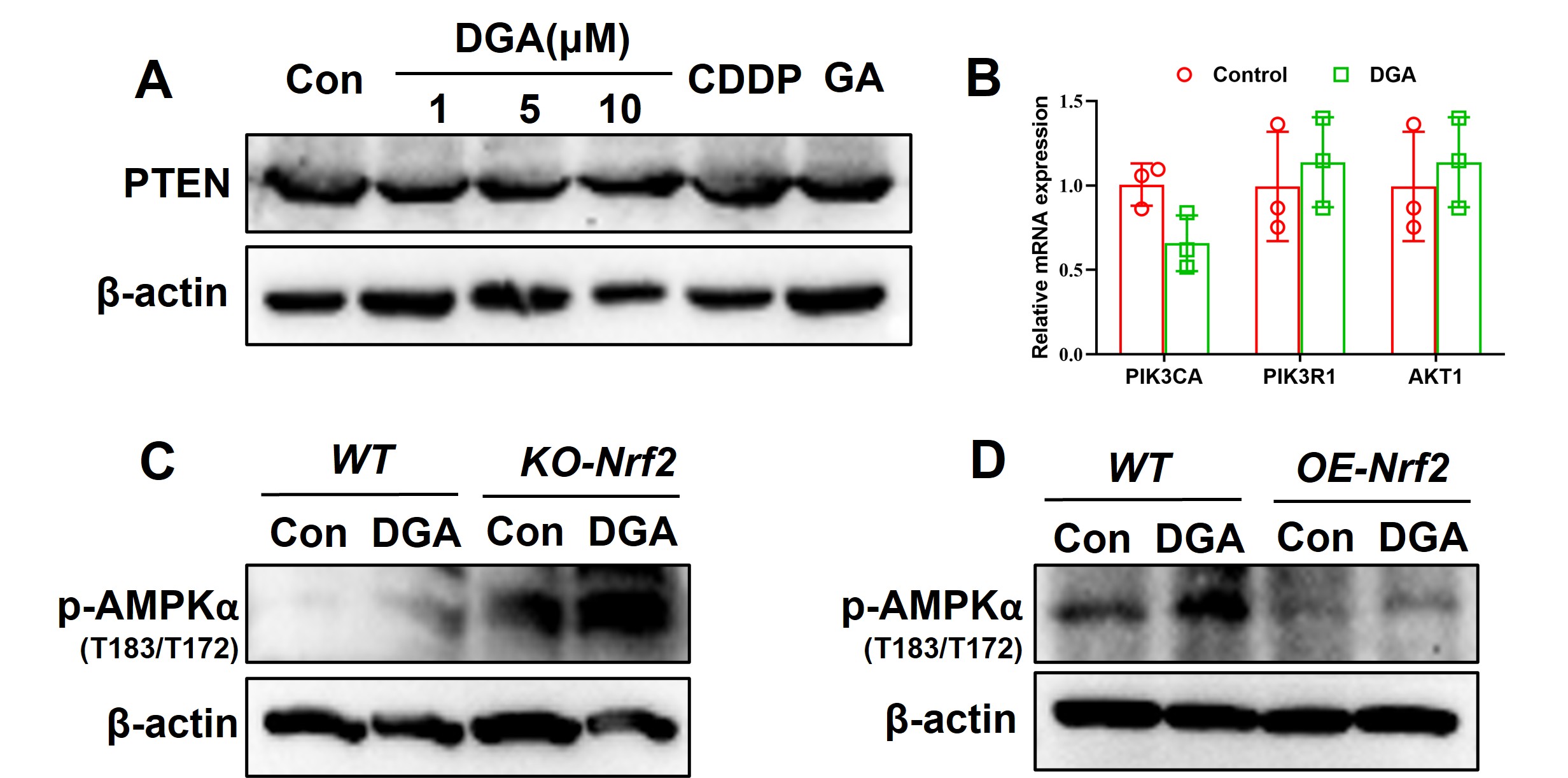
**Fig. S47. (A)** Western blot analysis of PTEN. **(B)** qPCR assay of PIK3CA, PIK3R1and AKT1 after 48h treatment with **DGA** NPs (5 μM) on HepG2 cells. **(C)** Western blot analysis of p-AMPK after 48h treatment with **DGA** NPs (5 μM) on *WT* and *KO-Nrf2* cells. **(D)** Western blot analysis of p-AMPK after 48h treatment with **DGA** NPs (5 μM) on *WT* and *OE-Nrf2* cells. Data are presented as mean ± SD (n = 3 biologically independent experiments).


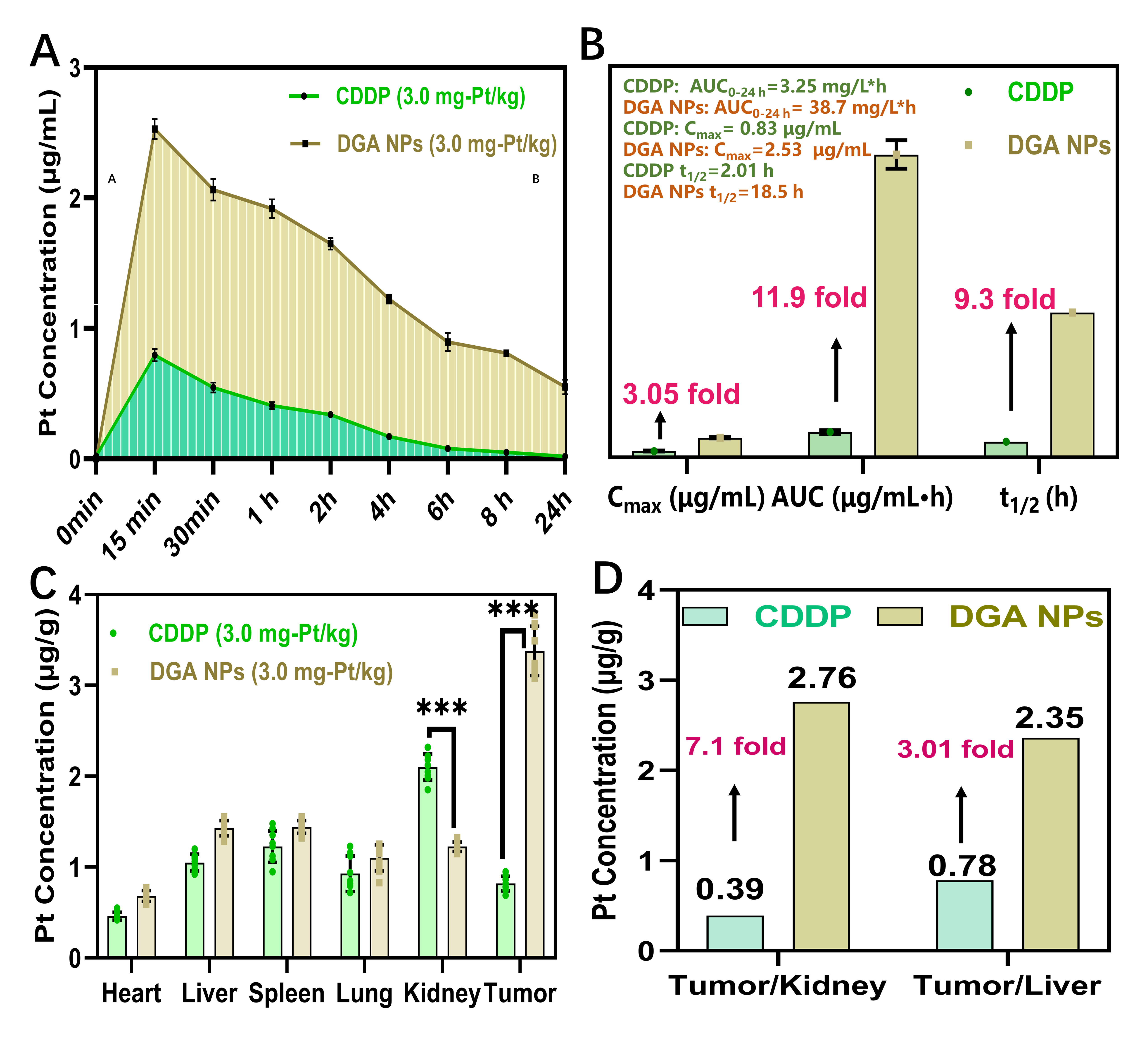
**Fig. S48**. **(A)** Plasma concentration-time curves of CDDP and **DGA** NPs after a single intravenous administration in mice. **(B)** Comparative pharmacokinetic parameters (C_max_, AUC, t_1/2_) of CDDP and **DGA** NPs. **(C)** Biodistribution of platinum in major organs and tumors at 24 hours post-administration. **(D)** Tumor/kidney and tumor/liver platinum ratios for CDDP and **DGA** NPs. Data are presented as mean ± SD. Statistical significance was determined by one-way ANOVA (*p < 0.05, **p < 0.01, ***p < 0.001; NS, not significant).

**Table S5**. Body weight (g) of control group mice during the treatment period.

|  | Control 1 | Control 2 | Control 3 | Control 4 | / |
| --- | --- | --- | --- | --- | --- |
| D0 | 20.7 | 20.5 | 19.6 | 20.9 | / |
| D2 | 20.1 | 21 | 20.3 | 21.3 | / |
| D4 | 19.7 | 20.7 | 19.9 | 22.3 | / |
| D6 | 19.9 | 21.4 | 20.8 | 23.9 | / |
| D8 | 20.2 | 22.5 | 21.6 | 23.2 | / |
| D10 | 21.3 | 21.6 | 22.8 | 24.1 | / |
| D12 | 22.4 | 22.6 | 21.9 | 25.2 | / |
| D14 | 21.8 | 23.2 | 22.5 | 24.8 | / |
| D16 | 22.3 | 23.5 | 21.6 | 25.2 | / |
| D18 | 21.9 | 24.1 | 22.3 | 24.3 | / |
| D20 | 23.1 | 24.3 | 22.7 | 24.8 | / |

**Table S6.** Body weight (g) of CDDP group mice during the treatment period.

|  | CDDP1 | CDDP 2 | CDDP 3 | CDDP 4 | CDDP 5 |
| --- | --- | --- | --- | --- | --- |
| D0 | 20.2 | 21.8 | 20.5 | 20.9 | 19.4 |
| D2 | 20.7 | 21.2 | 19.7 | 18.5 | 20.1 |
| D4 | 20.5 | 21.5 | 19.1 | 18.9 | 19.5 |
| D6 | 21.9 | 21.4 | 20.2 | 18.7 | 20.1 |
| D8 | 21.8 | 21.6 | 20.4 | 19.7 | 19.8 |
| D10 | 22 | 21.9 | 20.5 | 20.1 | 21 |
| D12 | 22.1 | 22.5 | 20.7 | 20.1 | 22.3 |
| D14 | 21.9 | 22.7 | 21.3 | 21.5 | 22.7 |
| D16 | 22 | 22.5 | 21.7 | 21.9 | 23.3 |
| D18 | 22.1 | 22.9 | 22.2 | 22.3 | 23.7 |
| D20 | 22.2 | 22.5 | 22.3 | 22.5 | 23.7 |

**Table S7**. Body weight (g) of CDDP combine GA group mice during the treatment period.

|  | CDDP+GA 1 | CDDP+GA 2 | CDDP+GA 3 | CDDP+GA 4 | CDDP+GA 5 |
| --- | --- | --- | --- | --- | --- |
| D0 | 20.3 | 20.7 | 21.3 | 18.9 | 20.8 |
| D2 | 20 | 20.5 | 20.8 | 19.3 | 19.2 |
| D4 | 20.3 | 20.7 | 21.5 | 19.1 | 19.7 |
| D6 | 21.2 | 21.3 | 22.2 | 19.8 | 20 |
| D8 | 21.8 | 21.2 | 21.4 | 18.7 | 20.5 |
| D10 | 21.5 | 19.8 | 21.9 | 19.2 | 20 |
| D12 | 20.8 | 20.8 | 22.5 | 20.1 | 21.7 |
| D14 | 21.9 | 19.7 | 23.6 | 21.3 | 22.5 |
| D16 | 22.5 | 20.1 | 22.5 | 22.3 | 21.6 |
| D18 | 22.7 | 19.5 | 23.1 | 21.6 | 23.5 |
| D20 | 21.1 | 19.7 | 21.7 | 22.7 | 21.9 |

**Table S8.** Body weight (g) of DGA NPs group mice during the treatment period.

|  | DGA 1 | DGA 2 | DGA 3 | DGA 4 | DGA 5 |
| --- | --- | --- | --- | --- | --- |
| D0 | 18.7 | 20.1 | 19.9 | 20.3 | 19.7 |
| D2 | 19.6 | 20.5 | 20.5 | 20.7 | 20.3 |
| D4 | 20.3 | 20.7 | 20.2 | 20.3 | 20.6 |
| D6 | 21.2 | 21 | 21.8 | 21.2 | 21.2 |
| D8 | 20.7 | 22.2 | 20.9 | 22.5 | 22.5 |
| D10 | 22 | 21.7 | 21.7 | 23.2 | 21.9 |
| D12 | 23.4 | 22.5 | 22.3 | 24.3 | 22.5 |
| D14 | 24.2 | 21.9 | 23.2 | 23.8 | 23.4 |
| D16 | 23.7 | 22.3 | 24.1 | 23.6 | 22.5 |
| D18 | 24.5 | 23.2 | 23.9 | 24.3 | 24.7 |
| D20 | 25.3 | 23.4 | 23.7 | 24.3 | 24.5 |

**Table S9**. Serum levels of kidney function markers (BUN, mmol/L; CRE, μmol/L) and liver enzymes (ALT, U/L; AST, U/L).

|  | Control | CDDP | CDDP+GA | DGA |
| --- | --- | --- | --- | --- |
| 1 (BUN) | 8.876117 | 10.73436 | 9.12516 | 7.171137 |
| 2 (BUN) | 8.703704 | 12.03065 | 9.048531 | 7.624521 |
| 3 (BUN) | 8.748404 | 11.87101 | 8.671775 | 7.203065 |
| 4 (BUN) | 7.330779 | 15.447 | 8.94636 | 8.218391 |
| 5 (BUN) | / | 9.425287 | 8.57599 | 9.195402 |
| 1 (CRE) | 11.52298 | 29.24685 | 13.45307 | 12.66456 |
| 2 (CRE) | 17.16026 | 23.97441 | 17.24264 | 20.98513 |
| 3 (CRE) | 14.19451 | 15.73623 | 16.13637 | 13.9709 |
| 4 (CRE) | 10.18134 | 13.66491 | 19.05504 | 17.67808 |
| 5 (CRE) | / | 18.57252 | 19.36103 | 14.64172 |
| 1 (ALT) | 18.36364 | 16.40909 | 17.40909 | 14.68182 |
| 2 (ALT) | 12.95455 | 26.77273 | 37.68182 | 19.54545 |
| 3 (ALT) | 13.36364 | 26.90909 | 12.09091 | 8.181818 |
| 4 (ALT) | 8.181818 | 29.50005 | 27.22727 | 24.77273 |
| 5 (ALT) | / | 30.95455 | 38.63636 | 16.36364 |
| 1 (AST) | 72.88889 | 145.6667 | 120.8889 | 87.88889 |
| 2 (AST) | 86.44444 | 112.2222 | 136.7778 | 101.5556 |
| 3 (AST) | 75.77778 | 97 | 70.11111 | 110.8889 |
| 4 (AST) | 54.44444 | 91.88889 | 109.7778 | 126.5556 |
| 5 (AST) | / | 86.22222 | 111.6667 | 83.66667 |
